# Supplementary material for: Zebrafish behavioural profiling identifies GABA and serotonin receptor ligands related to sedation and paradoxical excitation
Source: Nat Commun. 2019 Sep 9;10:4078. doi: 10.1038/s41467-019-11936-w (PMC6733874; doi:10.1038/s41467-019-11936-w)
Supplement: Supplementary file 1 — Supplementary Information [file 41467_2019_11936_MOESM1_ESM.pdf]

## SUPPLEMENTARY INFORMATION

Zebrafish behavioral profiling identifies ligands, targets, and neurons related to sedation and paradoxical excitation

## AUTHORS

Mc Carroll et al.

## SUPPLEMENTARY NOTE 1

**$GABA_AR$ ligands produce paradoxical excitation in zebrafish.** Compounds with weak phenoscores (x < 0.51) included one $GABA_B$ receptor agonist, one PAM of δ-subunit containing $GABA_ARs$, two non-BZ-site ligands, three structurally-related $GABA_AR$ orthosteric agonists, and seven BZ-site $GABA_AR$ PAMs (**Fig. 1g**). For these compounds, the average phenoscores were significantly less than the positive controls ($P < 0.01$, Kolmogorov-Smirnov test, **Supplementary Figure 20a**), suggesting that these compounds did not phenocopy etomidate. For example, the highest scoring ocinaplon treatment produced a behavioral profile that resembled the negative controls (**Supplementary Figure 2**). These data suggest that a variety of GABAergic compounds do not cause sedation and paradoxical excitation.

Compounds with intermediate phenoscores (0.51 < x < 0.71) included several types of $GABA_AR$ PAMs including thiopental, carboetomidate, THDOC, alfaxalone, diazepam, and valerenic acid. The highest scoring profiles produced by some of these compounds (including alfaxalone, thiopental, and tracazolate) showed a barely detectable statistically significant difference compared to the positive controls (0.01 < P < 0.05). The highest scoring profiles of animals treated with diazepam, and valerenic acid were significantly lower than the positive controls ($P < 0.01$, Kolmogorov-Smirnov test, **Supplementary Figure 20a**), however these treatments produced interesting intermediate effects on sedation and paradoxical excitation. For example, although the highest-scoring diazepam treatment was strongly sedating in most assays, it produced eASRs that were relatively weak and inconsistent (**Supplementary Figure 2**). These data suggest that a variety of PAMs have intermediate effects on sedation and paradoxical excitation.

Interestingly, although DOC and progesterone are neurosteroid precursors, they were among the most potent compounds tested (**Fig. 1g)**. As expected, progesterone's etomidate-like phenotype was suppressed by dutasteride, a 5-alpha-reductase inhibitor that blocks the metabolic conversion of progesterone to allopregnanolone, suggesting that these compounds were converted to active neurosteroids (**Supplementary Figure 5**).

## SUPPLEMENTARY NOTE 2

**Target prediction using SEA.** We used the Similarity Ensemble Approach (SEA) to predict targets based on 'guilt-by-association' enrichment factor scores (EFs). These EFs were first developed for predicting adverse drug interactions [1], and balance the overall strength of a given target-to-compound-set association by correcting for the frequency that specific targets are predicted over random compounds sets in the screen [2]. Here, we used EFs to predict targets for the compounds that caused eASRs in the zebrafish.

SEA identified 15 compounds with enriched target predictions for mGluRs (**Supplementary Table 9**, **Supplementary Figure 12a**). We chose eight of these compounds to reorder and retest and found that four of them reproducibly caused eASRs *in vivo* (**Supplementary Figure 12b, Supplementary Table 9**). Next, we tested five of these compounds as agonists and antagonists for activity at seven human mGluRs (mGluR1-6 and mGluR8). However, none of the compounds showed strong functional effects against mGluRs *in vitro* (**Supplementary Figure 12c**), suggesting that the

compounds did not act via mGluRs *in vivo*. To further test the mGluR hypothesis, we tried to phenocopy etomidate in dose-response experiments with a panel of structurally-diverse mGluR ligands and ligand combinations (**Supplementary Table 9**). Although MPEP, a mGluR5 antagonist, reproducibly caused eASRs, MPEP-induced eASRs were substantially lower in magnitude than etomidate-induced eASRs, and MPEP-induced eASRs only occurred in a narrow concentration range (**Supplementary Figure 12b, Supplementary Table 9**). Therefore, although MPEP weakly phenocopied etomidate, we found no further evidence that hit compounds targeted mGluRs, as predicted by SEA.

SEA predicted that $GABA_AR$ was a target of four hit compounds (**Supplementary Figure 11**). We tested three of them (5658603, 5142031 and 7145248) and found that one (5658603) potentiated $GABA_AR$ *in vitro* (**Fig. 2f, red arrow)**. Curiously, we noted that SEA failed to predict $GABA_AR$ as a target for most hit compounds that tested positive in the $GABA_AR$ FLIPR assay (**Fig. 2f**), underscoring the value of behavior-based screens for identifying bioactive compounds with poorly annotated chemical structures.

## SUPPLEMENTARY NOTE 3

### $GABA_A$R and HTR6 ligands likely converge on a common neural substrate

To determine if HTR6 antagonists activated the same neurons as GABAergic ligands, we took three approaches. First, we looked for overlap between 5HT immunohistochemistry and the eASR substrate neurons (**Supplementary Figure 13f**). Consistent with previous reports, we observed strong 5-HT staining in the telencephalon, pineal gland, hindbrain, and dorsal raphe nuclei [3]. In addition, we observed bilateral 5-HT staining in tracts that converged on the midline of the caudal hindbrain at the same location of the putative eASR substrate neurons in the caudal hindbrain (**Supplementary Figure 13f**). These tracts likely originated from the dorsal raphe, but we could not trace their origin definitively. Second, we visualized HTR6 mRNA expression by RNAscope but could not detect reproducible expression patterns (**Supplementary Figure 14**), suggesting that HTR6 mRNA is not abundantly expressed. Finally, we tested for pharmacological interactions between GABAergic and serotonergic ligands. As expected, pretreatment with the $GABA_AR$ antagonist PTX rescued etomidate-treated animals, increasing and decreasing the magnitude of the violet light and eASR phenotypes, respectively (**Supplementary Figure 15a)**. Similarly, PTX rescued the GABAergic compound 5658603, and partially rescued compounds 701338, and 5942595, albeit to a lesser extent than etomidate (**Supplementary Figure 15a)**. PTX also partially rescued the behavioral phenotypes of several HTR6 antagonists including BGC 20-761, 6029941, 6028165, 6030006, and 6013263 (**Supplementary Figure 15b**). By contrast, EMDT oxalate, a HTR6 agonist, did not suppress eASRs caused by HTR6 antagonists (**Supplementary Figure 15c**), suggesting that the effects of HTR6 antagonists are not easily reversed. Together, these data suggest that $GABA_AR$ agonists and HTR6 antagonists likely cause eASR behaviors via different targets that converge on a common neural substrate in the zebrafish hindbrain.

## SUPPLEMENTARY DISCUSSION

Although etomidate and propofol are human anesthetics, the hit compounds identified in this study

may not be useful as human anesthetics. One reason, is that the primary screen in zebrafish did not include behavioral correlates key anesthetic effects including analgesia and amnesia. Furthermore, only a minority of the hit compounds suppressed the TrpA1-induced pain-related assay (**Fig. 6b**), suggesting that many of the compounds may not cause the analgesic effects associated with human anesthesia. Another reason is hat paradoxical excitation is an unwanted side effect of anesthetic drugs. Even if the hit compounds translated to mammals (causing both sedation and paradoxical excitation), additional studies would be necessary to determine if the paradoxical excitation phenotype could be overcome at higher concentrations or via medicinal chemistry. For example, the 21 analogs of compound 7013338 showed variable efficacies (**Fig. 6e**), suggesting that it may be possible to use medicinal chemistry to increase or decrease eASR activity. These shifts likely correspond specific effects on one or more molecular targets that would need to be identified with more sensitive functional assays, such as electrophysiological experiments, of recombinantly expressed receptor subtypes. Future studies may seek to identify different ligands that sedate zebrafish without causing eASRs, or eASRs may be used as a counter screen for other potential anesthetic lead compounds. Presumably, such compounds would work through different mechanisms than etomidate, propofol, and the other compounds identified in this study, and would further improve our understanding of GABAergic signaling, anesthesia, and paradoxical excitation.

## SUPPLEMENTARY METHODS

**Behavioral assays for pERK.** Animals were treated with DMSO or drug for one hour then exposed to a low-volume acoustic stimulus every 10 seconds for 10 minutes. Optovin-treated animals were stimulated with violet light for one second every 10 seconds for 10 minutes. Videos were recorded to measure response to the stimulus and quantified by motion index (MI). Animals were immediately fixed in 4% PFA in PBS.

**High speed imaging.** Digital video was recorded at 500-1000 frames per second using an Integrated Design Tools NX5-S1 digital camera. Assay duration was 500-1000 ms. Low amplitude tap stimuli was delivered as described above. Videos and still images were assembled using Fiji (imageJ) and Photoshop (Adobe).

**RNAscope.** Zebrafish (6 dpf) were fixed using 10% normal buffered formaldehyde overnight at 4˚C, dehydrated in 100% methanol and stored at -20˚C until the assay was performed. Fluorescent in situ hybridization was performed using the RNAScope kit (Advanced Cell Diagnostics)[4] with the following modifications: target retrieval was performed using 150 mM Tris pH 9.5 for 15 minutes at 70˚C, washes were performed using 0.2x SSCT (0.01% Tween-20, 3mM NaCl, 0.3mM TriNa-citrate, pH7), samples were stored overnight after probe hybridization and 2x 5 minute washes in 5x SSC (75 mM NaCl, 7.5 mM TriNa-citrate, pH7) at room temperature before amplification and detection steps were performed the following day.

**Viability.** Animals were scored manually to determine viability after anesthetic treatments. 100 animals were scored per condition at 10 minutes, 1 hour and 5 hours post treatment. Strong heart rate was observed under a dissecting scope (Leica M-80) for our viability score.

**Structural Clustering.** Structural clustering was performed on the top 125 hit compounds using the rdkit function FingerprintMols package written for python. Tanimoto similarity function was used with a

threshold of 0.25 to define clusters and visualized using the scipy hierarchy dendrogram function.

**SEA and EF calculations.** Here we describe our computational pipeline: 1) Use the reference trace to discover the top 125 hit compounds (most similar phenotypically related to etomidate). 2) Organize hit compounds into hierarchical supersets of increasing numbers of hit compounds. Use SEA analysis to generate target predictions for each of the compounds in the sets. Perform enrichment factor calculations on the sets, which attempt to correct the occurrence of target predictions for a set of compounds by comparing to a background distribution.[2] To do so we generated 10,000 sets of 200 random screening compounds each, and applied the following formula to calculate the enrichment of target *y* for set *x*: E_xy = n*N / (A * T), where *n* is the number of times target *y* is predicted for set *x* compounds by SEA, A is the number of times any target shows up for set *x*, T is the number of times target *y* shows up for any set, and N is a normalization factor equal to the product of all the targets and all the sets.

**Determination of phenotypic thresholds and significance.** For each ligand, we selected the dose that gave the highest average phenoscore, and for that dose, we performed a two-sample Kolmogorov-Smirnov (KS) test to calculate the KS statistic against the 12 positive control replicates of etomidate @ 6.25 µM using the scipy function ks_2samp from the scipy.stats package (**Supplementary Figure 20a**).

To calculate approximate thresholds of phenoscore significance, we performed a statistical simulation. For each score in the space of possible phenoscores (binned in 0.05 increments from 0 to 1), we sampled 12 replicates from a uniform distribution centered around the score ranging from -4σ to +4σ away from the mean, and calculated the KS statistic against the etomidate 6.25 µM replicates. We repeated this simulated procedure 100 times to get robust statistics, and took the average of these *P* values. However, we realized that the standard deviation of replicates across different $GABA_AR$ ligands was not a constant value. It tended to be low for extremely poor phenotypes, peaked for intermediate phenotypes, and decreased again for extremely strong phenotypes. Therefore, we fit the standard deviations for $GABA_AR$ ligands as a function of phenoscore with a 10th order polynomial using the Polynomial package in numpy (**Supplementary Figure 20b**). Using this resulting polynomial, we calculated the KS *P* values from the simulated uniform distributions as we iteratively stepped along the y-axis ; these *P* values were smoothly distributed except for a discontinuity around phenoscore 0.5 due to rapidly increasing *P* values in this range (**Supplementary Figure 20c**). We derived the threshold phenoscores associated with these *P* values by fitting another polynomial to the resulting distribution in the smooth region (above phenoscore 0.5) (**Supplementary Figure 20d**) and calculating the roots of the function at those *P* values. The resulting phenoscores corresponding to 0.01 and 0.05 *P* value thresholds were 0.51 and 0.71, respectively.

**Z' and false positives and negatives calculation.** To calculate the Z' (quality of screen coefficient), we use the formula Z' = $1 - 3(\sigma_p + \sigma_n)/(\mu_p - \mu_n)$, expressed in terms of the mean and SD of the positive (p) and negative (n) controls. The false positive/negative rates were determined at a threshold of 3 SD. Any positive controls with a phenoscore 3 SD away from the positive control mean ($\mu_p$) were labeled false positives. Likewise, any negatives controls 3 SD away from the negative control mean ($\mu_n$) were labeled false negatives.

**General Synthesis Scheme of Isoflavones**

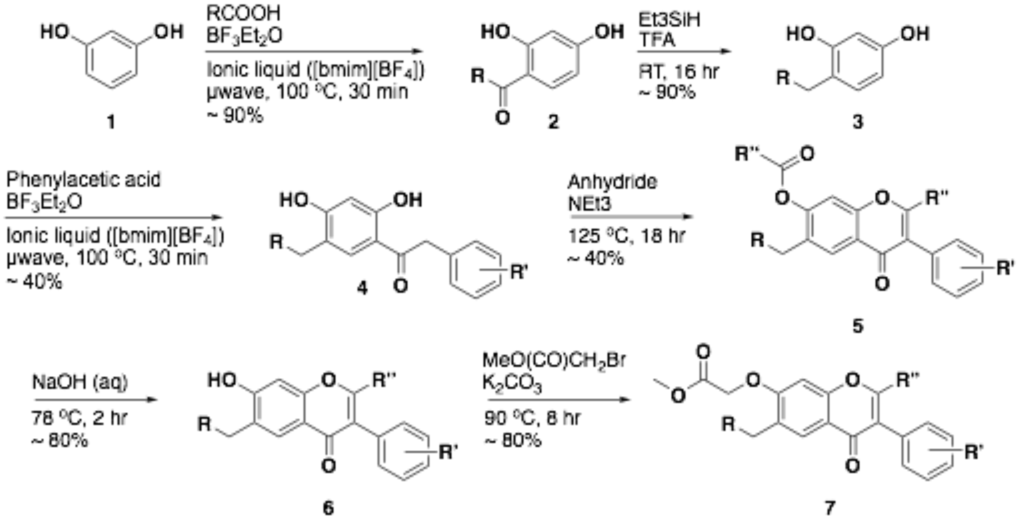


**General Procedure for the Synthesis of 2.** To a mixture of resorcinol (1 equiv) and carboxylic acid (1 equiv), BF3·Et2O (3 equiv) and ionic liquid ([bmim][BF4]) (3.5 equiv) was added. The reaction mixture was irradiated at 100 °C for 30 min in a microwave reactor at the maximum power of 300W. The solution was allowed to cool and poured into water. The reaction mixture was extracted with ethyl acetate, and the organic layer is separated and washed with brine, dried, and concentrated. The concentrate was purified by silica gel column chromatography to get ketone 2.

**General Procedure for the Synthesis of 3.** 2 was dissolved in trifluoroacetic acid (20 equiv) and triethylsilane (2.5 equiv) was added at room temperature. The resulting solution was stirred overnight, and the solvent was removed by flushing nitrogen gas in mild temperature. The residue was purified by silica gel column chromatography to get diol 3.

**General Procedure for the Synthesis of 4.** To a mixture of 3 and a phenylacetic acid (1 equiv), BF3·Et2O (3.5 equiv) and ionic liquid ([bmim][BF4]) (3 equiv) was added. The reaction mixture was irradiated at 100 °C for 30 min in a microwave reactor at the maximum power of 300W. The solution was allowed to cool and poured into water. The reaction mixture was extracted with ethyl acetate, and the organic layer was separated and washed with brine, dried and concentrated. The concentrate was purified by silica gel column chromatography to get ketone 4.

**General Procedure for the Synthesis of 5.** A mixture of 4, propionic anhydride (5 equiv) and triethylamine (4 equiv) was heated at 125 °C for 12 h. Then the reaction mixture was added to cold dilute 1M HCl solution. The mixture was extracted with ethyl acetate, and the organic layer was separated and washed with brine, dried and concentrated. The concentrate was purified by silica gel column chromatography to get isoflavone 5.

**General Procedure for the Synthesis of 6.** A solution of 5 in ethanol (0.2 M) containing 10% w/w NaOH was refluxed for 30 min. After 30 min, the same amount of water was added, and heating was continued for another 1.5 h. The reaction mixture was acidified with dilute hydrochloric acid and extracted with ethyl acetate, and the organic layer was separated and washed with brine, dried and concentrated. The concentrate was purified by silica gel column chromatography to get isoflavone 6.

**General Procedure for the Synthesis of 7.** To a solution of 6 in DMF (0.5 M), methyl bromoacetate (1.1 equiv) and $K_2CO_3$ (3 equiv) was added. The mixture was heated to 90 °C for 8 h. The reaction mixture was cooled to room temperature and extracted with ethyl acetate. The organic layer was

separated, washed with brine, and dried and concentrated. The concentrate was purified by silica gel column chromatography to get isoflavone 7.

**GABA$_A$R terminology.** Notation for GABA receptors conform to IUPHAR recommendations [5]. Receptor subunits are indicated by their greek symbols with subscripted numbers to indicate specific isoforms as in: "the $\alpha_1$ subunit isoform". To refer to GABA$_A$ receptor (GABA$_A$R) subtypes, the term GABA is used to indicate the receptor type, and the subscript A is used to refer to all GABA$_A$Rs. Subtypes comprised of specific subunit isoforms are indicated like: "the $\alpha_1\beta_2\gamma_2$ subtype".

## SUPPLEMENTARY FIGURES

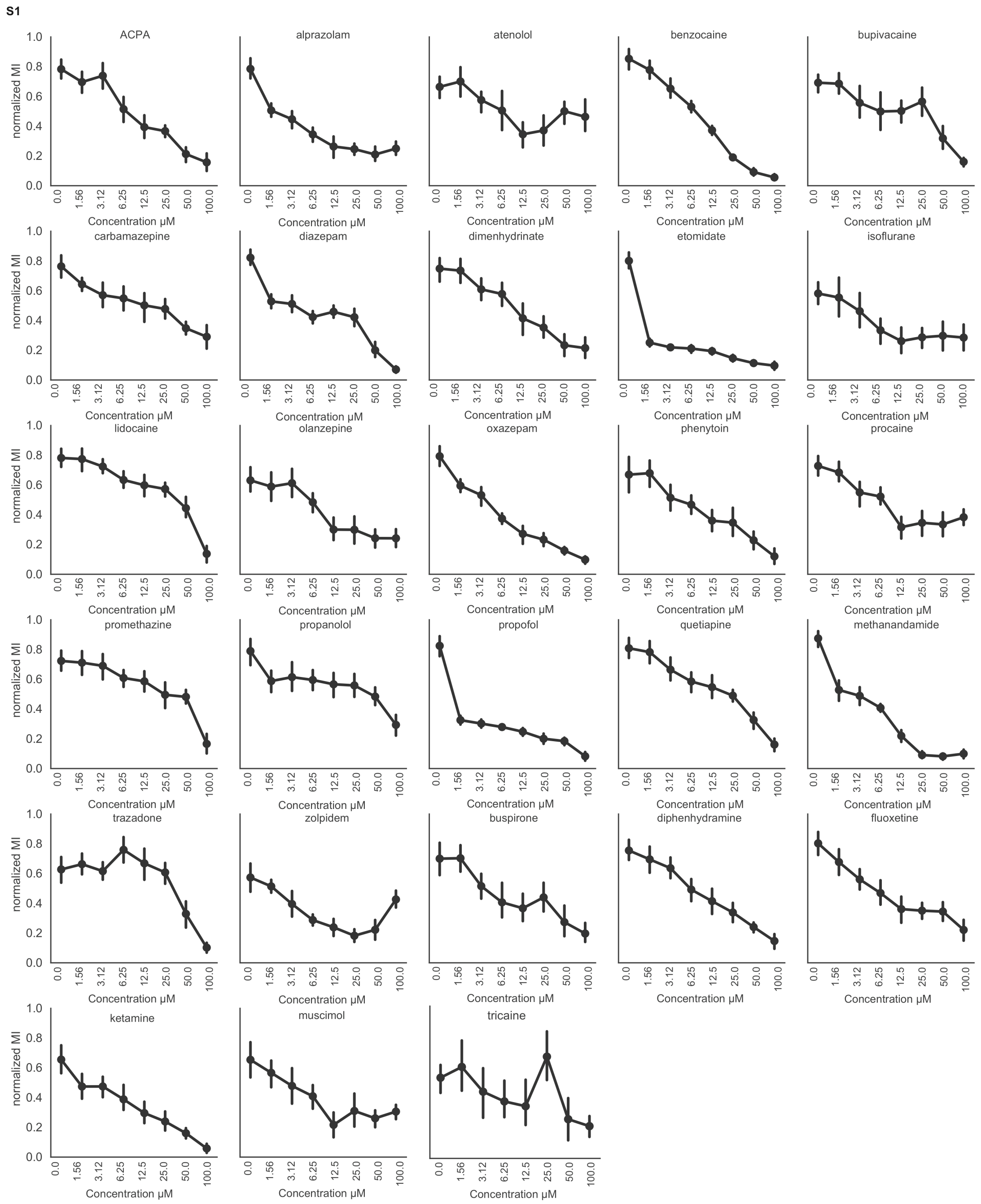


**Supplementary Figure 1. Sedatives cause a dose dependent reduction in zebrafish motion.** A panel of 30 known sedatives administered to 7dpf zebrafish larvae (n = 12 replicates; 96 fish/condition) at a 2 fold dilution series. The y-axis represents motion index (MI) and the x-axis represents dose.

## S2

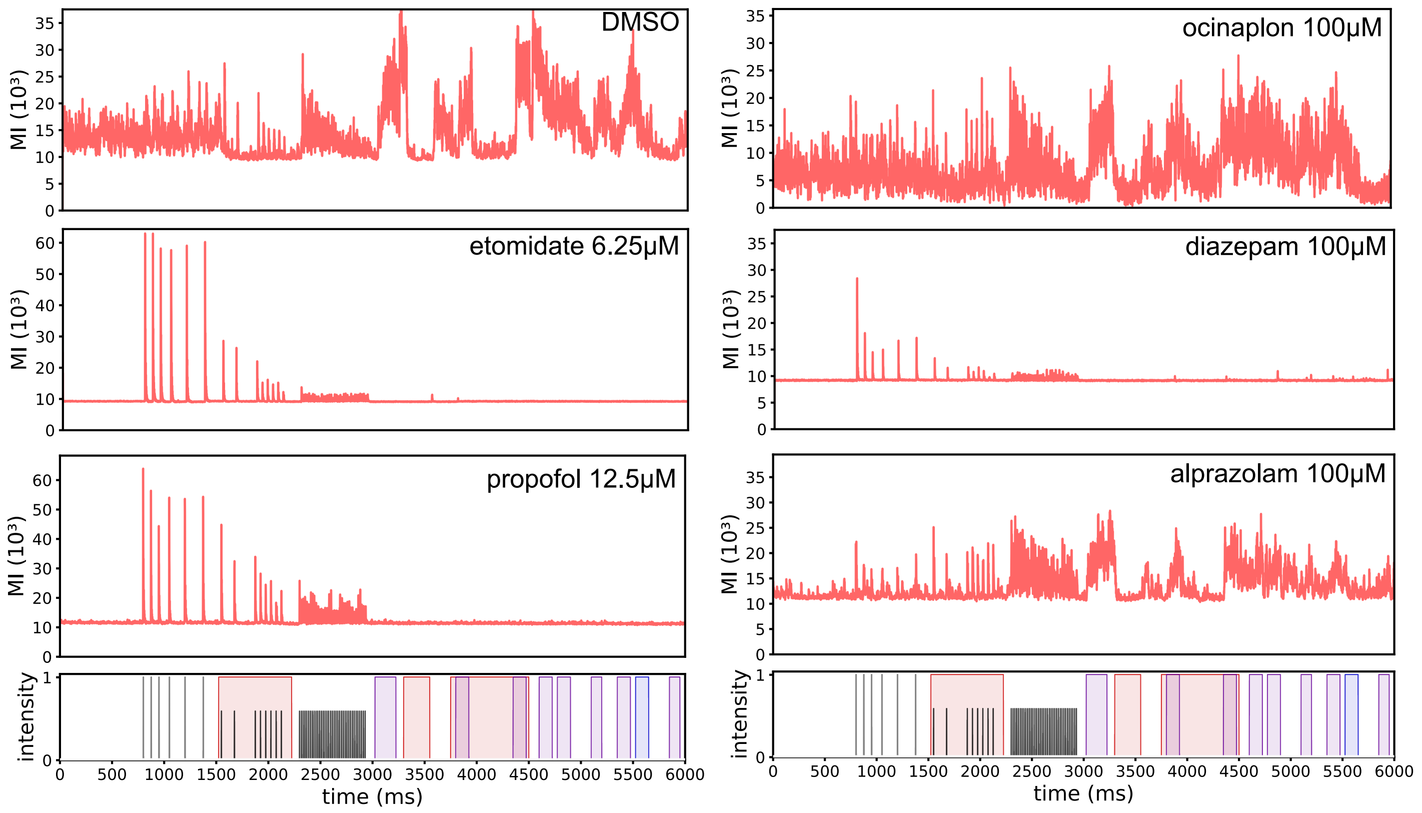


**Supplementary Figure 2. Propofol and etomidate block light-induced behaviors, but enhance the acoustic startle response.** The plots show motor activity (*y*-axis) of zebrafish treated with the indicated compounds (n = 12 wells). Colored bars above the *x*-axis represent the timing and duration of indicated stimuli.

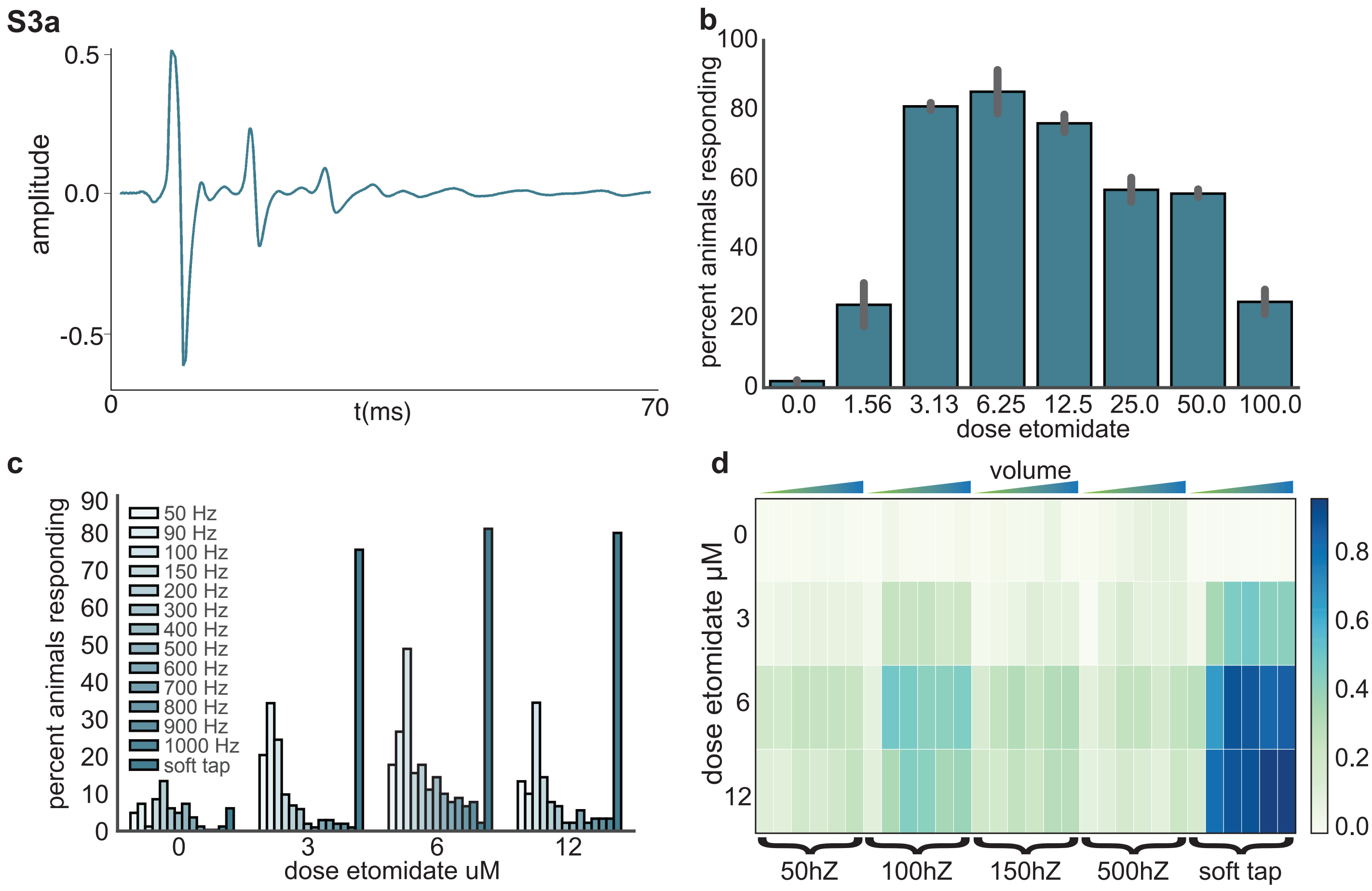


**Supplementary Figure 3. eASR stimulus characterization.** We explored a range of digital, acoustic stimuli to understand which parameters were important for triggering eASRs. **(a)** Recorded waveform of the dampened solenoid. The original stimulus, generated by a dampened solenoid, approximated a 100 Hz inverse fading sine wave, with a 70 dB maximum volume and 70 ms duration. **(b)** Bar graph depicting startle frequency (y-axis) of 100 animals to the dampened solenoid stimulus at increasing concentrations of etomidate (x-axis), it elicited responses in 85% of etomidate-treated animals (6.25 μM) and in 2% of controls. **(c)** Startle frequency (y-axis) of 100 animals treated with indicated concentrations of etomidate (x-axis) in response to different frequencies of synthesized and dampened solenoid acoustic stimulus (colored bars). In frequency scans from 50-1000 Hz, the highest magnitude eASRs were elicited by 100 Hz stimuli. Interestingly, the most effective synthesized stimulus (a 100 Hz inverse fading sine wave; 70ms) was only 50% as effective as the original solenoid, suggesting that some unknown feature of the original solenoid-based stimulus was not captured by the synthesized waveform and/or the surface transducers. **(d)** Heat map of the startle frequency of 100 animals (color bar) in response to increasing volume (top y-axis) of different frequency synthesized acoustic stimulus and the solenoid stimulus (x-axis). Animals were treated with increasing concentrations of etomidate (y-axis). At 100Hz, all stimuli greater than 60 dB were effective, whereas those less than 55 dB were not.

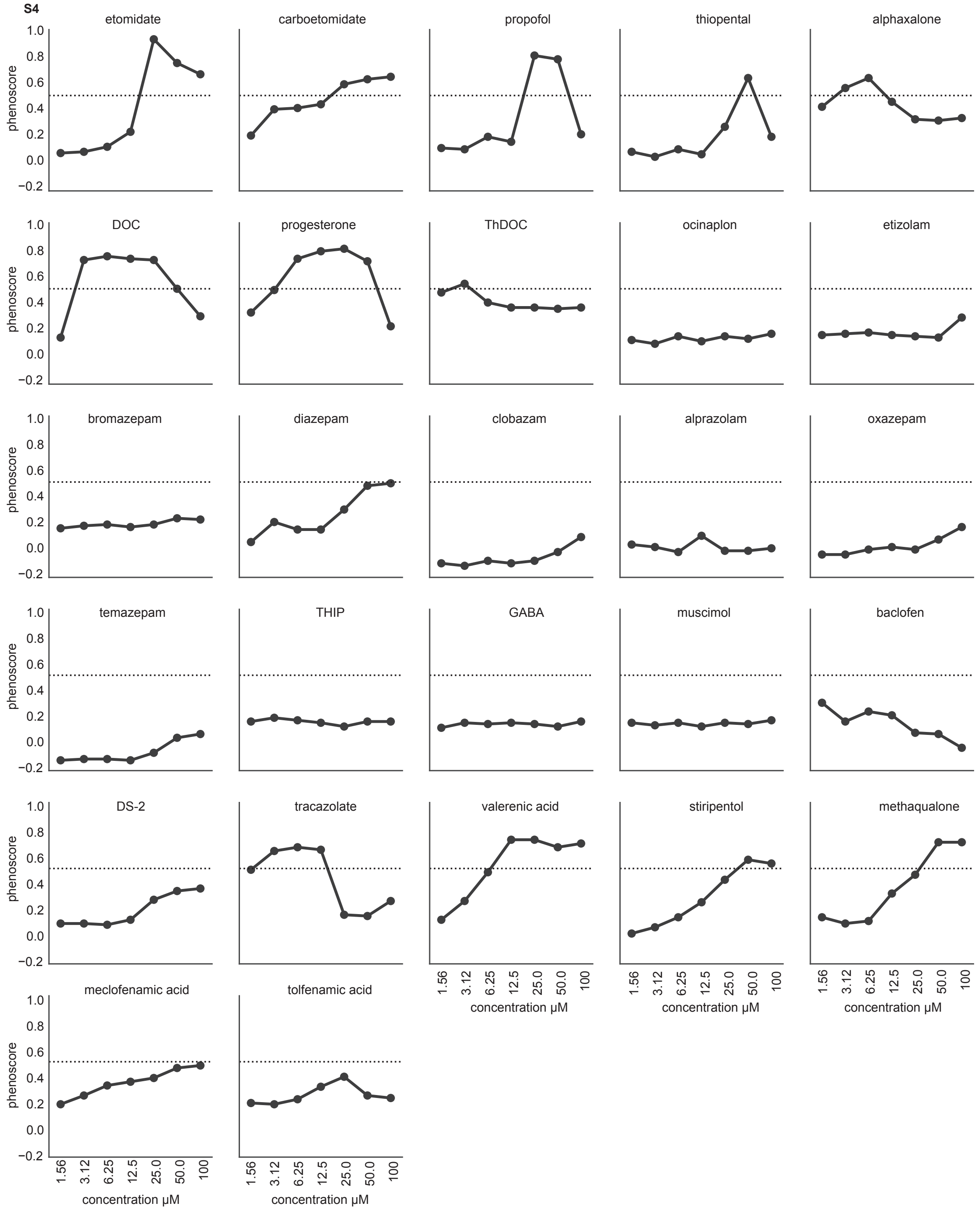


**Supplementary Figure 4. Dose response analysis of GABA reference compounds.** Average phenoscores (*y*-axis) of zebrafish treated with the indicated compounds (n = 12 wells) at increasing concentrations (x-axis).

S5

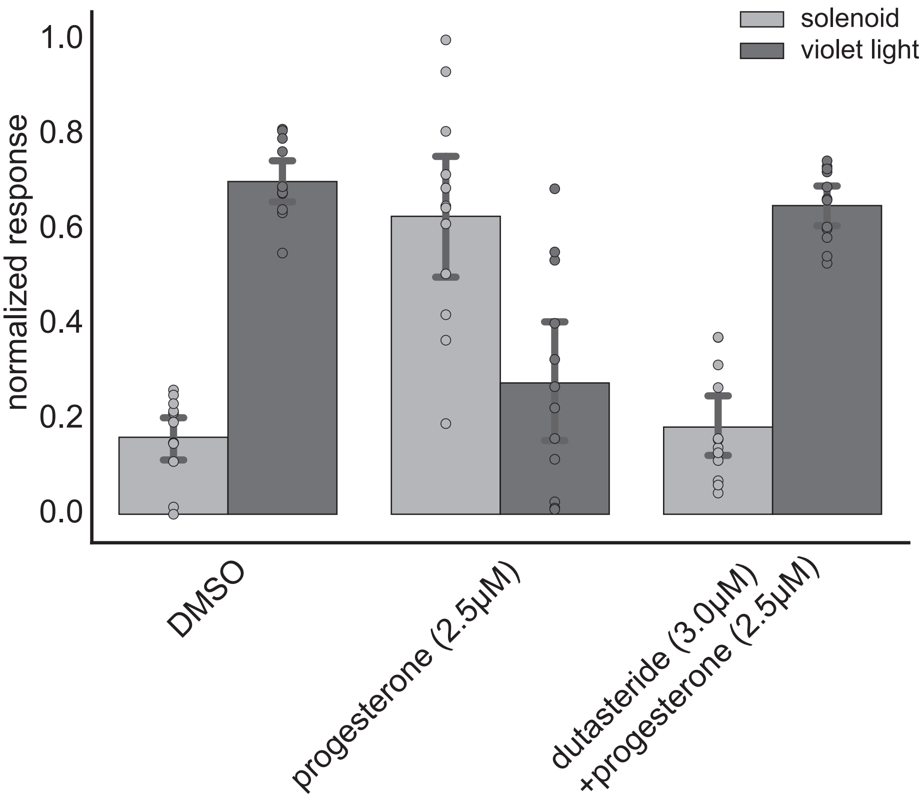


**Supplementary Figure 5.**

**Dutasteride inhibits progesterone-induced eASRs.** The plots show the normalized behavioral responses (y-axis), to acoustic (grey) or light (black) stimuli, in animals treated with the indicated compounds (x-axis).

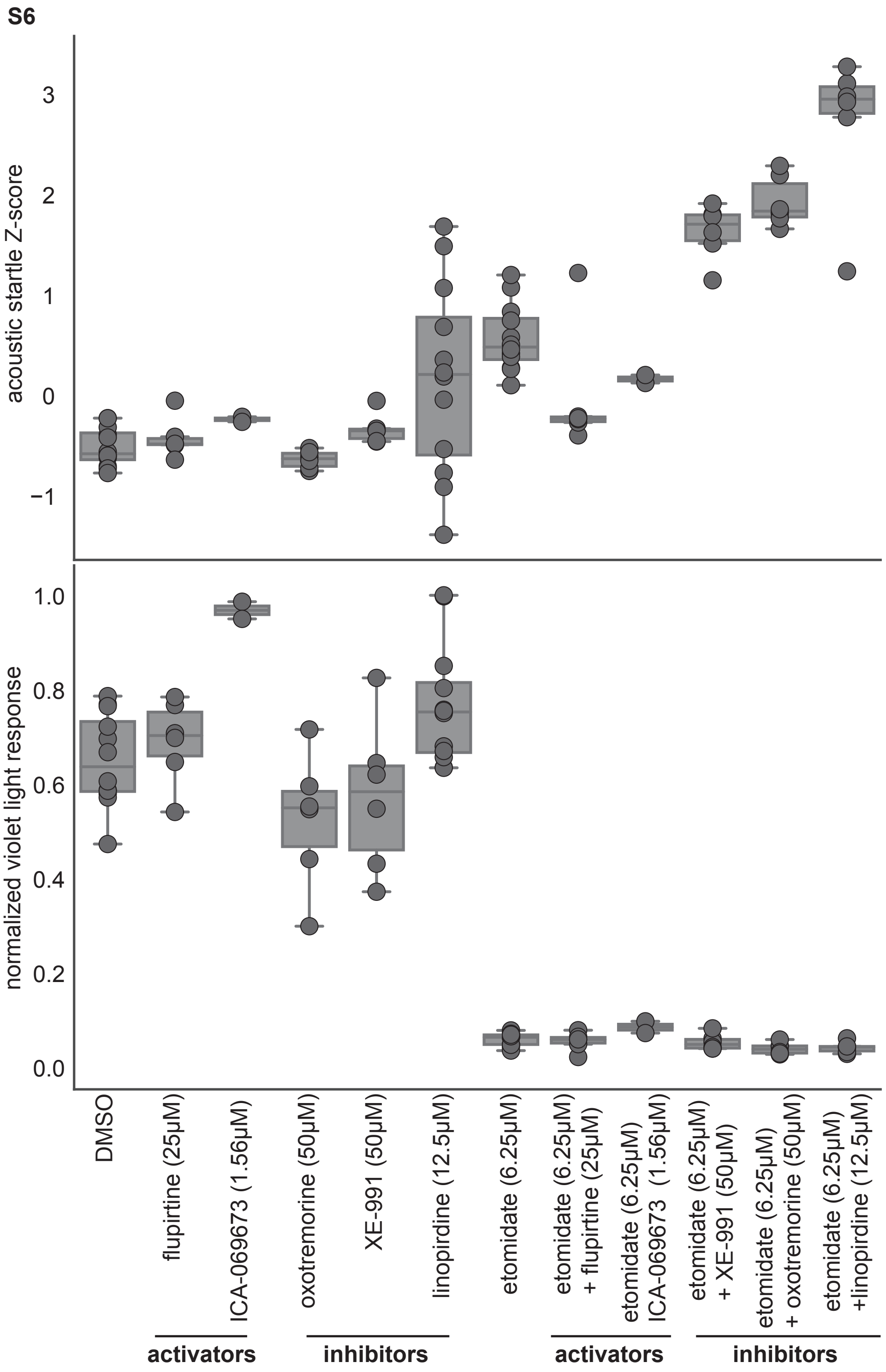


**Supplementary Figure 6. M-current ligands modify eASRs.** Boxplots depicting the motor activity (y-axis) of animals treated with the indicated compounds (x-axis) in response response to acoustic (top) or violet light stimuli (bottom). M-current activators and inhibitors were analyzed alone or combined with etomidate, at the indicated concentrations.

**S7**

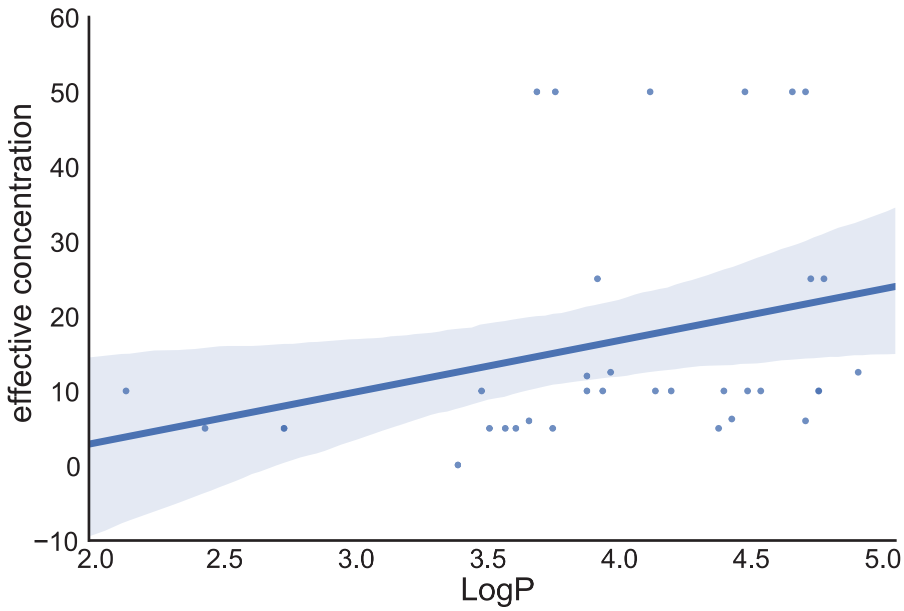


**Supplementary Figure 7. Hit compound efficacy does not correlate with hydrophobicity.** Animals were treated with hit compounds. For each compound, the cLogP (calculated partition coefficient) (x-axis) and minimum concentration required to cause the eASR phenotype, were plotted (y-axis). Unlike historical Myer-Overton analyses, the minimum effective concentration does not decrease with hydrophobicity. The best-fit line and shading represent the resulting regression line and a 95% confidence interval for that regression.

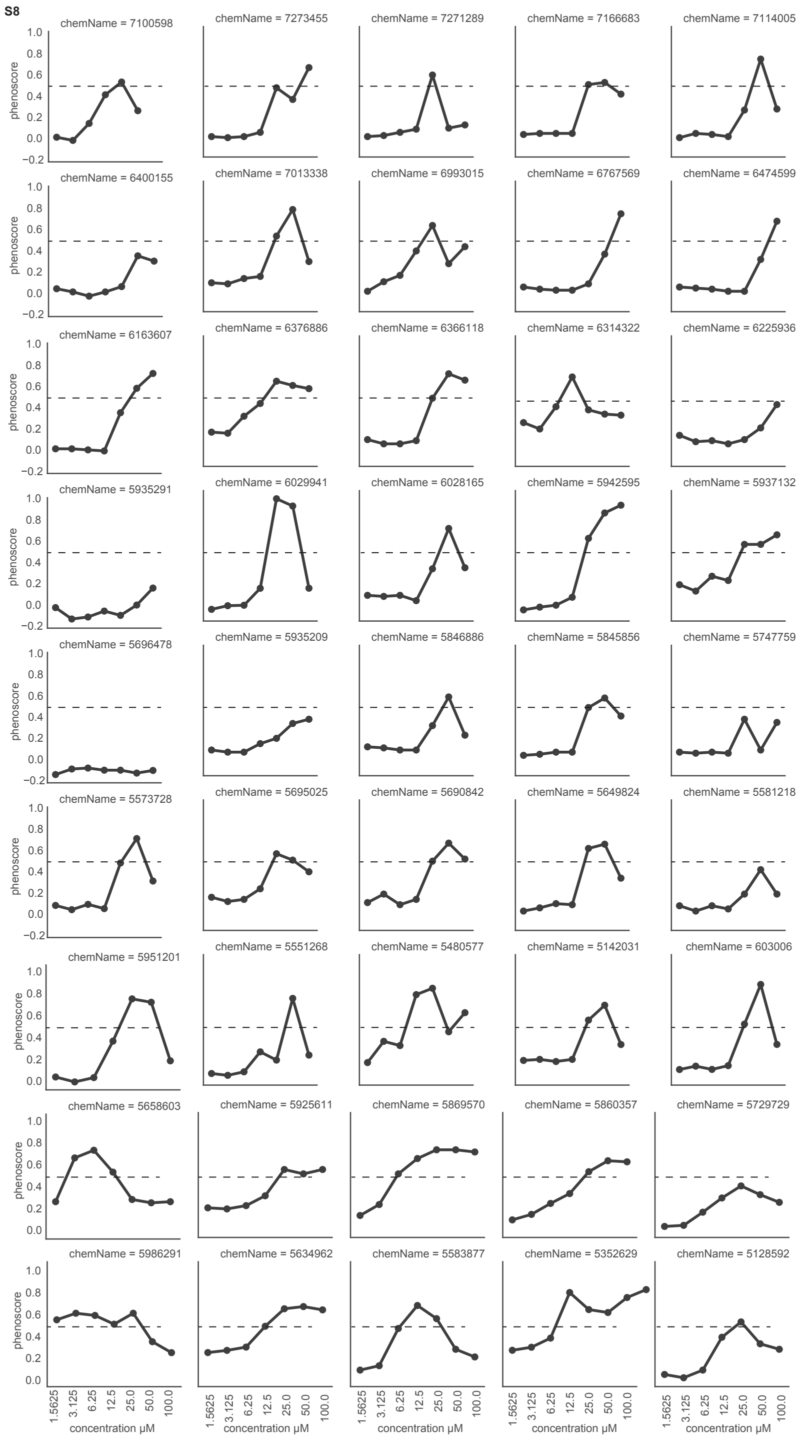


**Supplemental Figure 8. Dose response retest of primary hit compounds.** Average phenoscores (*y*-axis) of zebrafish treated with the indicated compounds (n = 12 wells) at the indicated concentrations (x-axis).

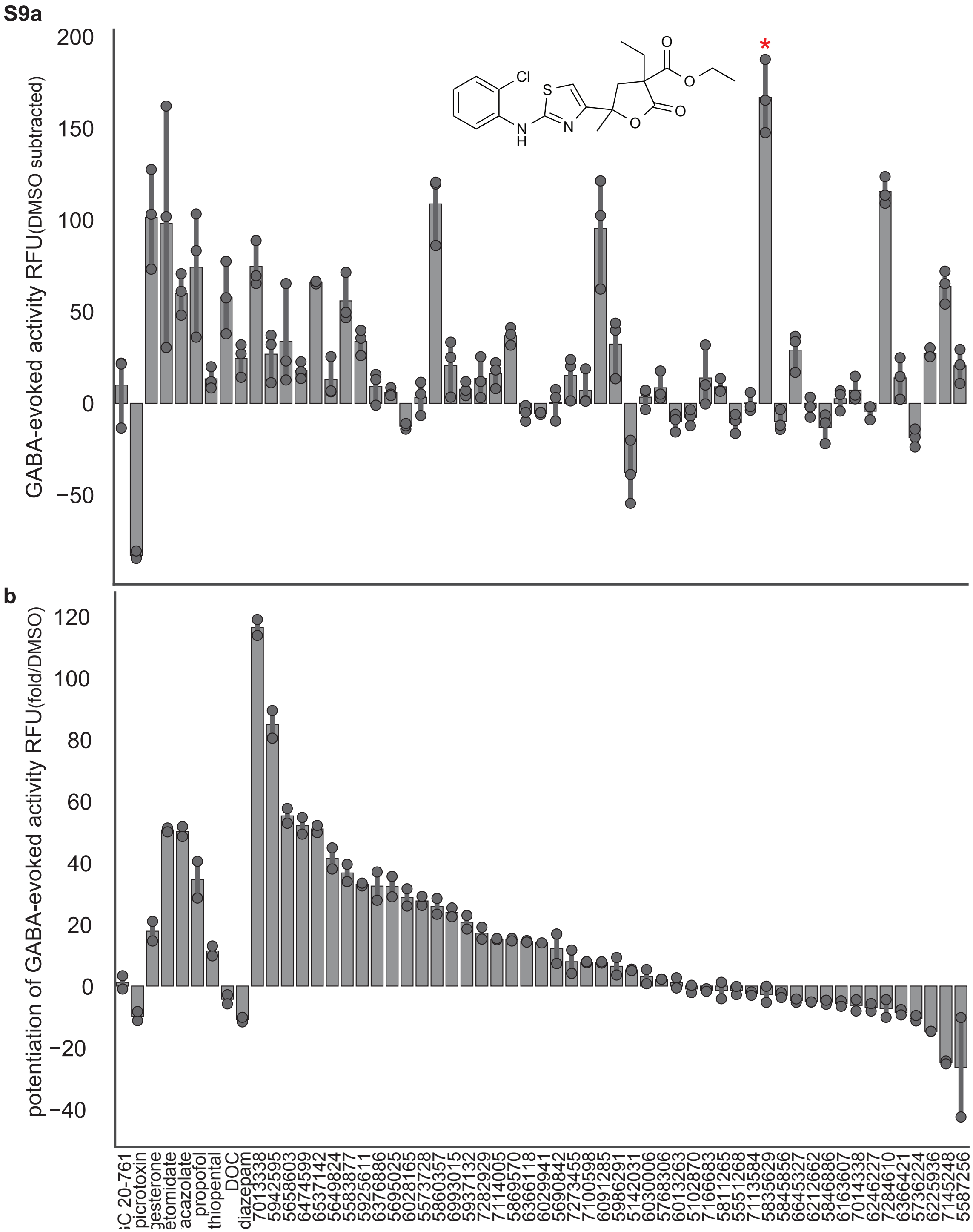


**Supplementary Figure 9. Hit compounds cause direct and indirect activation of GABARs. (a,b)** Human GABAAR activation (*y*-axis) was measured by FLIPR analysis in random fluorescent units (RFUs). Direct **(a)** and indirect **(b)** activation was analyzed for the indicated hit compounds (x-axis, n = 2-4).

**S10**

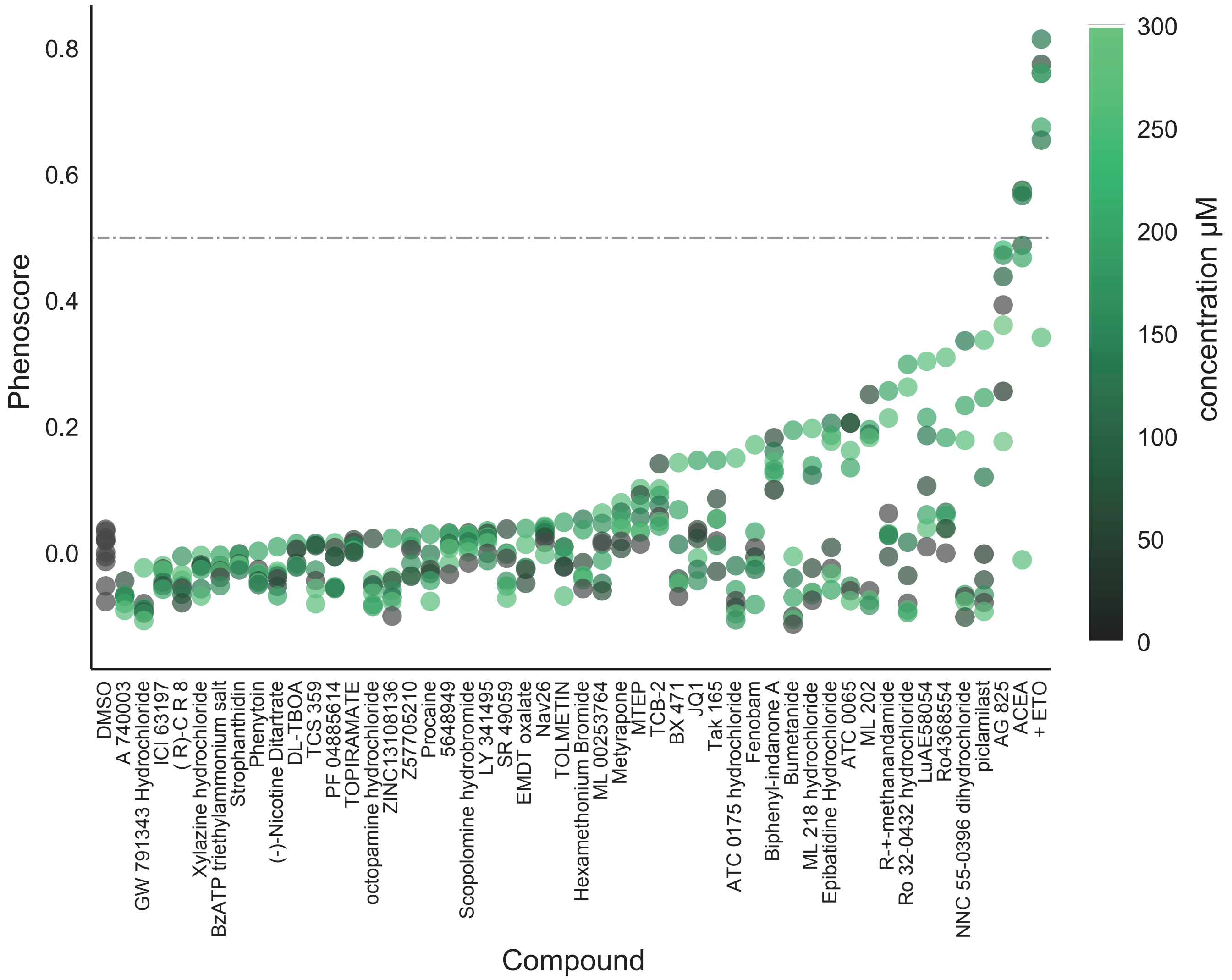


**Supplementary Figure 10. Phenoscores of ligands at targets with low value EFs.** Ligands for targets with low (left of dotted line) and high (right of dotted line) EF scores. The plot shows the phenoscore (y-axis) of the indicated compounds (x-axis). Color bar represents concentration in μM.

**S11**

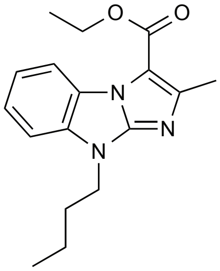

5658603

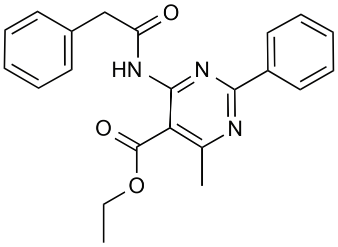

5951201

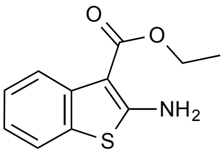

5142031

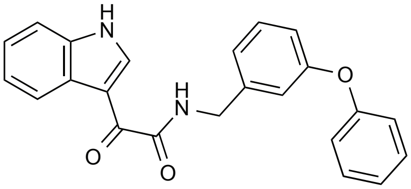

7145248

**Supplementary Figure 11. Chemical structures of hit compounds predicted to target GABAARs by SEA.**

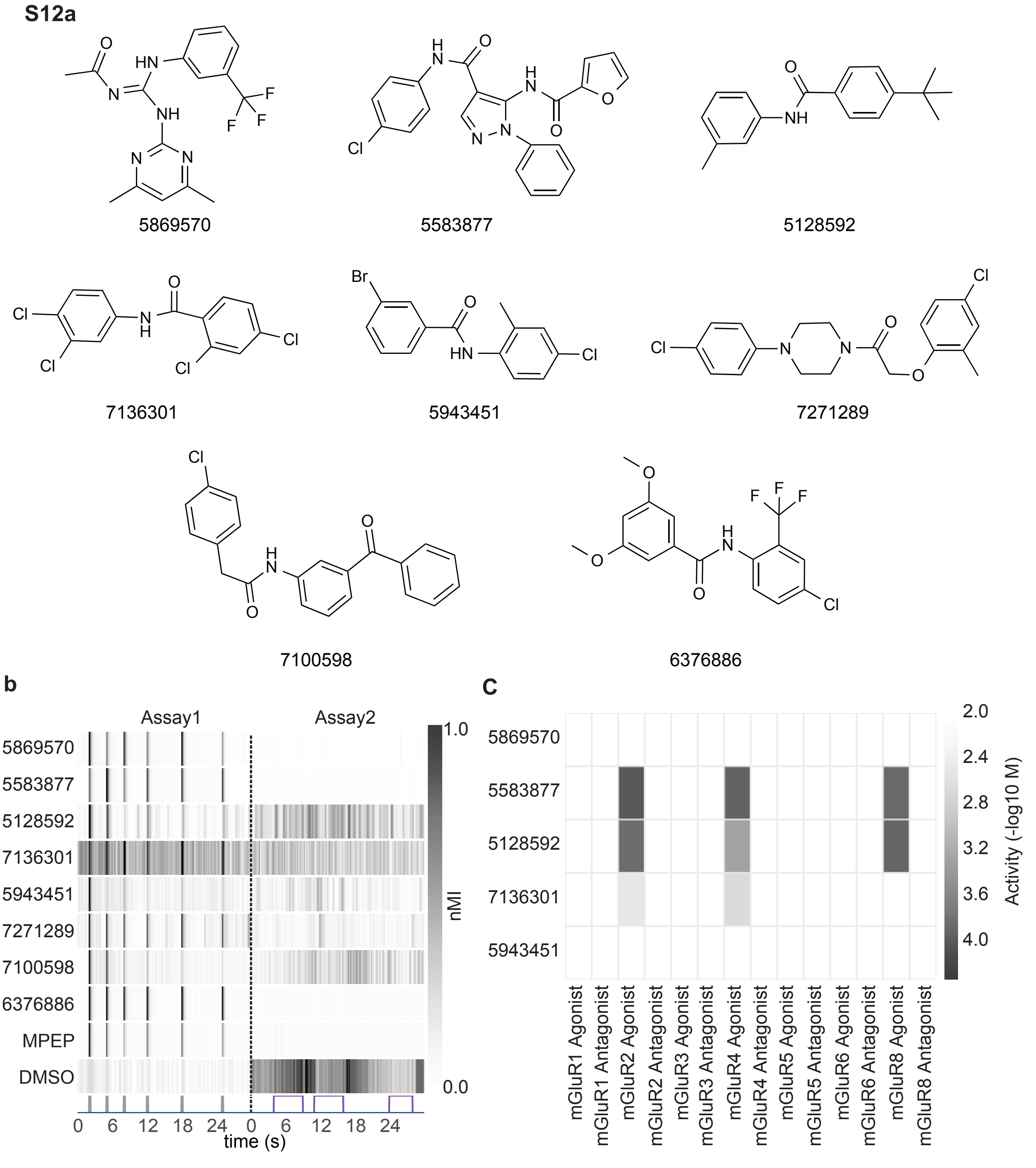


**Supplementary Figure 12. Characterization of hit compounds predicted to target mGluR by SEA. (a)** Chemical structures of 8 hit compounds predicted to target mGluR. **(b)** The heatmap represents the normalized motion index (nMI) of larvae treated with the indicated compounds. Assay 1 is composed of 6 low amplitude acoustic stimuli; Assay 2 is a series of 3 violet light pulses as indicated on the *x*-axis. MPEP is a known mGluR4/5 ligand. Compounds were tested for agonist and antagonist activity in Gq functional assays in-vitro. **(c)** The heat map represents the activity of 5 novel mGluR predicted compounds (y-axis) at the indicated receptor (x-axis). Low-level activation of mGlur2/4 was detected for compounds 5583877, 5128592, and 7136301 (46.13 μM to 2871 μM).

S13

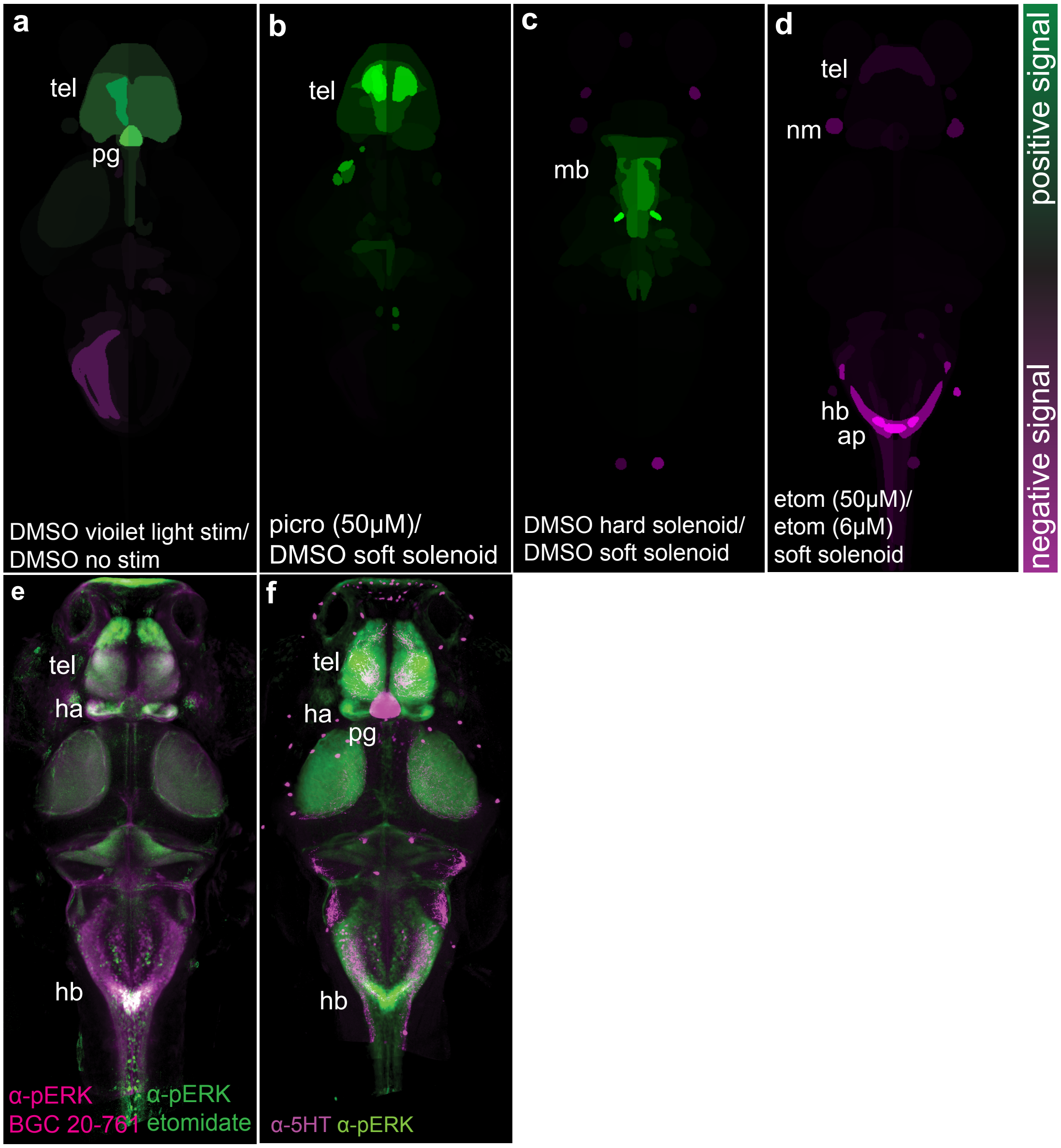


**Supplementary Figure 13. pERK whole brain neural activity maps in control assays and 5-HT immunohistochemistry.**

**(a-d)** Brain activity maps showing significant ΔpERK signals using the Z-brain online reference tool (n = 5-10 animals/condition). Heatmaps indicate positive (green), negative (purple), and nonsignificant (black) changes in pERK labeling ($p < 0.0005$, Mann-Whitney U test). All activity maps are comparisons between the indicated treatment conditions. (**e**) Overlay of  average α-pERK signal for BGC 20-761(magenta), and etomidate treated animals (green). (**f**) Overlay of α-5HT staining (magenta) and the average α-pERK staining (green) for BGC 20-761 treatment. Abbreviations: tel, telencephalon; mb, midbrain; ot, optic tectum; hb, hindbrain; ha, habenula;  ob, olfactory bulb; nm, neuromast; ap, area postrema; pg, pineal gland.

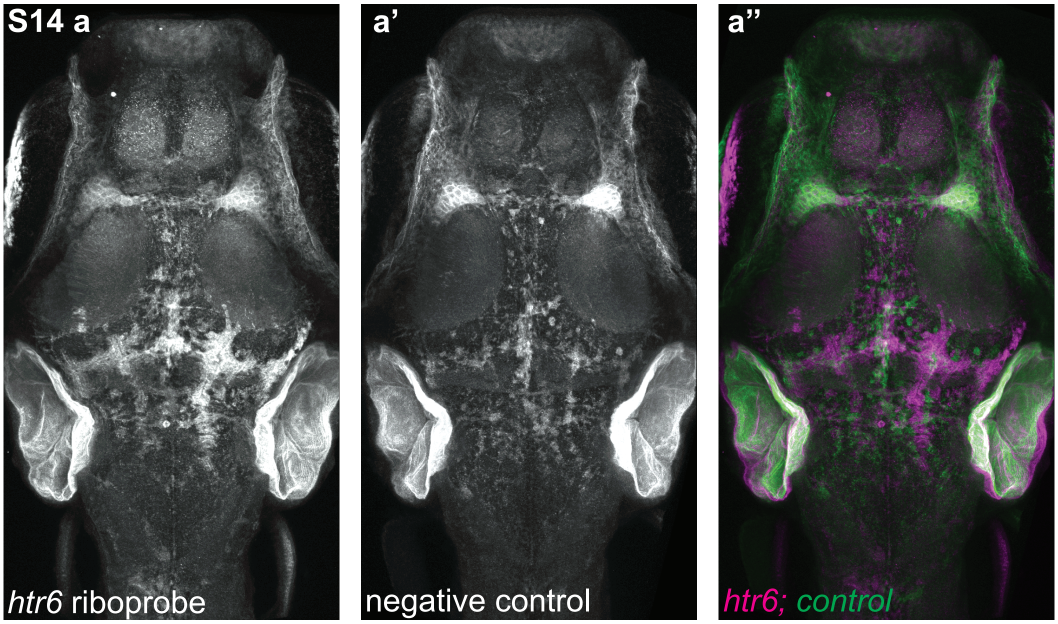


**Supplementary Figure 14. Fluorescent in situ hybridization of the zebrafish *htr6* transcript shows low expression in the telencephalon. (a-a’’)** Confocal projections from image registered animals showing transcripts for *htr6* (a) and non specific negative control antisense probe (a’), overlay in (a’’).

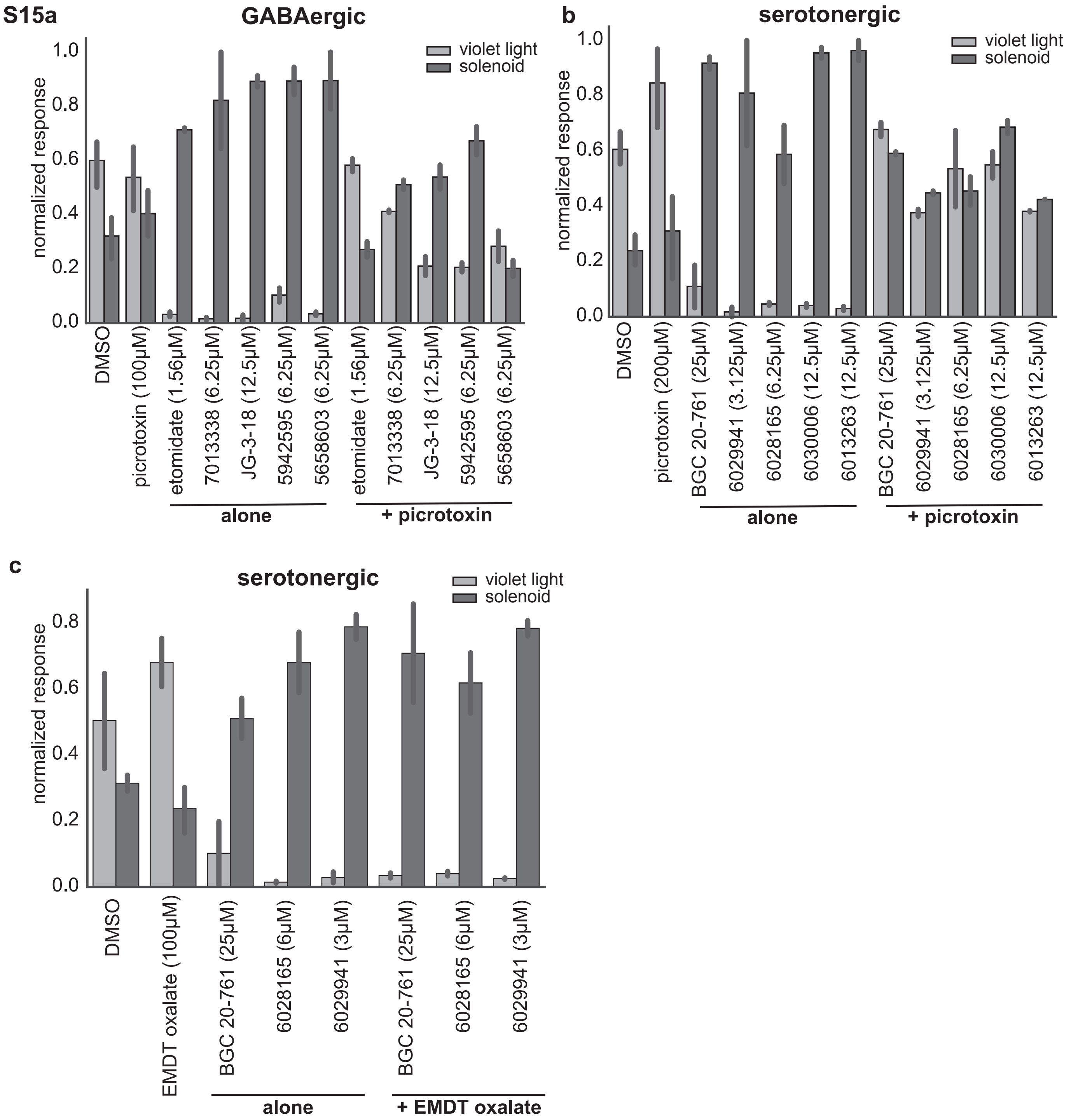


**Supplementary Figure 15. The GABAergic antagonist picrotoxin reverses the eASRs-induced by some ligands, but the serotonergic agonist EMDT oxolate does not. (a-c)** Normalized behavioral responses (y-axis) of animals treated with the indicated compounds (x-axis).

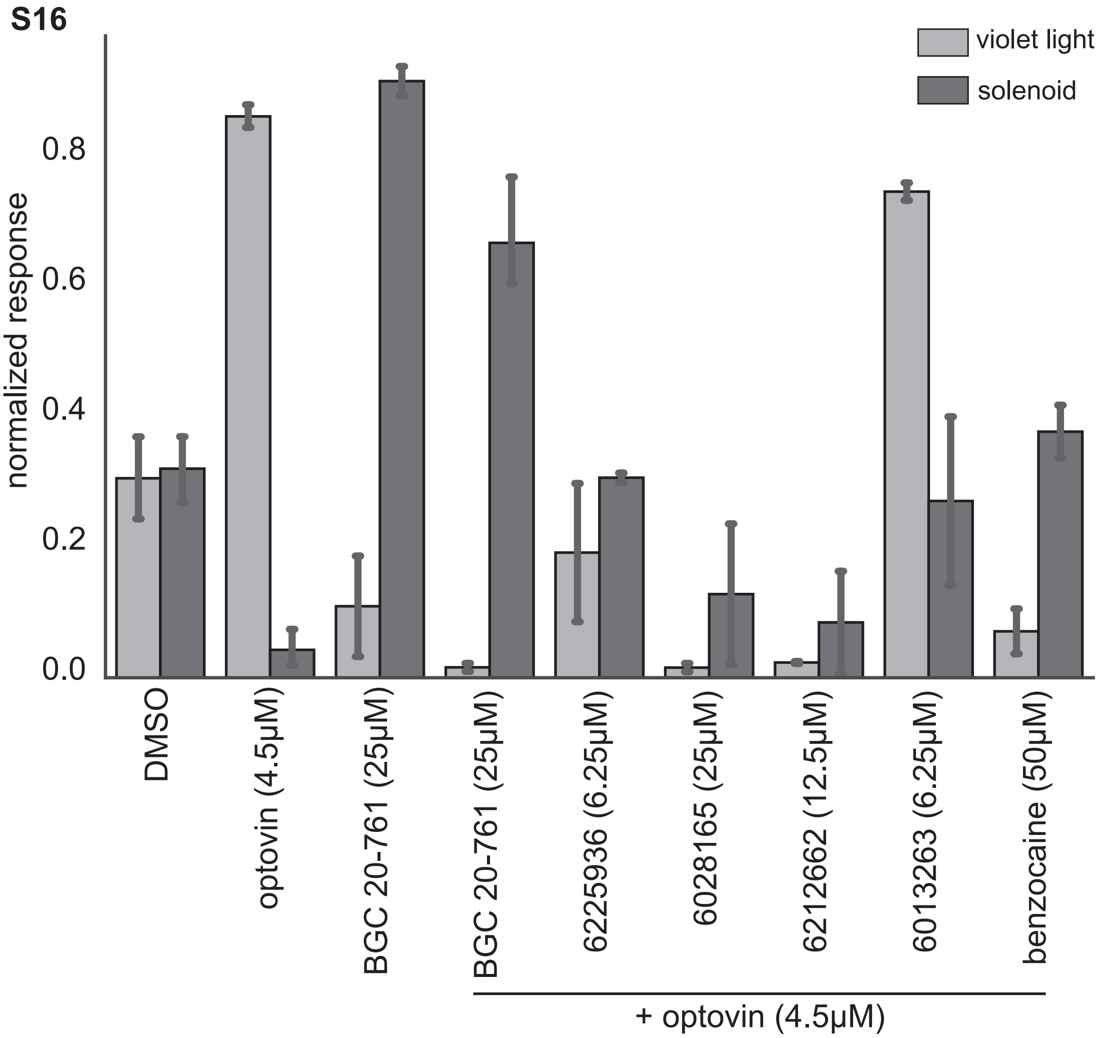


**Supplementary Figure 16. Serotonergic hit compounds inhibit optovin response.** Normalized behavioral response (y-axis) of animals treated with the indicated compounds (x-axis).

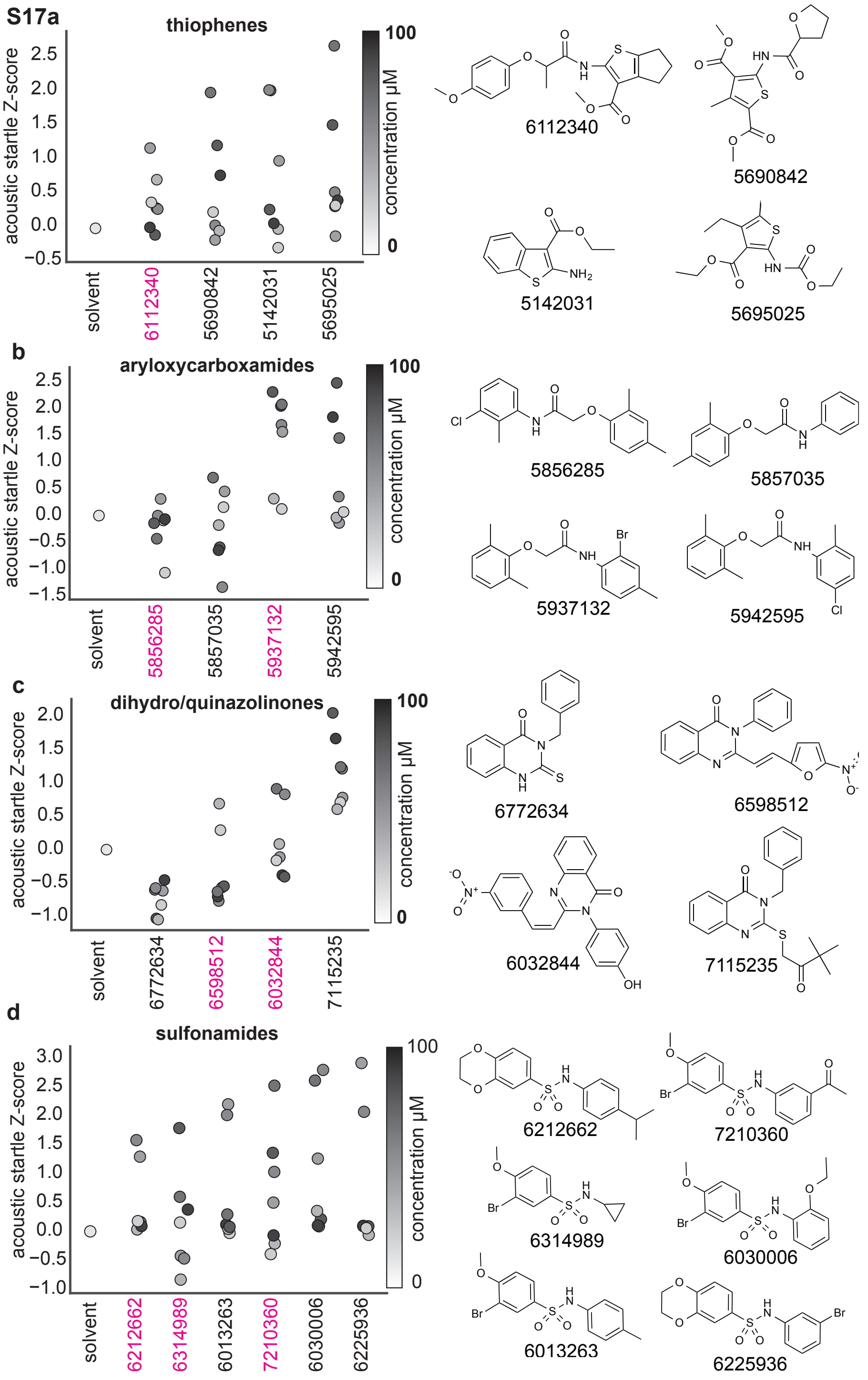


**Supplementary Figure 17. Preliminary SAR of key compound classes. (a-d)** The plots show the Z-score of the acoustic startle response (y-axis) in animals treated with the indicated compounds (x-axis). The compound structures in each class are shown to the right of each plot including the original hit compounds (black) and their analogs (red). Many analogs did not cause the eASR behaviors.

**S18**

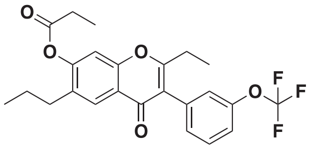

**JG-13**

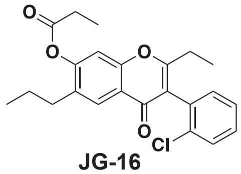

**JG-16**

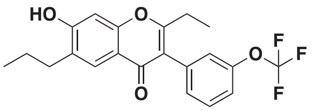

**JG-17**

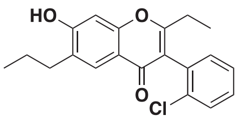

**JG-18**

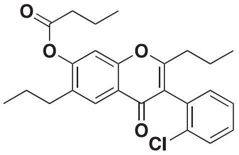

**JG-29**

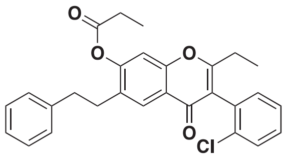

**JG-30**

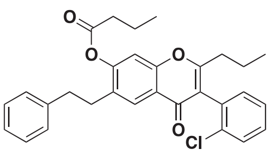

**JG-31**

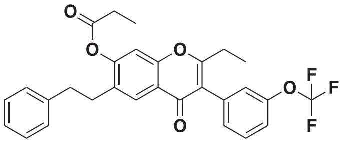

**JG-34**

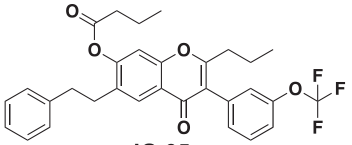

**JG-35**

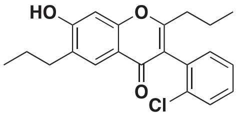

**JG-37**

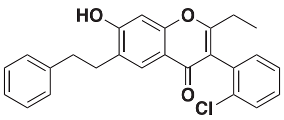

**JG-38**

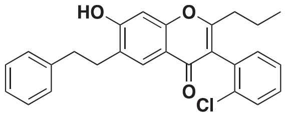

**JG-39**

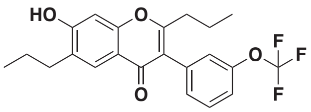

**JG-41**

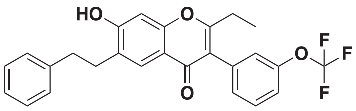

**JG-42**

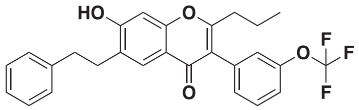

**JG-43**

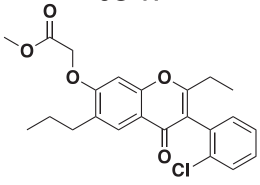

**JG-44**

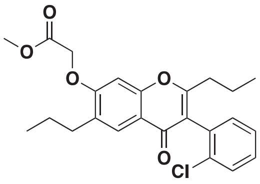

**JG-45**

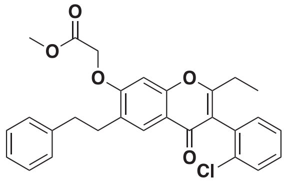

**JG-46**

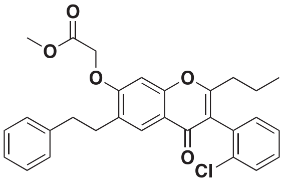

**JG-47**

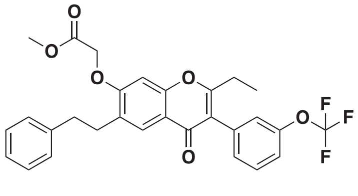

**JG-48**

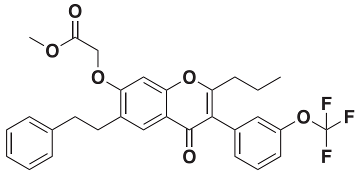

**JG-49**

**Supplementary Figure 18. Chemical structures of isoflavone analogs.**

**S19a**

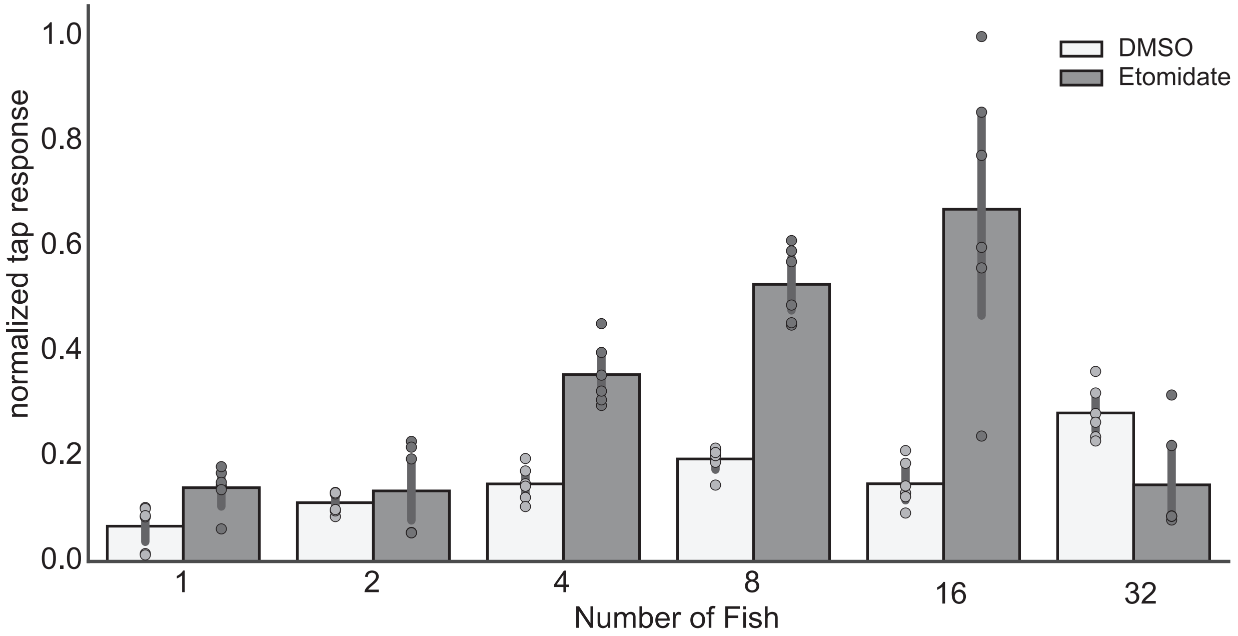


**b**

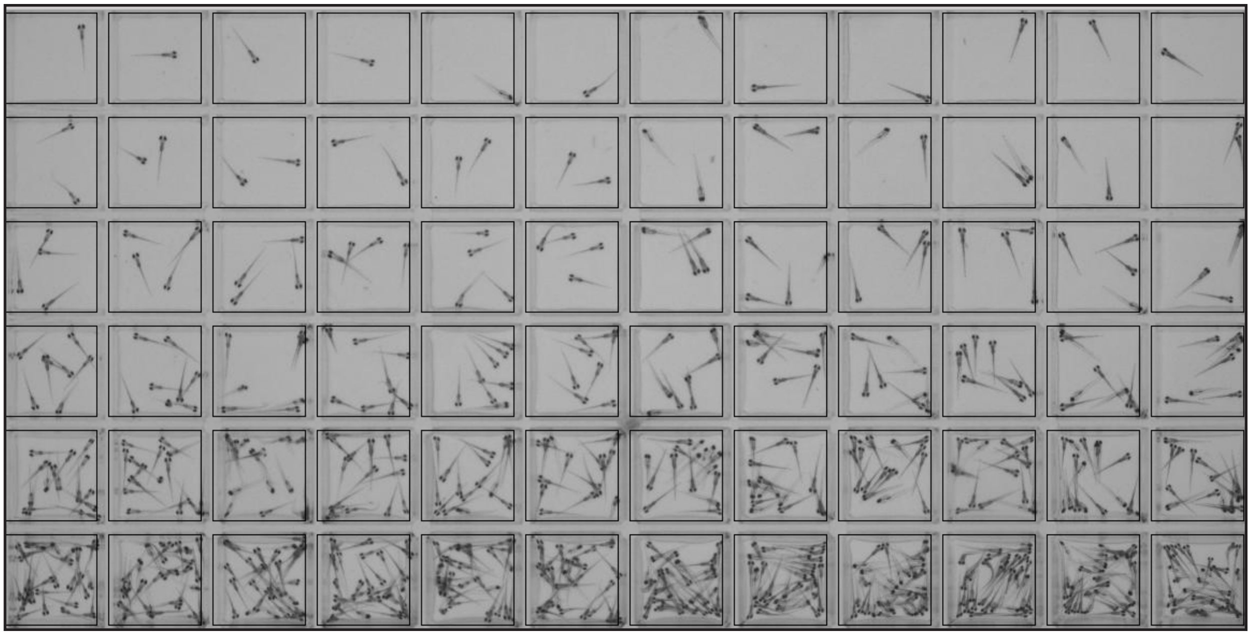

**Supplementary Figure 19. Group size affects eASR quantification.** To determine the impact of group size on this assay, we analyzed eASR behaviors from animals in different group sizes (1, 2, 4, 8, 12, 16, 32 animals per well). Groups of 8 and 16 animals generated the most robust MI values. Here, we chose to use 8 animals per group because it balanced a small group size with high signal to noise using the MI metric. **(a)** Bar graph illustrating the average tap response (*y*-axis) per tap stimulus (each marker represents one of 6 total stimuli averaged over 6 replicate wells), for wells with the indicated number of fish larvae (*x*-axis) and treated with DMSO control or 6μM etomidate, as indicated. **(b)** Representative image of wells containing increasing numbers of animals.

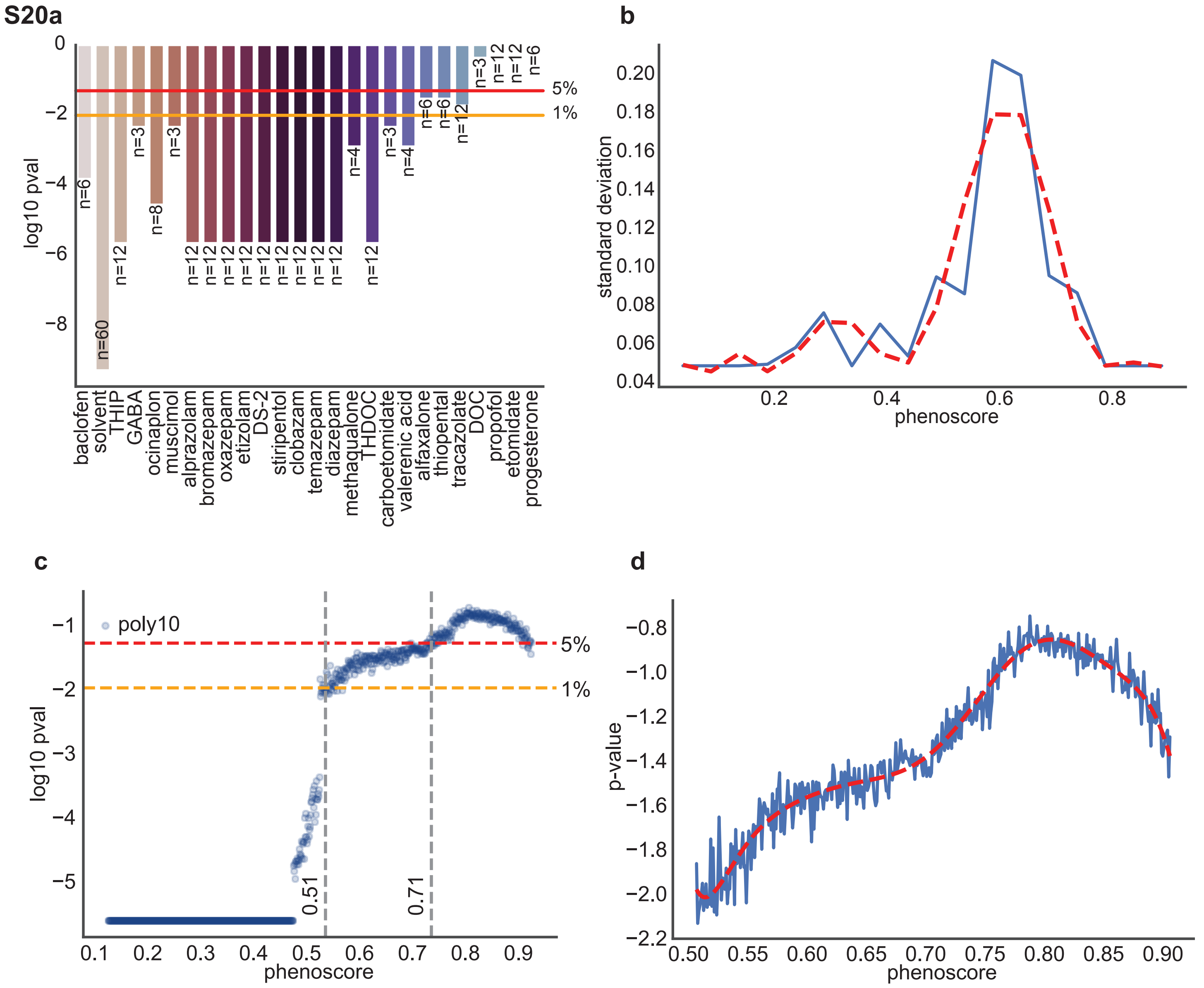


**Supplementary Figure 20. Statistical analysis of phenotypic thresholds for $GABA_A$R ligands.** **(a)** This plot shows the Kolmogorov-Smirnov (KS Test) statistic for the highest-scoring profiles produced by the indicated treatments *(y*-axis). On the x-axis, the ligands are sorted in order of ascending average phenoscore (left to right) from lowest to highest. Horizontal lines on the *y*-axis indicate the 1%( yellow) and 5% (red) *P* value significance thresholds **(b)** Plot showing the standard deviations for GABAAR ligands as a function of phenoscore with a 10th order polynomial. **(c)** Plot showing simulated *P* values as a function of phenoscore. Horizontal dashed lines indicate 1% and 5% P value thresholds, and vertical lines indicate the phenoscores at which these thresholds are met (0.51 and 0.71, respectively). **(d)** Plot showing a 10th order polynomial fit for the smooth region of the simulation where phenoscore > 0.5 in panel (c).

## REFERENCES

1. Lounkine, E. *et al.* Large-scale prediction and testing of drug activity on side-effect targets. *Nature* **486**, 361–367 (2012).
2. Bruni, G. *et al.* Zebrafish behavioral profiling identifies multitarget antipsychotic-like compounds. *Nat. Chem. Biol.* **12**, 559–566 (2016).
3. Gaspar, P. & Lillesaar, C. Probing the diversity of serotonin neurons. *Philos. Trans. R. Soc. Lond. B Biol. Sci.* **367**, 2382–2394 (2012).
4. Gross-Thebing, T., Paksa, A. & Raz, E. Simultaneous high-resolution detection of multiple transcripts combined with localization of proteins in whole-mount embryos. *BMC Biol.* **12**, 55 (2014).
5. Barnard, E. A. *et al.* International Union of Pharmacology. XV. Subtypes of gamma-aminobutyric acidA receptors: classification on the basis of subunit structure and receptor function. *Pharmacol. Rev.* **50**, 291–313 (1998).
6. Reis, G. M. L. & Duarte, I. D. G. Baclofen, an agonist at peripheral GABAB receptors, induces antinociception via activation of TEA-sensitive potassium channels. *Br. J. Pharmacol.* **149**, 733–739 (2006).
7. Masiulis, S. *et al.* GABAA receptor signalling mechanisms revealed by structural pharmacology. *Nature* **565**, 454–459 (2019).
8. Johnston, G. A. R. Muscimol as an ionotropic GABA receptor agonist. *Neurochem. Res.* **39**, 1942–1947 (2014).
9. Jensen, M. L. *et al.* A study of subunit selectivity, mechanism and site of action of the delta selective compound 2 (DS2) at human recombinant and rodent native GABA(A) receptors. *Br. J. Pharmacol.* **168**, 1118–1132 (2013).
10. Braestrup, C. & Squires, R. F. Pharmacological characterization of benzodiazepine receptors in the brain. *Eur. J. Pharmacol.* **48**, 263–270 (1978).
11. Pernea, M. & Sutcliffe, A. G. Clobazam and Its Use in Epilepsy. *Pediatr. Rep.* **8**, 6516 (2016).

12. Heel, R. C., Brogden, R. N., Speight, T. M. & Avery, G. S. Temazepam: a review of its pharmacological properties and therapeutic efficacy as an hypnotic. *Drugs* **21**, 321–340 (1981).

13. Lippa, A. *et al.* Selective anxiolysis produced by ocinaplon, a GABA(A) receptor modulator. *Proc. Natl. Acad. Sci. U. S. A.* **102**, 7380–7385 (2005).

14. Sanna, E. *et al.* Molecular and neurochemical evaluation of the effects of etizolam on GABAA receptors under normal and stress conditions. *Arzneimittelforschung* **49**, 88–95 (1999).

15. Thompson, S.-A., Wingrove, P. B., Connelly, L., Whiting, P. J. & Wafford, K. A. Tracazolate reveals a novel type of allosteric interaction with recombinant gamma-aminobutyric acid(A) receptors. *Mol. Pharmacol.* **61**, 861–869 (2002).

16. Fisher, J. L. The effects of stiripentol on GABA(A) receptors. *Epilepsia* **52 Suppl 2**, 76–78 (2011).

17. Hammer, H. *et al.* A Multifaceted GABAA Receptor Modulator: Functional Properties and Mechanism of Action of the Sedative-Hypnotic and Recreational Drug Methaqualone (Quaalude). *Mol. Pharmacol.* **88**, 401–420 (2015).

18. Luger, D. *et al.* Identification of the putative binding pocket of valerenic acid on GABAA receptors using docking studies and site-directed mutagenesis. *Br. J. Pharmacol.* **172**, 5403–5413 (2015).

19. Hosie, A. M., Wilkins, M. E. & Smart, T. G. Neurosteroid binding sites on GABA(A) receptors. *Pharmacol. Ther.* **116**, 7–19 (2007).

20. Garcia, P. S., Kolesky, S. E. & Jenkins, A. General anesthetic actions on GABA(A) receptors. *Curr. Neuropharmacol.* **8**, 2–9 (2010).

21. Troupin, A. S., Green, J. R. & Halpern, L. M. Carbamazepine (Tegretol) as an anticonvulsant. A controlled double-blind comparison with diphenylhydantoin (Dilantin). *Acta Neurol. Scand. Suppl.* **60**, 13–26 (1975).

22. Yaari, Y., Selzer, M. E. & Pincus, J. H. Phenytoin: mechanisms of its anticonvulsant action. *Ann. Neurol.* **20**, 171–184 (1986).

23. Wong, D. T., Bymaster, F. P. & Engleman, E. A. Prozac (fluoxetine, Lilly 110140), the first selective serotonin uptake inhibitor and an antidepressant drug: twenty years since its first

publication. *Life Sci.* **57**, 411–441 (1995).

24. Stahl, S. M. Mechanism of action of trazodone: a multifunctional drug. *CNS Spectr.* **14**, 536–546 (2009).

25. Simons, F. E. R. & Simons, K. J. Histamine and H1-antihistamines: celebrating a century of progress. *J. Allergy Clin. Immunol.* **128**, 1139–1150.e4 (2011).

26. Jann, M. W. Buspirone: an update on a unique anxiolytic agent. *Pharmacotherapy* **8**, 100–116 (1988).

27. Maletzky, B. M. Anxiolytic efficacy of alprazolam compared to diazepam and placebo. *J. Int. Med. Res.* **8**, 139–143 (1980).

28. Calcaterra, N. E. & Barrow, J. C. Classics in chemical neuroscience: diazepam (valium). *ACS Chem. Neurosci.* **5**, 253–260 (2014).

29. Janecek, J., Vestre, N. D., Schiele, B. C. & Zimmermann, R. Oxazepam in the treatment of anxiety states: a controlled study. *J. Psychiatr. Res.* **4**, 199–206 (1966).

30. Misra, L. K., Erpenbach, J. E., Hamlyn, H. & Fuller, W. C. Quetiapine: a new atypical antipsychotic. *S. D. J. Med.* **51**, 189–193 (1998).

31. Bymaster, F. P. *et al.* Radioreceptor binding profile of the atypical antipsychotic olanzapine. *Neuropsychopharmacology* **14**, 87–96 (1996).

32. Waal-Manning, H. J. Atenolol and three nonselective beta-blockers in hypertension. *Clin. Pharmacol. Ther.* **25**, 8–18 (1979).

33. Stapleton, M. P. Sir James Black and propranolol. The role of the basic sciences in the history of cardiovascular pharmacology. *Tex. Heart Inst. J.* **24**, 336–342 (1997).

34. Hillard, C. J. *et al.* Synthesis and characterization of potent and selective agonists of the neuronal cannabinoid receptor (CB1). *J. Pharmacol. Exp. Ther.* **289**, 1427–1433 (1999).

35. Abadji, V. *et al.* (R)-methanandamide: a chiral novel anandamide possessing higher potency and metabolic stability. *J. Med. Chem.* **37**, 1889–1893 (1994).

36. Sanger, D. J. & Depoortere, H. The Pharmacology and Mechanism of Action of Zolpidem. *CNS*

*Drug Rev.* **4**, 323–340 (1998).

37. Terrell, R. C. The invention and development of enflurane, isoflurane, sevoflurane, and desflurane. *Anesthesiology* **108**, 531–533 (2008).
38. Chidambaran, V., Costandi, A. & D'Mello, A. Propofol: a review of its role in pediatric anesthesia and sedation. *CNS Drugs* **29**, 543–563 (2015).
39. Forman, S. A. Clinical and molecular pharmacology of etomidate. *Anesthesiology* **114**, 695–707 (2011).
40. Kurdi, M. S., Theerth, K. A. & Deva, R. S. Ketamine: Current applications in anesthesia, pain, and critical care. *Anesth Essays Res* **8**, 283–290 (2014).
41. Fozzard, H. A., Sheets, M. F. & Hanck, D. A. The sodium channel as a target for local anesthetic drugs. *Front. Pharmacol.* **2**, 68 (2011).
42. Clarkson, C. W. & Hondeghem, L. M. Mechanism for bupivacaine depression of cardiac conduction: fast block of sodium channels during the action potential with slow recovery from block during diastole. *Anesthesiology* **62**, 396–405 (1985).
43. Carter, K. M., Woodley, C. M. & Brown, R. S. A review of tricaine methanesulfonate for anesthesia of fish. *Rev. Fish Biol. Fish.* **21**, 51–59 (2011).
44. Outland, T. & Hanlon, C. R. THE USE OF PROCAINE HYDROCHLORIDE AS A THERAPEUTIC AGENT. *JAMA* **114**, 1330–1333 (1940).

**Supplementary Table 1. CNS depressants characterized on zebrafish larval behavior.**

| class ID | chemical name | class | citation |
|---|---|---|---|
| 1 | carbamazepine | anticonvulsant | 19 |
| 1 | phenytoin | anticonvulsant | 20 |
| 2 | fluoxetine | antidepressant | 21 |
| 2 | trazodone | antidepressant | 22 |
| 3 | diphenhydramine | antihistamine | 23 |
| 3 | dimenhydrinate | antihistamine | 23 |
| 3 | promethazine | antihistamine | 23 |
| 4 | buspirone | anxiolytic | 24 |
| 4 | alprazolam | anxiolytic | 25 |
| 4 | diazepam | anxiolytic | 26 |
| 4 | oxazepam | anxiolytic | 27 |
| 5 | quetiapine | atypical antipsychotic | 28 |
| 5 | olanzapine | atypical antipsychotic | 29 |
| 6 | atenolol | beta blocker | 30 |
| 6 | propranolol | beta blocker | 31 |
| 7 | ACPA | cannabinoid | 32 |
| 7 | methanandamide | cannabinoid | 33 |
| 8 | zolpidem | hypnotic | 34 |
| 9 | benzocaine | local anesthetic | 35 |
| 9 | lidocaine | local anesthetic | 39 |
| 9 | bupivacaine | local anesthetic | 40 |
| 9 | tricaine | local anesthetic | 41 |
| 9 | procaine | local anesthetic | 42 |
| 10 | ketamine | intravenous anesthetic | 38 |
| 10 | isoflurane | inhalational anesthetic | 35 |
| 10 | propofol | intravenous anesthetic | 36 |
| 10 | etomidate | intravenous anesthetic | 37 |

**Supplemental Table 2. Viability of anesthetic treated animals**

| treatment | concentration | alive/total 10 min | alive/total 1 hour | alive/total 5 hours |
|---|---|---|---|---|
| DMSO | 0 µM | 100/100 | 100/100 | 100/100 |
| etomidate | 3 µM | 100/100 | 100/100 | 100/100 |
| etomidate | 6 µM | 100/100 | 100/100 | 100/100 |
| etomidate | 12 µM | 100/100 | 100/100 | 98/100 |
| propofol | 3 µM | 100/100 | 100/100 | 100/100 |
| propofol | 6 µM | 100/100 | 100/100 | 100/100 |
| propofol | 12 µM | 100/100 | 100/100 | 99/100 |

**Supplementary Table 3. GABAAR ligand reference set**

| Chemical Name | Class | Reference |
|---|---|---|
| dmso | vehicle control | NA |
| baclofen | $GABA_B$ agonist | 4 |
| GABA | orthosteric $GABA_A$R agonist | 5 |
| muscimol | orthosteric $GABA_A$R agonist | 6 |
| gaboxadol (THIP) | $GABA_A$R delta subtype preferring PAM | 7 |
| DS-2 | $GABA_A$R delta subtype preferring PAM | 7 |
| ocinaplon | $GABA_A$R BZ-site PAM | 11 |
| bromazepam | $GABA_A$R BZ-site PAM | 8 |
| etizolam | $GABA_A$R BZ-site PAM | 12 |
| alprazolam | $GABA_A$R BZ-site PAM | 5 |
| oxazepam | $GABA_A$R BZ-site PAM | 8 |
| clobazam | $GABA_A$R BZ-site PAM | 9 |
| temazepam | $GABA_A$R BZ-site PAM | 10 |
| diazepam | $GABA_A$R BZ-site PAM | 5 |
| stripentol | $GABA_A$R non BZ-site PAM | 14 |
| methaqualone | $GABA_A$R non BZ-site PAM | 15 |
| valerinic acid | $GABA_A$R non BZ-site PAM | 16 |
| thiopental | $GABA_A$R non BZ-site PAM | 18 |
| tracazolate | $GABA_A$R non BZ-site PAM | 13 |
| carboetomidate | $GABA_A$R anesthetic PAM | 18 |
| propofol | $GABA_A$R anesthetic PAM | 18 |
| etomidate | $GABA_A$R anesthetic PAM | 18 |
| tetrahydrodeoxycorticosterone (THDOC) | $GABA_A$R neurosteroid PAM | 17 |
| alphaxalone | $GABA_A$R neurosteroid PAM | 17 |
| progesterone | $GABA_A$R neurosteroid PAM | 17 |
| deoxycorticosterone (DOC) | $GABA_A$R neurosteroid PAM | 17 |

**Supplementary Table 4. Chemical names and SMILES of the top 125 hit compounds from a zebrafish behavioral drug screen.**

| Chemical Name | SMILES | Retested |
|---|---|---|
| progesterone | CC(=O)C1CCC2C3CCC4=CC(=O)CCC4(C)C3CCC12C | yes |
| alfaxalone | CC(=O)C1CCC2C3CCC4CC(O)CCC4(C)C3C(=O)CC12C | yes |
| DOC | CC12CCC(=O)C=C1CCC1C2CCC2(C)C(C(=O)CO)CCC12 | yes |
| 7166683 | Cc1cc(C)n2c(SCc3ccc(C(=O)c4ccccc4)cc3)nnc2n1 | yes |
| etomidate | CCOC(=O)c1cncn1C(C)c1ccccc1 | yes |
| 6587027 | CC(=O)c1cccc(NC(=O)c2ccc(-c3ccc(Cl)cc3)o2)c1 | no |
| etomidate | CCOC(=O)c1cncn1C(C)c1ccccc1 | yes |
| 6858658 | O=C(c1cc(Cl)ccc1Cl)N1CCN(c2cccc(C(F)(F)F)c2)CC1 | no |
| 5846886 | Nc1ccc(Oc2ccc(Cl)c3cccnc23)c(Cl)c1 | yes |
| 6767569 | Cc1nc2ccccc2n1C(=O)N(c1ccccc1)c1ccccc1 | yes |
| 6762995 | Fc1ccccc1OCCn1c(S)nc2ccccc21 | yes |
| alfaxalone | CC(=O)C1CCC2C3CCC4CC(O)CCC4(C)C3C(=O)CC12C | yes |
| 7013338 | CCc1cc2c(=O)c(-c3ccccc3Cl)c(C)oc2cc1OC | yes |
| 7010474 | CCc1cc2c(=O)c(-c3ccccc3Cl)coc2cc1OC | no |
| 6376886 | COc1cc(OC)cc(C(O)=Nc2ccc(Cl)cc2C(F)(F)F)c1 | yes |
| 7100598 | O=C(c1ccccc1)c1cccc(N=C(O)Cc2ccc(Cl)cc2)c1 | yes |
| 7113584 | Nc1ccc(OC(F)(F)F)cc1C(=O)c1ccccc1 | yes |
| 7114005 | CCOC(=O)c1c(C)n(Cc2ccco2)c2ccc(OC)cc12 | yes |
| 6576466 | O=[N+]([O-])c1cccc(C(O)=Nc2ccc(Cl)cc2C(F)(F)F)c1 | no |
| 6029941 | COc1ccc(OC)c(NS(=O)(=O)c2ccc(OC)c(Br)c2)c1 | yes |
| 7285168 | OC(=Nc1ccc(Cl)cc1F)c1cccc2ccccc12 | no |
| 7136301 | OC(=Nc1ccc(Cl)c(Cl)c1)c1ccc(Cl)cc1Cl | yes |
| 6890102 | CCOc1ccc2c(=O)c(-c3ccccc3Cl)c(C)oc2c1 | no |
| 6225936 | O=S(=O)(Nc1cccc(Br)c1)c1ccc2c(c1)OCCO2 | no |
| 6525784 | O=c1c2ccccc2nc(C=Cc2ccc([N+](=O)[O-])cc2)n1-c1ccccc1Cl | no |
| 7273455 | Cc1cccc(OCCn2c(NC(=O)c3ccco3)nc3ccccc32)c1 | yes |
| 6314322 | CC(C)(C)N=C(O)COc1ccc(C(C)(C)c2ccccc2)cc1 | yes |
| 6474599 | CCCN(CCC)S(=O)(=O)c1ccc(Cl)c(Cl)c1OC | yes |
| 6682129 | C=CCn1c(SCC(=O)c2cccs2)nc2sc(CC)cc2c1=O | no |
| ivermectin | CCC(C)C1OC2(CCC1C)CC1CC(CC=C(C)C(OC3CC(OC)C(OC4CC(OC)C(O)C(C)O4)C(C)O3)C(C)C=CC=C3COC4C(O)C(C)=CC(C(=O)O1C34O)O2 | yes |
| 6028165 | COc1ccc(S(=O)(=O)NC2CCCC2)cc1Br | yes |
| 7271289 | Cc1cc(Cl)ccc1OCC(=O)N1CCN(c2ccc(Cl)cc2)CC1 | yes |
| 5584178 | CCc1nc2c(cnn2-c2ccccc2)c(=O)n1-c1ccc(C)cc1 | no |
| 5735460 | COc1ccccc1CC(O)=Nc1ccc(Br)cc1F | no |
| 7305598 | OC(=Nc1cccc(N=C(O)c2cc(Cl)ccc2Cl)c1)c1cc(Cl)ccc1Cl | no |
| 6993015 | CC(C)CC(O)=NC(S)=NCc1ccccc1 | yes |
| progesterone | CC(=O)C1CCC2C3CCC4=CC(=O)CCC4(C)C3CCC12C | yes |
| 6645327 | COc1ccccc1-c1coc2cc(OC(=O)c3cccs3)ccc2c1=O | yes |
| 6772634 | O=c1c2ccccc2nc(S)n1Cc1ccccc1 | no |
| 6366118 | COc1ccc(S(=O)(=O)N2CCC(C)CC2)cc1Br | yes |
| 7282929 | CCOC(=O)c1c(N=C(O)C2CCCC2)sc2c1CCCC2 | yes |
| 6193422 | O=S(=O)(Nc1cccc(Cl)c1)c1ccc2c(c1)OCCO2 | no |
| 5694163 | Cc1csc(N=C(O)C2c3ccccc3Oc3ccccc32)n1 | no |
| 5754452 | COC(=O)c1cccc(N=C(O)COc2ccc(C(C)(C)C)cc2)c1 | yes |
| 6565557 | CCCCOc1ccc(C(=O)NC(C)C2COc3ccccc3O2)cc1 | yes |
| 5696478 | Cc1c(C(=O)OC(C)C)sc(N=C(O)C2CCCCC2)c1C(=N)O | yes |

**Supplementary Table 4. Continued**

| Chemical Name | SMILES | Retested |
|---|---|---|
| 5587256 | O=C(Nc1c(C(=O)Nc2ccc(Cl)cc2)cnn1-c1ccccc1)c1ccccc1F | yes |
| 5551268 | Clc1ccc(CSc2nc3ccccc3[nH]2)cc1 | yes |
| 5149665 | Brc1ccc(CSc2nc3ccccc3[nH]2)cc1 | no |
| 5869570 | CC(O)=NC(=Nc1cccc(C(F)(F)F)c1)Nc1nc(C)cc(C)n1 | yes |
| 5729729 | O=C(CC(c1ccccc1)c1ccccc1)N1CCN(c2ccc(F)cc2)CC1 | yes |
| 6877352 | CC(c1ccccc1)n1c(S)nc2ccccc21 | no |
| 6595002 | CCc1ccc(O)c(N=C(S)N=C(O)c2ccccc2I)c1 | no |
| 5352629 | COc1ccccc1N1CCN(C(=O)c2ccc(C(=O)c3c(C)cc(C)cc3C)cc2)CC1 | yes |
| 7184284 | CCc1cccc(C)c1N=C(O)COc1ccc(Cl)cc1Cl | no |
| 7114335 | Nc1ccc(SC(F)(F)F)cc1C(=O)c1ccccc1 | yes |
| 7014338 | CCc1cc2c(=O)c(-c3ccccc3Cl)coc2cc1O | yes |
| DOC | CC12CCC(=O)C=C1CCC1C2CCC2(C)C(C(=O)CO)CCC12 | yes |
| 5142031 | CCOC(=O)c1c(N)sc2ccccc12 | yes |
| 5695025 | CCOC(=O)c1c(N=C(O)OCC)sc(C)c1CC | yes |
| 5935291 | Cc1ccc(N2C(=O)CC(N3CCN(c4cccc(Cl)c4)CC3)C2=O)cc1Cl | yes |
| 6625147 | CC1CC(OCC(O)CN2CCN(c3ccccc3F)CC2)CC(C)(C)C1.Cl.Cl | no |
| 6340625 | O=C(c1cc2ccccc2o1)N1CCCC(c2ccccc2)C1 | no |
| 6383686 | C=C(C)Cn1c(-c2ccc(OC)c(OC)c2)nc2ccccc21 | no |
| 5813444 | CCC(O)=Nc1nc(-c2ccccc2)nc(SC)c1C(C)=O | no |
| 6570890 | CCOc1ccc(NCc2ccccc2NS(=O)(=O)c2ccc(C)cc2)cc1 | no |
| 6400155 | O=C(c1cc2ccccc2o1)N1CCN(c2ccc(C(F)(F)F)cc2[N+](=O)[O-])CC1 | yes |
| 5951201 | CCOC(=O)c1c(C)nc(-c2ccccc2)nc1N=C(O)Cc1ccccc1 | yes |
| riluzole | N=c1[nH]c2ccc(OC(F)(F)F)cc2s1 | yes |
| 5583877 | O=C(Nc1ccc(Cl)cc1)c1cnn(-c2ccccc2)c1N=C(O)c1ccco1 | yes |
| 6642835 | Cc1c(O)ccc(C(=O)Cc2ccccc2)c1O | no |
| 5573728 | CC(=O)c1c(C)nc2n(Cc3ccccc3)c3ccccc3n12 | yes |
| 5658603 | CCCCn1c2ccccc2n2c(C(=O)OCC)c(C)nc12 | yes |
| 6013263 | COc1ccc(S(=O)(=O)Nc2ccc(C)cc2)cc1Br | yes |
| riluzole | N=c1[nH]c2ccc(OC(F)(F)F)cc2s1 | yes |
| 6652383 | CC(C)(C)C(=O)Oc1ccc2c(=O)c(-c3ccccc3Cl)coc2c1 | no |
| 6522346 | CC(=O)Oc1ccc(OC(C)=O)c(S(=O)(=O)c2ccc(Cl)c(Cl)c2)c1 | no |
| 6271180 | Cc1cccc(OCC(=O)N2CCN(c3ccc(C(F)(F)F)cc3[N+](=O)[O-])CC2)c1C | yes |
| 5480577 | CCOC(=O)c1cc2n(c1N=CN(C)C)-c1ccccc1C2=O | yes |
| 6204912 | CCC(C)c1ccc(NC(=O)CC(C)(C)C)cc1 | no |
| 6386892 | Cc1cc(C)c(N=C(O)Cc2cccs2)c(C)c1 | no |
| 5846693 | OC(=NCc1ccco1)c1cc2nc(-c3ccc(F)cc3)cc(C(F)(F)F)n2n1 | no |
| 6030006 | CCOc1ccccc1NS(=O)(=O)c1ccc(OC)c(Br)c1 | yes |
| 6756477 | COc1cccc(-n2nnnc2SCC(O)=Nc2c(C)cc(C)cc2C)c1 | no |
| 6353053 | CC(C)(c1ccccc1)c1ccc(OCC(O)=Nc2ccccc2C(=N)O)cc1 | no |
| 6667020 | CCCOC(O)=Nc1nc(-c2ccc(C)cc2)c(C)s1 | no |
| 5978667 | OC1=Nc2c(ccc3ccccc23)C(c2ccc(C(F)(F)F)cc2)C1 | no |
| 5649824 | CCC(=O)c1c(C)nc2n(Cc3ccccc3)c3ccccc3n12 | yes |
| 7211089 | O=[N+]([O-])c1cccc(N=C(O)c2ccc(Cl)cc2Cl)c1 | no |
| 5577990 | O=C(c1c(N=C(O)c2ccccc2Cl)sc2c1CCCCC2)N1CCCCC1 | no |
| 6805976 | CCC(C)c1ccccc1N=C(O)c1ccccc1I | no |
| 5799128 | CCCCOc1nnc(-c2ccccc2)c2ccccc12 | no |
| 5795075 | Clc1ccc(OCc2nc(-c3ccccn3)no2)c(Br)c1 | no |
| 7115521 | O=C1CCCc2c1[nH]c1ccc(C(F)(F)F)cc21 | no |

**Supplementary Table 4. Continued**

| Chemical Name | SMILES | Retested |
|---|---|---|
| 7145248 | O=C(NCc1cccc(Oc2ccccc2)c1)C(=O)c1c[nH]c2ccccc12 | yes |
| 7015047 | Cc1ccc(C(C)C)c(OCCNCc2ccccc2)c1 | no |
| 5802987 | Clc1ccc(OCc2nc(-c3cccnc3)no2)c(Br)c1 | no |
| 5982161 | COC(=O)c1c(N=C(O)CCN2C(=O)c3ccccc3C2=O)sc2c1CCC2 | no |
| 6647373 | CC(=O)Oc1ccc2c(=O)c(Oc3ccc(F)cc3)c(C(F)(F)F)oc2c1C | no |
| 7112518 | CCOC(=O)COc1ccc2c(c1)c(C(C)=O)c(C)n2Cc1ccccc1 | no |
| 6653154 | CC(=O)Oc1ccc2c(=O)c(Oc3ccccc3)c(C(F)(F)F)oc2c1C | no |
| 6689594 | CCc1cc2c(=O)c(Oc3ccccc3F)c(C)oc2cc1OC(C)=O | no |
| 6793728 | Cc1cc(=Nc2cccc3ccccc23)c2cc(Cl)ccc2[nH]1.Cl | no |
| 5846232 | CC(=O)c1cc(F)c(N2CCN(C(=O)c3ccc(Cl)cc3Cl)CC2)cc1C | yes |
| 6639477 | COc1ccc2c(=O)c(-c3ccccc3Cl)c(C)oc2c1 | no |
| 5128592 | Cc1cccc(NC(=O)c2ccc(C(C)(C)C)cc2)c1 | yes |
| 6216715 | Cc1cccc(C)c1N=C(O)CSCc1cccc(Br)c1 | no |
| 6053334 | O=C(Cc1cccc(Cl)c1)Nc1ccc(Cl)cc1 | no |
| 6646535 | COc1ccc2c(=O)c(-c3ccccc3)c(C)oc2c1C | no |
| 6367818 | CCC(=O)Nc1ccc(Oc2ccc(Cl)cc2)cc1 | no |
| 7115235 | CC(C)(C)C(=O)CSc1nc2ccccc2c(=O)n1Cc1ccccc1 | no |
| promazine | CN(C)CCCN1c2ccccc2Sc2ccccc21 | no |
| 6661919 | CCCCN=C(S)N=C(O)c1ccc(Cl)cc1Cl | yes |
| 6163607 | O=S(=O)(Nc1ccc(OCc2ccccc2)cc1)c1ccc2c(c1)OCCO2 | yes |
| 5690842 | COC(=O)c1sc(N=C(O)C2CCCO2)c(C(=O)OC)c1C | yes |
| 6673619 | Cc1ccccc1CC(=O)c1ccc(O)c(C)c1O | no |
| 7011253 | COc1ccccc1-c1nnc(SCc2ccccc2F)n1-c1ccccc1 | no |
| 7150160 | O=C(CC(CC(=O)c1ccccc1)c1ccccc1)c1ccccc1 | no |
| 5867832 | COc1ccc(N=C(O)c2ccccc2Oc2ccccc2)c(OC)c1 | no |
| 5942595 | Cc1ccc(Cl)cc1N=C(O)COc1c(C)cccc1C | yes |
| 6150813 | Cc1noc(C)c1CSc1nc2sc3c(c2c(=O)n1-c1ccccc1)CCC3 | no |
| 6165550 | Cc1cccc(-n2c(C=Cc3cccc([N+](=O)[O-])c3)nc3ccccc3c2=O)c1 | no |
| 6678692 | CCc1cc2c(=O)c(Oc3ccc(F)cc3)coc2cc1OC(C)=O | no |
| 7237541 | Cc1ccc2c3c1C(=O)C(=O)N3C(C)(C)CC2(C)c1ccccc1 | yes |

**Supplementary Table 5. Compound names and summary of results from *in vitro* FLIPR experiments for GABAA.**

| Compound Name | FLIPR Results | Ave Norm FLIPR Score | SD |
|---|---|---|---|
| DMSO | Negative | 1 | 0.992 |
| BGC 20-761 | Negative | 1.188647747 | 3.043 |
| picrotoxin | Negative | -9.759599332 | 2.087 |
| progesterone | Positive | 17.80300501 | 4.5 |
| etomidate | Positive | 50.67111853 | 0.892 |
| tracazolate | Positive | 50.13856428 | 2.255 |
| propofol | Positive | 34.52337229 | 8.439 |
| thiopental | Positive | 11.39232053 | 2.274 |
| DOC | Negative | -4.308013356 | 2.091 |
| diazepam | Negative | -10.89565944 | 1.052 |
| 7013338 | Positive | 116.3580968 | 3.651 |
| 5942595 | Positive | 84.9148581 | 6.339 |
| 5658603 | Positive | 55.23539232 | 3.39 |
| 6474599 | Positive | 52.02420702 | 3.831 |
| 6537142 | Positive | 50.97579299 | 1.64 |
| 5649824 | Positive | 41.41402337 | 4.859 |
| 5583877 | Positive | 36.70784641 | 3.964 |
| 5925611 | Positive | 32.94657763 | 0.645 |
| 6376886 | Positive | 32.44240401 | 6.486 |
| 5695025 | Positive | 32.2721202 | 4.675 |
| 6028165 | Positive | 28.75792989 | 3.955 |
| 5573728 | Positive | 27.62103506 | 2.061 |
| 5860357 | Positive | 25.8706177 | 3.621 |
| 6993015 | Positive | 23.92654424 | 2.021 |
| 5937132 | Positive | 20.70033389 | 3.122 |
| 7282929 | Positive | 17.15317195 | 2.717 |
| 7114005 | Positive | 15.23205343 | 0.272 |
| 5869570 | Positive | 15.03672788 | 0.574 |
| 6366118 | Positive | 14.5033389 | 0.336 |
| 6029941 | Positive | 14.00584307 | NA |
| 5690842 | Positive | 12.09432387 | 6.775 |
| 7273455 | Positive | 7.892320535 | 5.349 |
| 7100598 | Positive | 7.736227046 | 0.217 |
| 6091285 | Positive | 7.664440735 | 0.253 |
| 5986291 | Positive | 6.420701169 | 4.018 |
| 5142031 | Positive | 5.23706177 | 0.349 |
| 6030006 | Negative | 3.04590985 | 3.264 |
| 5768306 | Negative | 2.243739566 | 0.059 |
| 6013263 | Negative | 1.035058431 | 2.342 |

**Supplementary Table 5. Continued**

| Compound Name | FLIPR Results | Ave Norm FLIPR Score | SD |
|---|---|---|---|
| 5102870 | Negative | -0.944908181 | 1.695 |
| 7166683 | Negative | -1.302170284 | 0.581 |
| 5811265 | Negative | -1.4557596 | 3.792 |
| 5551268 | Negative | -1.463272121 | 1.876 |
| 7113584 | Negative | -2.454924875 | 0.79 |
| 5835629 | Negative | -2.682804675 | 3.707 |
| 5845856 | Negative | -2.947412354 | 1.101 |
| 6645327 | Negative | -4.733722872 | 0.776 |
| 6212662 | Negative | -5.178631052 | 0.047 |
| 5846886 | Negative | -5.25542571 | 0.94 |
| 6163607 | Negative | -5.679465776 | 1.265 |
| 7014338 | Negative | -6.270450752 | 2.581 |
| 6246227 | Negative | -6.910684474 | 1.718 |
| 7284610 | Negative | -7.29966611 | 4.074 |
| 6366421 | Negative | -8.515859767 | 1.221 |
| 5736224 | Negative | -10.45158598 | 1.238 |
| 6225936 | Negative | -14.696995 | 0.004 |
| 7145248 | Negative | -24.78631052 | 0.673 |
| 5587256 | Negative | -26.36894825 | 22.82 |

**Supplementary Table 6. SEA predictions prioritized by EF from the top 1000 hit compounds.**

| CHEMBL ID | EF | p-Value | q-Value | Description |
|---|---|---|---|---|
| CHEMBL5469 | 10.832 | 2.82E-153 | 9.38E-148 | Protein tyrosine kinase 2 beta |
| CHEMBL2094122 | 9.2246 | 3.67E-20 | 1.22E-14 | GABA-A receptor; alpha-5/beta-3/gamma-2 |
| CHEMBL3746 | 8.2498 | 5.69E-90 | 1.89E-84 | 11-beta-hydroxysteroid dehydrogenase 2 |
| CHEMBL3012 | 6.1087 | 6.66E-54 | 2.22E-48 | Phosphodiesterase 7A |
| CHEMBL4501 | 5.2561 | 9.62E-22 | 3.20E-16 | Ribosomal protein S6 kinase 1 |
| CHEMBL5936 | 5.2175 | 8.15E-29 | 2.71E-23 | Toll-like receptor 7 |
| CHEMBL1907607 | 5.16 | 1.14E-53 | 3.79E-48 | GABA-A receptor; anion channel |
| CHEMBL4409 | 4.6661 | 1.65E-09 | 0.000549 | Phosphodiesterase 10A |
| CHEMBL2835 | 4.3081 | 2.33E-56 | 7.74E-51 | Tyrosine-protein kinase JAK1 |
| CHEMBL1787 | 4.3081 | 4.35E-30 | 1.45E-24 | Steroid 5-alpha-reductase 1 |
| CHEMBL2095227 | 4.2252 | 3.99E-178 | 1.33E-172 | Vascular endothelial growth factor receptor |
| CHEMBL4296 | 4.2035 | 1.41E-35 | 4.69E-30 | Sodium channel protein type IX alpha subunit |
| CHEMBL3055 | 4.1484 | 9.87E-10 | 0.000328 | Cyclin-dependent kinase 7 |
| CHEMBL4975 | 4.0012 | 8.05E-14 | 2.68E-08 | Adenosine A1 receptor |
| CHEMBL2034 | 3.827 | 6.36E-34 | 2.12E-28 | Glucocorticoid receptor |
| CHEMBL1856 | 3.5805 | 9.87E-79 | 3.28E-73 | Steroid 5-alpha-reductase 2 |
| CHEMBL4977 | 3.5709 | 8.07E-63 | 2.69E-57 | Proto-oncogene c-JUN |
| CHEMBL2488 | 3.5637 | 3.50E-43 | 1.16E-37 | Prostanoid EP2 receptor |
| CHEMBL4040 | 3.4143 | 7.31E-47 | 2.43E-41 | MAP kinase ERK2 |
| CHEMBL259 | 3.3269 | 5.86E-17 | 1.95E-11 | Melanocortin receptor 4 |
| CHEMBL3072 | 3.2333 | 7.50E-24 | 2.49E-18 | Androgen Receptor |
| CHEMBL1907605 | 3.1718 | 1.64E-15 | 5.46E-10 | Cyclin-dependent kinase 2/cyclin E1 |
| CHEMBL1918 | 3.0062 | 8.12E-184 | 2.70E-178 | Glutamate receptor ionotropic kainate 1 |
| CHEMBL2337 | 2.8332 | 9.25E-110 | 3.08E-104 | Glycine transporter 1 |
| CHEMBL3687 | 2.7944 | 2.78E-49 | 9.26E-44 | Arachidonate 12-lipoxygenase |
| CHEMBL4430 | 2.7913 | 4.86E-43 | 1.62E-37 | Cytochrome P450 17A1 |
| CHEMBL330 | 2.7913 | 3.71E-67 | 1.24E-61 | Glutamate (NMDA) receptor subunit zeta 1 |
| CHEMBL3514 | 2.7732 | 4.06E-30 | 1.35E-24 | LDL-associated phospholipase A2 |
| CHEMBL5652 | 2.7499 | 2.49E-110 | 8.27E-105 | Glucose-dependent insulinotropic receptor |
| CHEMBL2569 | 2.7405 | 3.98E-110 | 1.32E-104 | Microsomal triglyceride transfer protein large subunit |
| CHEMBL2971 | 2.6595 | 2.83E-47 | 9.41E-42 | Tyrosine-protein kinase JAK2 |
| CHEMBL5658 | 2.6443 | 6.50E-66 | 2.16E-60 | Prostaglandin E synthase |
| CHEMBL1293255 | 2.6013 | 8.60E-140 | 2.86E-134 | 15-hydroxyprostaglandin dehydrogenase [NAD+] |
| CHEMBL1889 | 2.5984 | 4.47E-68 | 1.49E-62 | Vasopressin V1a receptor |
| CHEMBL2568 | 2.5325 | 5.69E-92 | 1.89E-86 | Liver glycogen phosphorylase |
| CHEMBL3230 | 2.4991 | 3.40E-103 | 1.13E-97 | Sphingosine 1-phosphate receptor Edg-6 |
| CHEMBL4036 | 2.454 | 2.29E-26 | 7.61E-21 | Cyclin-dependent kinase 5 |
| CHEMBL2903 | 2.4465 | 7.17E-17 | 2.39E-11 | Arachidonate 15-lipoxygenase |
| CHEMBL3227 | 2.4305 | 6.64E-166 | 2.21E-160 | Metabotropic glutamate receptor 5 |
| CHEMBL1790 | 2.3894 | 1.62E-28 | 5.39E-23 | Vasopressin V2 receptor |
| CHEMBL275 | 2.3347 | 2.22E-16 | 7.40E-11 | Phosphodiesterase 4B |
| CHEMBL4652 | 2.3307 | 3.35E-40 | 1.11E-34 | Somatostatin receptor 1 |
| CHEMBL3371 | 2.2818 | 1.39E-12 | 4.62E-07 | Serotonin 6 (5-HT6) receptor |
| CHEMBL5409 | 2.2229 | 7.73E-26 | 2.57E-20 | G-protein coupled bile acid receptor 1 |
| CHEMBL3351 | 2.1837 | 2.62E-12 | 8.72E-07 | Acetyl-CoA carboxylase 1 |
| CHEMBL4336 | 2.1425 | 6.56E-15 | 2.18E-09 | Prostanoid EP3 receptor |
| CHEMBL235 | 2.1375 | 3.50E-48 | 1.16E-42 | Peroxisome proliferator-activated receptor gamma |
| CHEMBL2095160 | 2.1118 | 2.42E-09 | 0.000807 | Leukotriene B4 receptor |
| CHEMBL5071 | 2.0202 | 2.18E-57 | 7.25E-52 | G protein-coupled receptor 44 |
| CHEMBL1811 | 1.9836 | 1.35E-19 | 4.51E-14 | Prostanoid EP1 receptor |
| CHEMBL4315 | 1.9642 | 1.48E-22 | 4.93E-17 | Purinergic receptor P2Y1 |

**Supplemental Table 6. Continued (2 of 3)**

| CHEMBL ID | EF | p-Value | q-Value | Description |
|---|---|---|---|---|
| CHEMBL1906 | 1.9542 | 1.78E-33 | 5.91E-28 | Serine/threonine-protein kinase RAF |
| CHEMBL2001 | 1.9359 | 1.07E-72 | 3.55E-67 | Purinergic receptor P2Y12 |
| CHEMBL4478 | 1.9234 | 9.22E-19 | 3.07E-13 | Voltage-gated N-type calcium channel alpha-1B subunit |
| CHEMBL3338 | 1.9062 | 1.44E-28 | 4.79E-23 | Squalene synthetase |
| CHEMBL1966 | 1.8407 | 7.61E-12 | 2.53E-06 | Dihydroorotate dehydrogenase |
| CHEMBL2993 | 1.8048 | 1.81E-19 | 6.01E-14 | Monoamine oxidase B |
| CHEMBL4051 | 1.7585 | 8.50E-33 | 2.83E-27 | Cystic fibrosis transmembrane conductance regulator |
| CHEMBL3974 | 1.7366 | 3.74E-29 | 1.24E-23 | Proteinase-activated receptor 1 |
| CHEMBL2868 | 1.7226 | 1.58E-63 | 5.24E-58 | Vasopressin V1a receptor |
| CHEMBL1868 | 1.694 | 3.93E-17 | 1.31E-11 | Vascular endothelial growth factor receptor 1 |
| CHEMBL2736 | 1.6741 | 1.24E-32 | 4.14E-27 | Metabotropic glutamate receptor 4 |
| CHEMBL244 | 1.6674 | 2.31E-22 | 7.70E-17 | Coagulation factor X |
| CHEMBL3553 | 1.6663 | 4.38E-44 | 1.46E-38 | Tyrosine-protein kinase TYK2 |
| CHEMBL285 | 1.6627 | 2.46E-10 | 8.20E-05 | Acyl coenzyme A:cholesterol acyltransferase 1 |
| CHEMBL1901 | 1.6571 | 2.88E-28 | 9.58E-23 | Cholecystokinin A receptor |
| CHEMBL1741186 | 1.6522 | 2.90E-36 | 9.66E-31 | Nuclear receptor ROR-gamma |
| CHEMBL311 | 1.6361 | 5.80E-22 | 1.93E-16 | Glutamate [NMDA] receptor subunit epsilon 2 |
| CHEMBL3969 | 1.6281 | 2.92E-10 | 9.72E-05 | Carbonic anhydrase VB |
| CHEMBL4892 | 1.6142 | 6.42E-10 | 0.000213 | Alpha-1a adrenergic receptor |
| CHEMBL3238 | 1.6083 | 4.80E-75 | 1.60E-69 | Carnitine palmitoyltransferase 2 |
| CHEMBL249 | 1.6064 | 1.82E-46 | 6.06E-41 | Neurokinin 1 receptor |
| CHEMBL3766 | 1.5872 | 3.26E-14 | 1.09E-08 | Vasopressin V2 receptor |
| CHEMBL2413 | 1.5732 | 1.63E-13 | 5.43E-08 | C-C chemokine receptor type 1 |
| CHEMBL2047 | 1.5349 | 1.17E-10 | 3.88E-05 | Bile acid receptor FXR |
| CHEMBL3858 | 1.5283 | 7.25E-56 | 2.41E-50 | Carnitine palmitoyltransferase 1A |
| CHEMBL2564 | 1.5215 | 5.48E-46 | 1.82E-40 | Metabotropic glutamate receptor 5 |
| CHEMBL1913 | 1.4884 | 1.72E-11 | 5.72E-06 | Platelet-derived growth factor receptor beta |
| CHEMBL1293194 | 1.4697 | 1.28E-46 | 4.25E-41 | Carnitine O-palmitoyltransferase 1 liver isoform |
| CHEMBL2095150 | 1.4663 | 4.96E-23 | 1.65E-17 | Phosphodiesterase 1 |
| CHEMBL3156 | 1.457 | 8.36E-13 | 2.78E-07 | Thromboxane A2 receptor |
| CHEMBL4018 | 1.4434 | 3.32E-17 | 1.10E-11 | Neuropeptide Y receptor type 2 |
| CHEMBL1844 | 1.3767 | 3.24E-17 | 1.08E-11 | Macrophage colony stimulating factor receptor |
| CHEMBL2216739 | 1.3745 | 2.01E-23 | 6.68E-18 | Carnitine O-palmitoyltransferase 1 muscle isoform |
| CHEMBL4722 | 1.3586 | 2.22E-13 | 7.40E-08 | Serine/threonine-protein kinase Aurora-A |
| CHEMBL2093869 | 1.3358 | 4.11E-15 | 1.37E-09 | Integrin alpha-IIb/beta-3 |
| CHEMBL2093866 | 1.3354 | 6.69E-25 | 2.23E-19 | Estrogen receptor |
| CHEMBL5669 | 1.2425 | 3.27E-24 | 1.09E-18 | Epoxide hydrolase 2 |
| CHEMBL4140 | 1.2125 | 3.30E-37 | 1.10E-31 | Epoxide hydratase |
| CHEMBL256 | 1.1181 | 3.59E-12 | 1.19E-06 | Adenosine A3 receptor |
| CHEMBL3572 | 1.0034 | 8.31E-15 | 2.77E-09 | Cholesteryl ester transfer protein |
| CHEMBL3105 | 0.89889 | 1.50E-20 | 4.99E-15 | Poly [ADP-ribose] polymerase-1 |
| CHEMBL1937 | 0.86861 | 2.11E-25 | 7.01E-20 | Histone deacetylase 2 |
| CHEMBL1951 | 0.8684 | 3.01E-09 | 0.001 | Monoamine oxidase A |
| CHEMBL255 | 0.85701 | 2.34E-19 | 7.79E-14 | Adenosine A2b receptor |
| CHEMBL2111429 | 0.8541 | 7.13E-15 | 2.37E-09 | Histone deacetylase (HDAC1 and HDAC2) |
| CHEMBL3254 | 0.82456 | 9.65E-20 | 3.21E-14 | Monoamine oxidase A |
| CHEMBL273 | 0.82284 | 3.24E-17 | 1.08E-11 | Serotonin 1a (5-HT1a) receptor |
| CHEMBL2095189 | 0.78671 | 5.92E-11 | 1.97E-05 | Platelet-derived growth factor receptor |
| CHEMBL251 | 0.78053 | 3.07E-14 | 1.02E-08 | Adenosine A2a receptor |
| CHEMBL321 | 0.77988 | 1.51E-14 | 5.02E-09 | Matrix metalloproteinase 9 |
| CHEMBL6009 | 0.76082 | 2.88E-15 | 9.60E-10 | Diacylglycerol O-acyltransferase 1 |

**Supplemental Table 6. Continued (3 of 3)**

| CHEMBL ID | EF | p-Value | q-Value | Description |
|---|---|---|---|---|
| CHEMBL4191 | 0.74784 | 1.01E-11 | 3.36E-06 | Monoglyceride lipase |
| CHEMBL205 | 0.73443 | 1.20E-10 | 3.99E-05 | Carbonic anhydrase II |
| CHEMBL3571 | 0.73416 | 2.94E-14 | 9.79E-09 | Cannabinoid CB1 receptor |
| CHEMBL3192 | 0.72428 | 1.63E-38 | 5.42E-33 | Histone deacetylase 8 |
| CHEMBL5393 | 0.71706 | 5.11E-59 | 1.70E-53 | ATP-binding cassette sub-family G member 2 |
| CHEMBL219 | 0.68396 | 5.99E-16 | 1.99E-10 | Dopamine D4 receptor |
| CHEMBL3594 | 0.68047 | 7.19E-21 | 2.39E-15 | Carbonic anhydrase IX |
| CHEMBL281 | 0.67296 | 3.75E-19 | 1.25E-13 | Carbonic anhydrase IV |
| CHEMBL1945 | 0.66941 | 1.07E-24 | 3.57E-19 | Melatonin receptor 1A |
| CHEMBL1980 | 0.6652 | 2.77E-10 | 9.22E-05 | Sodium channel protein type V alpha subunit |
| CHEMBL332 | 0.65466 | 2.02E-22 | 6.71E-17 | Matrix metalloproteinase-1 |
| CHEMBL4302 | 0.65163 | 1.11E-12 | 3.71E-07 | P-glycoprotein 1 |
| CHEMBL3138 | 0.64379 | 1.19E-20 | 3.98E-15 | Dopamine D3 receptor |
| CHEMBL246 | 0.62469 | 5.68E-10 | 0.000189 | Beta-3 adrenergic receptor |
| CHEMBL261 | 0.62233 | 6.21E-24 | 2.07E-18 | Carbonic anhydrase I |
| CHEMBL2095171 | 0.58411 | 9.03E-10 | 0.0003 | Sodium channel alpha subunits; brain (Types I II III) |
| CHEMBL3180 | 0.58231 | 1.61E-12 | 5.36E-07 | Carboxylesterase 2 |
| CHEMBL2093865 | 0.57073 | 9.07E-25 | 3.02E-19 | Histone deacetylase |
| CHEMBL4792 | 0.55951 | 8.64E-24 | 2.87E-18 | Orexin receptor 2 |
| CHEMBL2093870 | 0.55882 | 4.04E-20 | 1.34E-14 | Serotonin 2 (5-HT2) receptor |
| CHEMBL260 | 0.55449 | 2.22E-15 | 7.37E-10 | MAP kinase p38 alpha |
| CHEMBL3898 | 0.55037 | 1.05E-20 | 3.50E-15 | Bone morphogenetic protein 1 |
| CHEMBL1878 | 0.54726 | 1.45E-14 | 4.82E-09 | Calcium sensing receptor |
| CHEMBL2096671 | 0.53752 | 1.76E-11 | 5.86E-06 | Serotonin 2 (5-HT2) receptor |
| CHEMBL3229 | 0.53436 | 5.63E-21 | 1.87E-15 | Anandamide amidohydrolase |
| CHEMBL3473 | 0.53004 | 2.87E-34 | 9.54E-29 | C-C chemokine receptor type 3 |
| CHEMBL234 | 0.52084 | 4.19E-46 | 1.39E-40 | Dopamine D3 receptor |
| CHEMBL1946 | 0.51445 | 3.27E-42 | 1.09E-36 | Melatonin receptor 1B |
| CHEMBL214 | 0.51376 | 1.16E-28 | 3.84E-23 | Serotonin 1a (5-HT1a) receptor |
| CHEMBL3455 | 0.5133 | 1.69E-28 | 5.62E-23 | Anandamide amidohydrolase |
| CHEMBL2094268 | 0.50345 | 2.06E-31 | 6.84E-26 | Melatonin receptor |
| CHEMBL283 | 0.50011 | 9.33E-46 | 3.10E-40 | Matrix metalloproteinase 3 |
| CHEMBL3361 | 0.49714 | 4.58E-39 | 1.52E-33 | Dopamine D4 receptor |
| CHEMBL3427 | 0.49437 | 2.44E-14 | 8.12E-09 | Dopamine D2 receptor |
| CHEMBL264 | 0.49369 | 5.49E-16 | 1.83E-10 | Histamine H3 receptor |
| CHEMBL217 | 0.48105 | 2.23E-52 | 7.43E-47 | Dopamine D2 receptor |
| CHEMBL3465 | 0.48013 | 9.07E-15 | 3.02E-09 | Sigma opioid receptor |
| CHEMBL220 | 0.47337 | 8.15E-17 | 2.71E-11 | Acetylcholinesterase |
| CHEMBL2409 | 0.44961 | 2.31E-28 | 7.68E-23 | Epoxide hydratase |
| CHEMBL4588 | 0.4342 | 2.36E-49 | 7.85E-44 | Matrix metalloproteinase 8 |
| CHEMBL1873 | 0.42696 | 3.40E-20 | 1.13E-14 | Tissue-type plasminogen activator |
| CHEMBL325 | 0.42455 | 3.61E-39 | 1.20E-33 | Histone deacetylase 1 |
| CHEMBL3286 | 0.41945 | 7.47E-29 | 2.49E-23 | Urokinase-type plasminogen activator |
| CHEMBL287 | 0.39167 | 2.26E-39 | 7.51E-34 | Sigma opioid receptor |
| CHEMBL3223 | 0.37809 | 4.88E-32 | 1.62E-26 | Serotonin 7 (5-HT7) receptor |
| CHEMBL3199 | 0.36708 | 2.07E-29 | 6.88E-24 | Acetylcholinesterase |
| CHEMBL3602 | 0.33883 | 6.17E-42 | 2.05E-36 | Sigma opioid receptor |
| CHEMBL3198 | 0.3105 | 2.27E-38 | 7.55E-33 | Acetylcholinesterase |

**Supplementary Table 7. SEA predictions prioritized by EF from the top 30 hit compounds.**

| CHEMBL ID | EF | p-Value | q-Value | description |
|---|---|---|---|---|
| CHEMBL3746 | 53.234 | 0 | 0 | 11-beta-hydroxysteroid dehydrogenase 2 |
| CHEMBL1787 | 27.799 | 7.30E-267 | 2.43E-261 | Steroid 5-alpha-reductase 1 |
| CHEMBL3072 | 20.864 | 1.26E-225 | 4.18E-220 | Androgen Receptor |
| CHEMBL1856 | 19.253 | 5.42E-146 | 1.80E-140 | Steroid 5-alpha-reductase 2 |
| CHEMBL1907607 | 14.798 | 6.99E-127 | 2.33E-121 | GABA-A receptor; anion channel |
| CHEMBL2096664 | 8.055 | 2.14E-54 | 7.12E-49 | Steroid 5-alpha-reductase |
| CHEMBL230 | 7.6613 | 1.04E-24 | 3.47E-19 | Cyclooxygenase-2 |
| CHEMBL2903 | 5.92 | 1.39E-48 | 4.61E-43 | Arachidonate 15-lipoxygenase |
| CHEMBL2095227 | 4.8113 | 2.18E-125 | 7.25E-120 | Vascular endothelial growth factor receptor |
| CHEMBL1889 | 4.5727 | 3.51E-99 | 1.17E-93 | Vasopressin V1a receptor |
| CHEMBL1918 | 4.4766 | 5.57E-125 | 1.85E-119 | Glutamate receptor ionotropic kainate 1 |
| CHEMBL3371 | 4.4172 | 2.91E-50 | 9.70E-45 | Serotonin 6 (5-HT6) receptor |
| CHEMBL2564 | 4.0909 | 1.99E-49 | 6.60E-44 | Metabotropic glutamate receptor 5 |
| CHEMBL2337 | 3.6564 | 5.47E-147 | 1.82E-141 | Glycine transporter 1 |
| CHEMBL2568 | 3.4403 | 2.69E-29 | 8.97E-24 | Liver glycogen phosphorylase |
| CHEMBL244 | 3.3105 | 3.62E-30 | 1.20E-24 | Coagulation factor X |
| CHEMBL2993 | 2.9115 | 2.77E-20 | 9.22E-15 | Monoamine oxidase B |
| CHEMBL3230 | 2.8458 | 8.52E-70 | 2.83E-64 | Sphingosine 1-phosphate receptor Edg-6 |
| CHEMBL1966 | 2.6394 | 3.89E-14 | 1.29E-08 | Dihydroorotate dehydrogenase |
| CHEMBL2868 | 2.6154 | 1.21E-59 | 4.04E-54 | Vasopressin V1a receptor |
| CHEMBL3238 | 2.5944 | 7.37E-27 | 2.45E-21 | Carnitine palmitoyltransferase 2 |
| CHEMBL3766 | 2.5604 | 2.80E-50 | 9.32E-45 | Vasopressin V2 receptor |
| CHEMBL2216739 | 2.2174 | 5.63E-11 | 1.87E-05 | Carnitine O-palmitoyltransferase 1 muscle isoform |
| CHEMBL4681 | 2.0292 | 2.43E-10 | 8.09E-05 | Aldo-keto-reductase family 1 member C3 |

**Supplementary Table 8. Reference compounds targeting receptors with relatively low EF scores**

| Compound | Target | InChiKey |
|---|---|---|
| SR 49059 | V1 antagonist | CEBYCSRFKCEUSW-NAYZPBBASA-N |
| TCS 359 | FLT3 RTK antagonist | FSPQCTGGIANIJZ-UHFFFAOYSA-N |
| Piclamilast | PDEantagonist | RRRUXBQSQLKHEL-UHFFFAOYSA-N |
| ( R)-C R 8 | CDK antagonist | ORYSYXHQFOWNDK-RGFWRHHQSA-N |
| DL-TBOA | EAAT antagonist | BYOBCYXURWDEDS-IUCAKERBSA-N |
| ML 218 hydrochloride | CAC1G antagonist | IDCVEUISZZKMKJ-ZXVFAPHLSA-N |
| AG 825 | Erbb2 antagonist | KXDONFLNGBQLTN-WUXMJOGZSA-N |
| Tak 165 | Erbb2 antagonist | ZTFBIUXIQYRUNT-MDWZMJQESA-N |
| Z57705210 | PPBT antagonist | OTFKFIWLDAHEKF-UHFFFAOYSA-N |
| BX 471 | CCR1 antagonist | XQYASZNUFDVMFH-CQSZACIVSA-N |
| ML 00253764 | MC4R antagonist | KZUMGPQDDCBFBF-UHFFFAOYSA-N |
| Nav26 | SCN9A antagonist | ICGMZCVSHDKQTE-UHFFFAOYSA-N |
| ATC 0065 | MCHR1 antagonist | BPGUWYBAINNZQH-LFOVFOEYSA-N |
| 5648949 | PD2R2 antagonist | WVBVIRRWTMFJAK-AWQFTUOYSA-N |
| JQ1 | BRD3 antagonist | DNVXATUJJDPFDM-KRWDZBQOSA-N |
| R-+-methanandamide | CNR1 agonist | SQKRUBZPTNJQEM-FQPARAGTSA-N |
| TOPIRAMATE | GluR5 antagonist | KJADKKWYZYXHBB-XBWDGYHZSA-N |
| ML 202 | PKM2 antagonist | MORBXZMIXGYQDB-UHFFFAOYSA-N |
| PF 04885614 | SCNAA antagonist | AGORGFNWYAUYSU-UHFFFAOYSA-N |
| LY 341495 | Group II mGluR antagonist | VLZBRVJVCCNPRJ-KPHUOKFYSA-N |
| ICI 63197 | PDE4 antagonist | UQDVRVNMIJAGRK-UHFFFAOYSA-N |
| GW 791343 Hydrochloride | P2X7 allosteric modulator | WSBRAHWNJBXXJM-UHFFFAOYSA-N |
| A 740003 | P2X7 antagonist | PUHSRMSFDASMAE-UHFFFAOYSA-N |
| TOLMETIN | IL-8 antagonist | UPSPUYADGBWSHF-UHFFFAOYSA-N |
| EMDT oxalate | 5HT6 Agonist | IFGWAHGHGDZBEH-UHFFFAOYSA-N |
| Metyrapone | 11β-hydroxylase antagonist | FJLBFSROUSIWMA-UHFFFAOYSA-N |
| ACEA | CB1 receptor agonist | SCJNCDSAIRBRIA-DOFZRALJSA-N |
| Strophanthidin | Na+ /K+-ATPase antagonist | ODJLBQGVINUMMR-HZXDTFASSA-N |
| Procaine | NaV general antagonist | MFDFERRIHVXMIY-UHFFFAOYSA-N |
| ZINC13108136 | BACE2 antagonist | SVHVIRBGQRDCIB-UHFFFAOYSA-N |
| ATC 0175 hydrochloride | MCH1 antagonist | FAIMGWSOSCFGRU-UHFFFAOYSA-N |
| Fenobam | mGlu5 agonist | DWPQODZAOSWNHB-UHFFFAOYSA-N |
| NNC 55-0396 dihydrochloride | NaV general antagonist | BCCQNBXHUMKLFW-HNQRYHMESA-N |
| Xylazine hydrochloride | α2-adronergic agonist | DIIBRMSCONGGIN-UHFFFAOYSA-N |
| Bumetanide | NKCC cotransporter antagonist | MAEIEVLCKWDQJH-UHFFFAOYSA-N |
| Epibatidine Hydrochloride | nicotinic agonist | NLPRAJRHRHZCQQ-UTLUCORTSA-N |
| (-)-Nicotine Ditartrate | Nicotinic acetylcholine receptor agonis | RFEJUZJILGIRHQ-UHFFFAOYSA-N |
| Hexamethonium Bromide | nicotinic receptor antagonist | FAPSXSAPXXJTOU-UHFFFAOYSA-L |
| Scopolomine hydrobromide | muscarinic antagonist | STECJAGHUSJQJN-FWXGHANASA-N |
| octopamine hydrochloride | β3 adrenoceptor agonist | QHGUCRYDKWKLMG-UHFFFAOYSA-N |
| Ro 32-0432 hydrochloride | protein kinase C antagonist | HSPRASOZRZDELU-LMOVPXPDSA-N |
| BzATP triethylammonium salt | P2X7 receptor agonist | HVOVBTNCGADRTH-WBLDMZOZSA-N |
| Phenytoin | NaV general antagonist | CXOFVDLJLONNDW-UHFFFAOYSA-N |
| TCB-2 | 5HT2AR agonist | TYMMXVZAUGQKRF-UHFFFAOYSA-N |
| MTEP | mGluR5 antagonist | NRBNGHCYDWUVLC-UHFFFAOYSA-N |
| Ro4368554 | 5HT6R antagonist | AOPYPEADLGTXRA-UHFFFAOYSA-N |
| Biphenyl-indanone A | mGluR2 allosteric modulator | KMKBEESNZAPKMP-UHFFFAOYSA-N |
| LuAE58054 | 5HT6R antagonist | YBAWYTYNMZWMMJ-UHFFFAOYSA-N |

**Supplementary Table 9. Compound names and descriptions for annotated and novel small molecules that interact with 3 of the primary SEA predicted targets (mGluR, GABAA, and HTR6).**

| mGluR Compounds tested | SMILES | Mechanism Notes | Phenoscore |
|---|---|---|---|
| Fenobam | CN1CC(=O)N=C1NC(=O)Nc2cccc(c2)Cl | mGlu5 agonist | 0.3 |
| STK234931 | c1ccc(cc1)C#Cc2ccc(cc2)C(=O)N3CCC(CC3)O | MGluR antagonist | 0.4 |
| MPEP | CC1=NC(=CC=C1)C#CC2=CC=CC=C2.Cl | mGlu5 antagonist | 0.73 |
| TOPIRAMATE | CC1(O[C@@H]2CO[C@@]3([C@H]([C@@H]2O1)OC(O3)(C)C)COS(=O)(=O)N)C | mGluR5 antagonist | 0.1 |
| UBP 302 | c1ccc(c(c1)Cn2c(=O)ccn(c2=O)C[C@@H](C(=O)O)N)C(=O)O | mGluR5 antagonist | 0.17 |
| VU 0361737 | COc1cc(ccc1Cl)NC(=O)c2ccccn2 | mGluR4 positive allosteric modulator | 0.28 |
| VU 0155041 NaSalt | c1c(cc(cc1Cl)Cl)NC(=O)[C@H]2CCCC[C@H]2C(=O)O | mGluR4 positive allosteric modulator | 0.11 |
| Cinnabarinic acid | c1cc(c2c(c1)oc-3cc(=O)c(c(c3n2)C(=O)O)N)C(=O)O | mGluR4 agonist | 0.18 |
| L-AP4 | [H][C@](N)(CCP(O)(O)=O)C(O)=O | mGluR4/6/7/8 agonist | 0.1 |
| LY-354740 | C1C[C@]([C@H]2[C@@H]1[C@@H]2C(=O)O)(C(=O)O)N | mGluR2/3 agonist | 0.1 |
| Biphenylindanone A | CC1=C(C=C2CC(C(=O)C2=C1C)C3CCCC3)OCC4=CC(=CC=C4)C5=CC=C(C=C5)C(=O)O | mGluR2 positive allosteric modulator | 0.11 |

| Predicted mGluR | | | |
|---|---|---|---|
| 7285168 | OC(=Nc1ccc(Cl)cc1F)c1cccc2ccccc12 | predicted mGluR | 0.54 |
| 7211089 | O=[N+]([O-])c1cccc(N=C(O)c2ccc(Cl)cc2Cl)c1 | predicted mGluR | 0.5 |
| 5128592 | Cc1cccc(c1)NC(=O)c2ccc(cc2)C(C)(C)C | predicted mGluR | 0.52 |
| 6576466 | O=[N+]([O-])c1cccc(C(O)=Nc2ccc(Cl)cc2C(F)(F)F)c1 | predicted mGluR | 0.57 |
| 5795075 | Clc1ccc(OCc2nc(-c3ccccn3)no2)c(Br)c1 | predicted mGluR | 0.39 |
| 7136301 | O=C(N[C@H](c1occc1)C)COC(=O)c3cc([N+]([O-])=O)ccc3N2CCCC2 | predicted mGluR | 0.42 |
| 6587027 | CC(=O)c1cccc(NC(=O)c2ccc(-c3ccc(Cl)cc3)o2)c1 | predicted mGluR | 0.57 |
| 7271289 | Cc1cc(Cl)ccc1OCC(=O)N1CCN(c2ccc(Cl)cc2)CC1 | predicted mGluR | 0.56 |
| 7305598 | OC(=Nc1cccc(N=C(O)c2cc(Cl)ccc2Cl)c1)c1cc(Cl)ccc1Cl | predicted mGluR | 0.51 |
| 7100598 | O=C(c1ccccc1)c1cccc(N=C(O)Cc2ccc(Cl)cc2)c1 | predicted mGluR | 0.54 |
| 6053334 | O=C(Cc1cccc(Cl)c1)Nc1ccc(Cl)cc1 | predicted mGluR | 0.34 |
| 6376886 | COc1cc(OC)cc(C(O)=Nc2ccc(Cl)cc2C(F)(F)F)c1 | predicted mGluR | 0.59 |
| 5869570 | Cc1cc(nc(n1)N/C(=N\C(=O)C)/Nc2cccc(c2)C(F)(F)F)C | predicted mGluR | 0.68 |
| 5583877 | c1ccc(cc1)n2c(c(cn2)C(=O)Nc3ccc(cc3)Cl)NC(=O)c4ccco4 | predicted mGluR | 0.66 |
| 5943451 | CCOC(=O)c1c(C)n(Cc2ccco2)c2ccc(OC)cc21 | predicted mGluR | 0.37 |

| GABA Compounds Tested | | | |
|---|---|---|---|
| diazepam | CN1c2ccc(cc2C(=NCC1=O)c3ccccc3)Cl | GABAA agonist | 0.68 |
| propofol | Oc1c(cccc1C(C)C)C(C)C | GABAA agonist | 0.78 |
| DMCM | CCc1c(ncc2[nH]c3cc(OC)c(OC)cc3c12)C(=O)OC | GABAA negative allosteric modulator | 0.54 |
| β-CCE | CCOC(=O)c1cc2c3ccccc3[nH]c2cn1 | GABAA inverse agonist | 0.37 |
| flumazenil | CCOC(=O)c1ncn2c1CN(C)C(=O)c1cc(F)ccc1-2 | GABAA antagonist | 0.1 |
| TB 21007 | CC1(CC2=C(SC(=C2C(=O)C1)SCCO)C3=NC=CS3)C | GABAA inverse agonist | 0.12 |
| bicuculline (-) | CN1CCc2cc3c(cc2[C@H]1[C@H]4c5ccc6c(c5C(=O)O4)OCO6)OCO3 | GABAA antagonist | 0.05 |
| picrotoxin | C[C@@]12[C@H]3[C@H]4[C@H]([C@@H]([C@]1(C[C@@H]5[C@]2(O5)C(=O)O3)O)C(=O)O4)C(C)(C)O | GABAA antagonist | 0.03 |
| bicuculline (+) | CN1CCc2cc3c(cc2[C@H]1[C@H]4c5ccc6c(c5C(=O)O4)OCO6)OCO3 | GABAA antagonist | 0.23 |
| etomidate | N1(C=NC=C1C(=O)OCC)C(C)c2ccccc2 | GABAA agonist | 0.81 |
| Alphaxalone | CC(=O)[C@H]1CC[C@@H]2[C@@]1(CC(=O)[C@H]3[C@H]2CC[C@@H]4[C@@]3(CC[C@H](C4)O)C)C | GABAA agonist | 0.58 |
| thipoental | CCCC(C)C1(C(=O)NC(=S)NC1=O)CC | GABAA agonist | 0.51 |
| SR-95531 | [H]/N=c/1\ccc(nn1CCCC(=O)O)c2ccc(cc2)OC.Br | GABAA antagonist | 0.21 |
| muscimol | NCc1cc(O)no1 | GABAA agonist | 0.21 |
| GABA | NCCCC(=O)O | GABA agonist | 0.21 |
| tracazolate | CCCCNc1c2cnn(c2nc(c1C(=O)OCC)C)CC.Cl | GABAA agonist | 0.8 |
| clonazepam | c1ccc(c(c1)C2=NCC(=O)Nc3c2cc(cc3)[N+](=O)[O-])Cl | GABAA agonist | 0.23 |
| zolpidem | Cc1ccc(cc1)c2c(n3cc(ccc3n2)C)CC(=O)N(C)C | GABAA agonist | 0.23 |
| 21-Hydroxyprogesterone | C[C@]12CC[C@H]3[C@H]([C@@H]1CC[C@@H]2C(=O)CO)CCC4=CC(=O)CC[C@]34C | GABAA agonist | 0.79 |
| SCS | C1=CC=C(C(=C1)/C=N/NC(=O)C2=CC=CC=C2O)O | GABAA antagonist | 0.04 |
| Isoflurane | C(C(F)(F)F)(OC(F)F)Cl | GABAA agonist | 0.03 |
| TACA | C(/C=C/C(=O)O)N | GABAA agonist | 0.07 |
| R-Baclofen | c1cc(ccc1C(CC(=O)O)CN)Cl | GABAB Agonist | 0.26 |
| Progesterone | CC(=O)[C@H]1CCC2C3CCC4=CC(=O)CC[C@]4(C)C3CC[C@]12C | GABAA agonist | 0.68 |
| Indiplon | CC(=O)N(C)C1=CC=CC(=C1)C2=CC=NC3=C(C=NN23)C(=O)C4=CC=CS4 | GABA agonist | -0.06 |
| Ocinaplon | C1=CC=NC(=C1)C(=O)C2=C3N=CC=C(N3N=C2)C4=CC=NC=C4 | GABA agonist | -0.01 |
| L-655,708 | CCOC(=O)c1c2n(cn1)-c3ccc(cc3C(=O)N4[C@H]2CCC4)OC | GABAA antagonist | 0.51 |
| Gabapentin | C1CCC(CC1)(CC(=O)O)CN | increases GABA biosynthesis | 0.15 |
| Valproic Acid | CCCC(CCC)C(=O)O | GABA agonist | 0.75 |

| Predicted GABA | | | |
|---|---|---|---|
| 5658603 | CCCCn1c2ccccc2n2c(C(=O)OCC)c(C)nc12 | predicted GABAA | 0.74 |
| 5951201 | CCOC(=O)c1c(C)nc(-c2ccccc2)nc1N=C(O)Cc1ccccc1 | predicted GABAA | 0.48 |
| 5142031 | CCOC(=O)c1c(N)sc2ccccc12 | predicted GABAA | 0.54 |
| 7145248 | c1ccc(cc1)Oc2cccc(c2)CNC(=O)C(=O)c3c[nH]c4c3cccc4 | predicted GABAA | 0.5 |

| Serotonin-6 Compounds Tested | | | |
|---|---|---|---|
| BGC 20-761 | CN(C)CCC1=C(NC2=C1C=C(C=C2)OC)C3=CC=CC=C3 | 5-HT6 antagonist | 0.74 |
| Idalopirdine | C1=CC(=CC(=C1)OCC(C(F)(F)F)F)CNCCC2=CNC3=C2C=CC(=C3)F | 5-HT6 antagonist | 0.55 |
| R1485 DI HCL | c1ccc(c(c1)F)S(=O)(=O)N2CCOc3c2cccc3N4CCNCC4.Cl.Cl | 5-HT6 antagonist | 0.07 |
| MS 245 OXALATE | CN(C)CCC1=CN(C2=C1C=C(C=C2)OC)S(=O)(=O)C3=CC=CC=C3.C(=O)(C(=O)O)O | 5-HT6 antagonist | 0.09 |
| SB 399885 hydrochloride | COC1=C(C=C(C=C1)S(=O)(=O)NC2=C(C(=CC(=C2)Cl)Cl)OC)N3CCNCC3 | 5-HT6 antagonist | 0.16 |
| Ro 04-6790 | CNc1cc(NS(=O)(=O)c2ccc(N)cc2)nc(NC)n1 | 5-HT6 antagonist | 0.11 |
| WAY 208466 | CN(C)CCN1C=C(C2=C1N=CC=C2)S(=O)(=O)C3=CC=CC(=C3)F.Cl.Cl | 5-HT6 agonist | 0.21 |
| ST 1936 OXALATE | Cc1c(c2cc(ccc2[nH]1)Cl)CCN(C)C.C(=O)(C(=O)O)O | 5-HT6 agonist | 0.09 |
| EMD 386088 HYDROCHLORIDE | Cc1c(c2cc(ccc2[nH]1)Cl)C3=CCNCC3.Cl | 5-HT6 agonist | 0.15 |
| EMDT oxalate | CCc1c(c2cc(ccc2[nH]1)OC)CCN(C)C.C(=O)(C(=O)O)O | 5-HT6 agonist | -0.03 |

| Predicted Serotonin-6 | | | |
|---|---|---|---|
| 6028165 | COc1ccc(S(=O)(=O)NC2CCCC2)cc1Br | predicted 5-HT6 | 0.66 |
| 6366118 | COc1ccc(S(=O)(=O)N2CCC(C)CC2)cc1Br | predicted 5-HT6 | 0.66 |
| 6029941 | COc1ccc(OC)c(NS(=O)(=O)c2ccc(OC)c(Br)c2)c1 | predicted 5-HT6 | 0.61 |
| 6030057 | CCc1ccccc1NS(=O)(=O)c1ccc(OC)c(Br)c1 | predicted 5-HT6 | 0.36 |
| 6193422 | O=S(=O)(Nc1cccc(Cl)c1)c1ccc2c(c1)OCCO2 | predicted 5-HT6 | 0.46 |
| 6030006 | CCOc1ccccc1NS(=O)(=O)c1ccc(OC)c(Br)c1 | predicted 5-HT6 | 0.52 |
| 6013263 | COc1ccc(S(=O)(=O)Nc2ccc(C)cc2)cc1Br | predicted 5-HT6 | 0.61 |
| 5352629 | COc1ccccc1N1CCN(C(=O)c2ccc(C(=O)c3c(C)cc(C)cc3C)cc2)CC1 | predicted 5-HT6 | 0.48 |

| Other Serotonin Compounds Tested | | | |
|---|---|---|---|
| ALMOTRIPTAN MALATE | CN(C)CCc1c[nH]c2c1cc(cc2)CS(=O)(=O)N3CCCC3.C(C(=O)O)O)C(=O)O | 5-H1B/1D agonist | 0.18 |
| GR 55562 DIHYDROCHLORIDE | CN(C)CCCc1cc(ccc1O)C(=O)Nc2ccc(cc2)c3ccncc3.Cl.Cl | 5-HT1B silent antagonist | 0.24 |
| LISURIDE MALEATE | CCN(CC)C(=O)N[C@@H]1CN([C@@H]2Cc3c[nH]c4c3c(ccc4)C2=C1)C.C(=C\C(=O)O)\C(=O)O | 5-HT2b antagonist | 0.11 |
| MESULERGINE HYDROCHLORIDE | Cn1cc2c3c1cccc3[C@H]4C[C@@H](CN([C@@H]4C2)C)NS(=O)(=O)N(C)C.Cl | 5-HT2A/2C2C antagonist | 0.4 |
| Rizatriptan Benzoate | CN(C)CCc1c[nH]c2c1cc(cc2)Cn3cncn3.c1ccc(cc1)C(=O)O | 5-HT1B/1C agonist | -0.01 |
| Duloxetine Hydrochloride | CNCCC(c1cccs1)Oc2cccc3c2cccc3.Cl | SNRI | 0.27 |

**Supplementary Table 9. Continued**

| Other Serotonin Compounds Tested | SMILES | Mechanism Notes | Phenoscore |
|---|---|---|---|
| TCB-2 | COc1cc(c(c2c1C(C2)CN)OC)Br.Br | 5-HT2a agonist | 0.1 |
| Fluoxetine Hydrochloride | CNCCC(c1ccccc1)Oc2ccc(cc2)C(F)(F)F.Cl | SSRI | 0.46 |
| Ondansetron Hydrochloride | N2(c1c(cccc1)C3=C2CCC(C3=O)CN4C(=NC=C4)C)C | 5-HT3 antagonist | 0.13 |
| SB-216641 | Cc1cc(ccc1c2ccc(cc2)C(=O)Nc3ccc(c(c3)OCCN(C)C)OC)c4nc(on4)C | 5-HT1B antagonist | 0.11 |
| SDZ-205557 | CCN(CC)CCOC(=O)c1cc(c(cc1OC)N)Cl | 5-HT3/4 antagonist | 0.07 |
| sumatriptan | CNS(=O)(=O)Cc1ccc2c(c1)c(c[nH]2)CCN(C)C | 5-HT1 agonist | 0.07 |
| BW 723C86 | CC(N)Cc1c[nH]c2ccc(OCc3cccs3)cc12 | 5-HT2B agonist | 0.1 |
| DOI HYDROCHLORIDE | CC(Cc1cc(c(cc1OC)I)OC)N.Cl | 5-HT2A/2C agonist | 0.1 |
| Cisapride | Fc1ccc(cc1)OCCCN2CC(C(CC2)NC(=O)c3c(cc(c(c3)Cl)N)OC)OC | 5-HT4 agonist | 0.08 |
| Sertraline | ClC1=CC=C([C@H]2C3=C([C@H](CC2)NC)C=CC=C3)C=C1Cl | SSRI | 0.54 |
| Fluvoxamine maleate | FC(F)(F)c1ccc(cc1)/C(=N/OCCN)/CCCCOC | SSRI | 0.23 |
| Paroxetine Hydrochloride | Cl.Fc1ccc(cc1)[C@@H]1CCNC[C@H]1COc1ccc2OCOc2c1 | SSRI | 0.2 |
| 8-HYDROXY-DPAT | CCCN(CCC)C1CCc2cccc(O)c2C1 | 5-HT1A agonist | 0.49 |
| alpha-METHYLSEROTONIN | CC(Cc1c[nH]c2c1cc(cc2)O)N | 5-HT agonist | 0.18 |
| Trazodone hydrochloride | Cl.Clc1cccc(c1)N1CCN(CCCn2nc3ccccn3c2=O)CC1 | SSRI | 0.22 |
| 1-(3-CHLOROPHENYL)BIGUANIDE | c1cc(cc(c1)Cl)NC(=N)NC(=N)N.Cl | 5-HT3 agonist | 0.18 |
| Quipazine maleate salt | c1ccc2c(c1)ccc(n2)N3CCNCC3.C(=C\C(=O)O)\C(=O)O | 5-HT agonist | 0.16 |
| Zimelidine dihydrochloride monohydrate | Brc1ccc(cc1)/C(=C/CN(C)C)/c2cnccc2 | SSRI | 0.16 |
| Chlorpheniramine maleate | Clc1ccc(cc1)C(CCN(C)C)c2ncccc2 | SNRI | 0.05 |
| Buspirone hydrochloride | N1(CCN(CC1)c2ncccn2)CCCCN4C(=O)CC3(CCCC3)CC4=O | 5-HT1A agonist | 0.08 |
| FENFLURAMINE | CCNC(C)Cc1cccc(c1)C(F)(F)F.Cl | SSRI and 5-HT release stimulator | 0.13 |
| 57-DIHYDROXYTRYPTAMINE | c1c(cc(c2c1c(c[nH]2)CCN)O)O | 5-HT neurontoxn | 0.05 |
| serotonin | NCCc1c[nH]c2ccc(O)cc12 | 5-HT agonist | 0.19 |
| Desipramine hydrochloride | CNCCCN1c2ccccc2CCc3c1cccc3.Cl | SNRI | 0.49 |
| clomipramine | CN(C)CCCN1c2ccccc2CCc2ccc(Cl)cc12 | SNRI | 0.13 |
| Dihydroergotamine mesylate | CS(O)(=O)=O.[H][C@@]12CCCN1C(=O)[C@]([H])(Cc1ccccc1)N1C(=O)[C@](C)(NC(=O)<br>[C@@]3([H])CN(C)[C@]4([H])Cc5c[nH]c6cccc(c56)[C@@]4([H])C3)O[C@@]21O | 5-HT antagonist | 0.2 |
| PROPRANOLOL | CC(NCC(O)COC1=C(C=CC=C2)C2=CC=C1)C | 5-HT1/5-HT2 antagonist | 0.15 |
| clozapine | CN1CCN(CC1)C1=Nc2cc(Cl)ccc2Nc2ccccc12 | 5-HT2A/2C antagonist | 0.23 |
| ISAMOLTANE | CC(C)NCC(O)COc1ccccc1-n1cccc1 | 5-HT1B antagonist | 0.15 |
| METHIOTHEPIN | OC(=O)\C=C/C(O)=O.CSc1ccc2Sc3ccccc3CC(N3CCN(C)CC3)c2c1 | 5-HT2/1 antagonist | 0.33 |

**Supplementary Table 10. Isoflavone analogs of hit 7013338**

| Cpnd | C2 | C2' | C3' | C6' | C6 | C7 | phenocopy |
|---|---|---|---|---|---|---|---|
| 7013338 | $CH_3$ | Cl | H | H | $C_2H_5$ | $CH_3$ | positive |
| JG-17 | $CH_3$ | H | $CF_3O$ | Cl | $C_3H_7$ | H | negative |
| JG-41 | $C_3H_7$ | H | $CF_3O$ | H | $C_3H_7$ | H | negative |
| JG-30 | $C_2H_5$ | H | H | Cl | $C_8H_9$ | $C_3H_5O$ | negative |
| JG-13 | $C_2H_5$ | H | $CF_3O$ | H | $C_3H_7$ | $C_3H_5O$ | negative |
| JG-31 | $C_3H_7$ | H | H | Cl | $C_8H_9$ | $C_4H_7O$ | negative |
| JG-48 | $C_2H_5$ | H | $CF_3O$ | H | $C_8H_9$ | $C_3H_5O_2$ | negative |
| JG-47 | $C_3H_7$ | H | H | Cl | $C_8H_9$ | $C_3H_5O_2$ | negative |
| JG-46 | $C_2H_5$ | H | H | Cl | $C_8H_9$ | $C_3H_5O_2$ | negative |
| JG-34 | $C_2H_5$ | H | $CF_3O$ | H | $C_8H_9$ | $C_3H_5O$ | negative |
| JG-49 | $C_3H_7$ | H | $CF_3O$ | H | $C_8H_9$ | $C_3H_5O_2$ | negative |
| JG-35 | $C_3H_7$ | H | $CF_3O$ | H | $C_8H_9$ | $C_4H_7O$ | negative |
| JG-39 | $C_3H_7$ | H | H | Cl | $C_8H_9$ | H | negative |
| JG-43 | $C_3H_7$ | H | $CF_3O$ | H | $C_8H_9$ | H | negative |
| JG-42 | $C_2H_5$ | H | $CF_3O$ | H | $C_8H_9$ | H | negative |
| JG-38 | $C_2H_5$ | H | H | Cl | $C_8H_9$ | H | negative |
| JG-37 | $C_3H_7$ | H | H | Cl | $C_3H_7$ | H | positive |
| JG-29 | $C_3H_7$ | H | H | Cl | $C_3H_7$ | $C_4H_7O$ | positive |
| JG-44 | $C_2H_5$ | H | H | Cl | $C_3H_7$ | $C_3H_5O_2$ | positive |
| JG-16 | $C_2H_5$ | H | H | Cl | $C_3H_7$ | $C_3H_5O$ | positive |
| JG-45 | $C_3H_7$ | H | H | Cl | $C_3H_7$ | $C_3H_5O_2$ | positive |
| JG-18 | $C_2H_5$ | H | H | Cl | $C_3H_7$ | H | positive |

**Spectral Analysis of Isoflavone Analogs**

JG-13

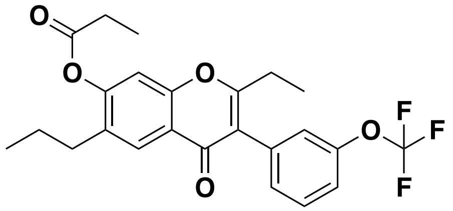


2-ethyl-4-oxo-6-propyl-3-(3-(trifluoromethoxy)phenyl)-4H-chromen-7-yl propionate: $^{1}$H-NMR (600 MHz, chloroform-d) δ = 8.08 (s, 1 H), 7.47-7.45 (m, 1H), 7.27 (s, 1 H), 7.25-7.23 (d, 1 H), 7.23-7.21 (d, 1 H), 7.15 (s, 1 H), 7.27-7.27 (m, 2 H), 2.61-2.59 (m, 2 H), 2.57-2.55 (m, 2 H), 1.67-1.61 (m, 2 H), 1.35-1.32 (m, 3 H), 1.27-1.24 (m, 3 H), 0.97-0.94 (m, 3 H); $^{13}$C-NMR (600 MHz, chloroform-d) δ = 176.31, 172.39, 167.95, 154.80, 153.18, 149.37, 135.19, 132.67, 129.91, 129.13, 127.43, 123.21, 121.87, 121.24, 120.42, 111.49, 32.09, 27.97, 26.32, 23.22, 14.00, 12.00, 9.29; HRMS (m/z): $[M+H]^+$ calcd., 448.1498; found, 449.1568.

JG-16

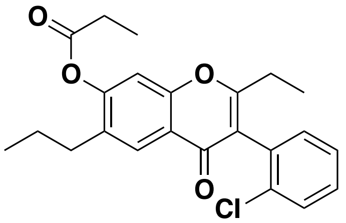


3-(2-chlorophenyl)-2-ethyl-4-oxo-6-propyl-4*H*-chromen-7-yl propionate: $^{1}$H-NMR (600 MHz, chloroform-d) δ = 8.09 (s, 1 H), 7.51-7.49 (m, 1 H), 7.36-7.31 (m, 2 H), 7.26 (s, 1 H), 7.23-7.22 (m, 1 H), 2.70-2.66 (m, 2 H), 2.60-2.59 (m, 2 H), 2.53-2.40 (m, 2 H), 1.67-1.61 (m, 2 H), 1.34-1.32 (m, 3 H), 1.23-1.20 (m, 3 H), 0.96-0.94 (m, 3 H); $^{13}$C-NMR (600 MHz, chloroform-d) δ = 175.95, 172.44, 168.20, 154.93, 153.06, 135.00, 132.54, 132.31, 129.89, 129.72, 127.53, 127.11, 121.29, 121.00, 111.50, 32.12, 27.99, 26.35, 23.25, 14.06, 11.43, 9.33; HRMS (m/z): $[M+H]^+$ calcd., 398.1285; found, 399.1349.

JG-17

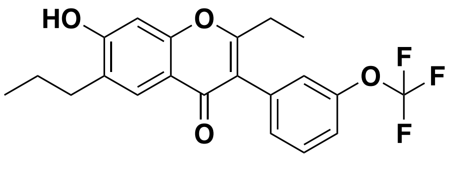


2-ethyl-7-hydroxy-6-propyl-3-(3-(trifluoromethoxy)phenyl)-4*H*-chromen-4-one: $^{1}$H-NMR (600 MHz, chloroform-d) δ = 7.94 (s, 1 H0, 7.42-7.39 (m, 1 H), 7.23-7.22 (d, 1 H), 7.17-7.11 (m, 2 H), 6.78 (m, 1 H), 3.66 (s, 1 H), 2.63-2.60 (m, 2 H), 2.56-2.52 (m, 2 H), 1.66-1.62 (m, 2 H), 1.25-1.22 (m, 3 H), 0.96-0.94 (m, 3 H); $^{13}$C-NMR (600 MHz, chloroform-d) δ = 177.38, 167.95, 160.40, 156.44, 135.35, 130.00, 129.20, 129.01, 126.84, 123.22,

122.22, 121.35, 120.35, 119.93, 116.24, 102.25, 31.82, 26.33, 22.68, 14.05, 12.04; HRMS (m/z): $[M+H]^+$ calcd., 392.1235; found, 393.1311.

JG-18

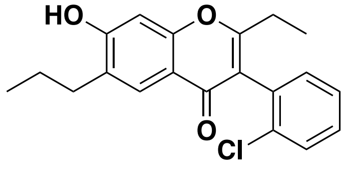

3-(2-chlorophenyl)-2-ethyl-7-hydroxy-6-propyl-4*H*-chromen-4-one: $^{1}$H-NMR (600 MHz, chloroform-d) δ = 8.57 (s, 1 H), 7.94 (s, 1 H), 7.43-7.41 (d, 1 H), 7.29-7.24 (m, 3 H), 6.80 (s, 1 H), 2.62-2.59 (m, 2 H), 2.52-2.39 (m, 2 H), 1.67-1.61 (m, 2 H), 1.23-1.20 (m, 3 H), 0.96-0.94 (m, 3 H); $^{13}$C-NMR (600 MHz, chloroform-d) δ = 177.09, 168.02, 160.08, 156.56, 135.08, 132.67, 132.39, 129.75, 129.60, 129.11, 127.04, 126.57, 120.23, 115.91, 102.28, 31.89, 26.33, 22.68, 14.12, 11.48; HRMS (m/z): $[M+H]^+$ calcd., 342.1023; found, 343.1103.

JG-29

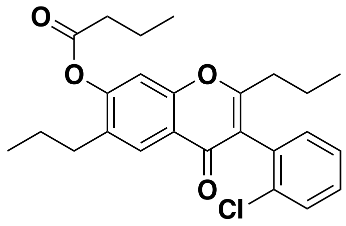

3-(2-chlorophenyl)-4-oxo-2,6-dipropyl-4*H*-chromen-7-yl butyrate: $^{1}$H-NMR (600 MHz, chloroform-d) δ = 8.09 (s, 1 H), 7.50-7.48 (m, 1 H), 7.35-7.31 (m, 2 H), 7.24 (s, 1 H), 7.23-7.21 (m, 1 H), 2.63-2.61 (m, 2 H), 2.60-2.58 (m, 2 H), 2.49-2.35 (m, 2 H), 1.86-1.81 (m, 2 H), 1.73-1.68 (m, 2 H), 1.67-1.61 (m, 2 H), 1.10-1.08 (m, 3 H), 0.97-0.94 (m, 3 H), 0.91-0.88 (m, 3 H); $^{13}$C-NMR (600 MHz, chloroform-d) δ = 175.88, 171.64, 167.24, 154.87, 153.06, 135.03, 132.56, 132.41, 129.86, 129.70, 127.51, 127.05, 121.62, 121.25, 111.48, 36.40, 34.64, 32.13, 23.27, 20.39, 18.63, 14.07, 13.96, 13.88; HRMS (m/z): $[M+H]^+$ calcd., 426.1598; found, 427.1698.

JG-30

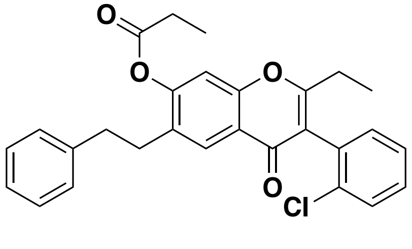

3-(2-chlorophenyl)-2-ethyl-4-oxo-6-phenethyl-4H-chromen-7-yl propionate: 1H-NMR (600 MHz, chloroform-d) δ = 8.14 (s, 1 H), 7.52-7.50 (m, 1H), 7.36-7.34 (m, 2 H), 7.31-7.28 (m, 3 H), 7.24-7.18 (m, 4 H), 2.92 (m, 4 H), 2.68-2.64 (m, 2 H), 2.55-2.41 (m, 2 H),

1.33-1.30 (m, 3 H), 1.24-1.21 (m, 3 H); 13C-NMR (600 MHz, chloroform-d) δ = 175.86, 172.33, 168.28, 155.06, 152.97, 141.30, 134.99, 132.48, 132.39, 131.72, 129.89, 129.76, 128.68, 128.49, 127.56, 127.13, 126.37, 121.33, 121.03, 111.56, 36.59, 32.18, 27.92, 26.36, 11.43, 9.28; HRMS (m/z): [M+H]+ calcd., 460.1441; found, 461.1531.

JG-31

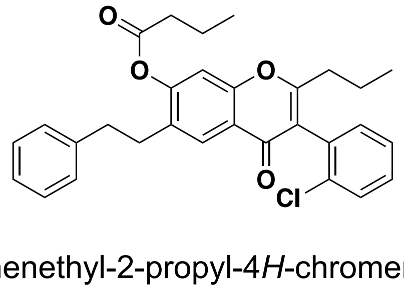


3-(2-chlorophenyl)-4-oxo-6-phenethyl-2-propyl-4*H*-chromen-7-yl butyrate: $^{1}$H-NMR (600 MHz, chloroform-d) δ = 8.15 (s, 1 H), 7.52-7.50 (m, 1 H), 7.35-7.34 (m, 2 H), 7.31-7.28 (m, 3 H), 7.24-7.19 (m, 4 H), 2.92-2.91 (m, 4 H), 2.63-2.60 (m, 2 H), 2.52-2.36 (m, 2 H), 1.86-1.77 (m, 2 H), 1.76-1.65 (m, 2 H), 1.09-1.07 (m, 3 H), 0.92-0.90 (m, 3 H); $^{13}$C-NMR (600 MHz, chloroform-d) δ = 175.79, 171.53, 167.34, 155.01, 152.98, 141.31, 135.02, 132.50, 132.39, 131.17, 129.88, 129.73, 128.48, 127.56, 127.07, 126.36, 121.66, 121.31, 111.55, 36.55, 34.65, 32.18, 20.40, 18.59, 13.98, 13.88; HRMS (m/z): $[M+H]^+$ calcd., 488.1754; found, 489.1849.

JG-34

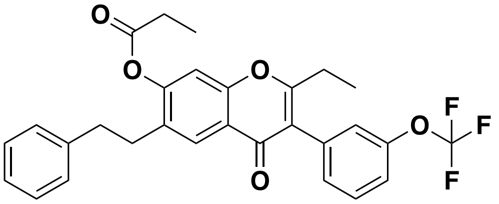


2-ethyl-4-oxo-6-phenethyl-3-(3-(trifluoromethoxy)phenyl)-4*H*-chromen-7-yl propionate: $^{1}$H-NMR (600 MHz, chloroform-d) δ = 8.11 (s, 1 H), 7.48-7.46 (m, 1 H), 7.30-7.26 (m, 3 H), 7.23-7.21 (m, 3 H), 7.18-7.15 (m, 3 H), 2.92 (m, 4 H), 2.68-2.64 (m, 2 H), 2.59-2.55 (m, 2 H), 1.32-1.30 (m, 3 H), 1.27-1.24 (m, 3 H); $^{13}$C-NMR (600 MHz, chloroform-d) δ = 176.22, 172.29, 168.02, 154.49, 153.10, 149.38, 141.21, 135.13, 131.83, 129.95, 129.13, 128.70, 128.51, 127.51, 126.40, 123.21, 121.91, 121.30, 120.47, 111.55, 36.56, 32.15, 27.93, 26.35, 12.01, 9.27; HRMS (m/z): $[M+H]^+$ calcd., 510.1654; found, 511.1745.

JG-35

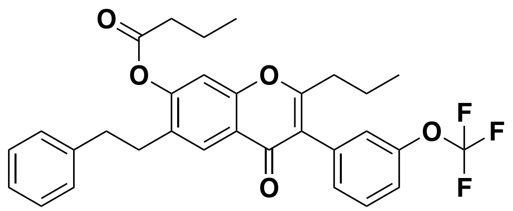

4-oxo-6-phenethyl-2-propyl-3-(3-(trifluoromethoxy)phenyl)-4*H*-chromen-7-yl butyrate: $^{1}$H-NMR (600 MHz, chloroform-d) δ = 8.11 (s, 1 H), 7.48-7.46 (m, 1 H), 7.30-7.28 (m, 2 H), 7.27 (s, 1 H), 7.25-7.24 (d, 1 H), 7.22-7.21 (d, 2 H), 7.18-7.17 (d, 2 H), 7.14 (s, 1 H), 2.92 (m, 4 H), 2.62-2.60 (m, 2 H), 2.53-2.51 (m, 2 H), 1.84-1.81 (m, 2 H), 1.75-1.71 (m, 2 H), 1.09-1.06 (m, 3 H), 0.93-0.90 (m, 3 H); $^{13}$C-NMR (600 MHz, chloroform-d) δ = 176.19, 171.49, 167.01, 154.89, 153.11, 149.36, 141.21, 131.84, 129.92, 128.68, 128.50, 127.50, 126.39, 123.31, 122.56, 121.28, 120.48, 111.55, 46.52, 36.34, 34.57, 32.15, 20.98, 18.58, 13.89, 13.85; HRMS (m/z): $[M+H]^+$ calcd., 538.1967; found, 539.2066.

JG-037

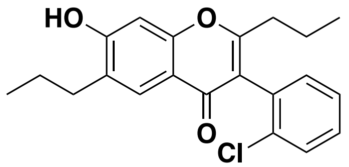

3-(2-chlorophenyl)-7-hydroxy-2,6-dipropyl-4*H*-chromen-4-one: $^{1}$H-NMR (600 MHz, chloroform-d) δ = 8.76 (s, 1 H), 7.94, (s, 1 H), 7.42-7.40 (d, 1 H), 7.27-7.24 (m, 3 H), 6.80 (s, 1 H), 2.61-2.59 (m, 2 H), 2.50-2.35 (m, 2 H), 1.75-1.68 (m, 2 H), 1.68-1.60 (m, 2 H), 0.96-0.94 (m, 3 H), 0.91 (m, 3 H); $^{13}$C-NMR (600 MHz, chloroform-d) δ = 177.16, 167.20, 160.99, 156.60, 135.17, 132.71, 132.55, 129.77, 129.61, 129.26, 127.03, 126.53, 120.89, 115.83, 102.33, 34.67, 31.96, 22.70, 20.48, 14.17, 13.98; HRMS (m/z): $[M+H]^+$ calcd., 356.1179; found, 357.1243.

JG-38

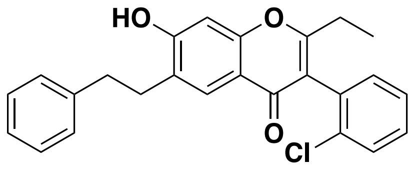

3-(2-chlorophenyl)-2-ethyl-7-hydroxy-6-phenethyl-4*H*-chromen-4-one: $^{1}$H-NMR (600 MHz, chloroform-d) δ = 8.01 (s, 1 H), 7.45-7.43 (d, 1 H), 7.29-7.24 (m, 6 H), 7.22-7.19 (m, 2 H), 6.82 (s, 1 H), 2.94-2.93 (m, 4 H), 2.51-2.40 (m, 2 H), 1.25-1.20 (m, 3 H); $^{13}$C-NMR (600 MHz, chloroform-d) δ = 176.78, 168.03, 160.12, 156.61, 141.95, 135.10, 132.64, 132.39, 129.84, 129.71, 128.67, 128.60, 127.12, 127.07, 126.24, 120.47, 116.55, 102.63, 36.17, 32.32, 26.37, 11.54; HRMS (m/z): $[M+H]^+$ calcd., 404.1179; found, 405.1255.

JG-39

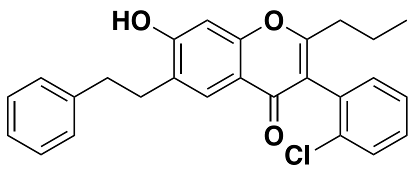

3-(2-chlorophenyl)-7-hydroxy-6-phenethyl-2-propyl-4*H*-chromen-4-one: $^{1}$H-NMR (600 MHz, chloroform-d) δ = 8.80 (s, 1 H), 8.01 (s, 1 H), 7.41-7.40 (d, 1 H), 7.26-7.25 (m, 4 H), 7.22-7.21 (m, 3 H), 7.19-7.16 (m, 1 H), 6.83 (s, 1 H), 2.94-2.91 (m, 4 H), 2.50-2.35 (m, 2 H), 1.75-1.66 (m, 2 H), 0.91-0.88 (m, 3 H); $^{13}$C-NMR (600 MHz, chloroform-d) δ = 177.14, 167.35, 161.06, 156.77,, 142.15, 135.19, 132.63, 132.53, 129.08, 129.68, 128,68, 128,59, 127.06, 126.62, 126.11, 10.92, 115.91, 102.51, 36.07, 34.69, 32.44, 20.51, 13.98; HRMS (m/z): $[M+H]^+$ calcd., 418.1336; found, 419.1402.

JG-41

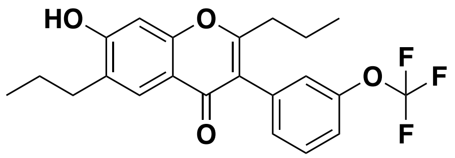

7-hydroxy-2,6-dipropyl-3-(3-(trifluoromethoxy)phenyl)-4*H*-chromen-4-one: $^{1}$H-NMR (600 MHz, chloroform-d) δ = 8.28 (s, 1 H), 7.94 (s, 1 H), 7.42-7.39 (m, 1 H), 7.23-7.22 (m, 1 H), 7.17-7.15 (m, 2 H), 6.77 (s, 1 H), 2.63-2.60 (m, 2 H), 2.51-2.48 (m, 2 H), 1.74-1.66 (m, 2 H), 1.66-1.61 (m, 2 H), 0.96-0.94 (m, 3 H), 0.91-0.90 (m, 3 H); $^{13}$C-NMR (600 MHz, chloroform-d) δ = 177.40, 166.01, 160.67, 156.44, 149.38, 135.47, 129.91, 129.33, 129.19, 126.77, 123.34, 121.96, 120.33, 116.08, 102.20, 34.57, 31.86, 22.67, 21.04, 14.06, 13.80; HRMS (m/z): $[M+H]^+$ calcd., 406.1392; found, 407.1482.

JG-42

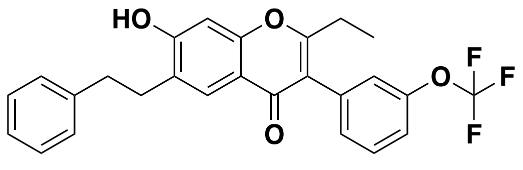

2-ethyl-7-hydroxy-6-phenethyl-3-(3-(trifluoromethoxy)phenyl)-4*H*-chromen-4-one: $^{1}$H-NMR (600 MHz, chloroform-d) δ = 7.98 (s, 1 H), 7.28-7.25 (m, 3 H), 7.23-7.22 (d, 2 H), 7.19-7.16 (m, 4 H), 6.79 (s, 1 H), 3.67 (s, 2 H), 2.95-2.91 (m, 4 H), 2.55-2.51 (m, 2 H), 1.24-1.22 (m, 3 H); $^{13}$C-NMR (600 MHz, chloroform-d) δ = 177.24, 175.89, 170.00, 160.29, 156.53, 141.79, 130.10, 129.97, 129.73, 129.21, 128.65, 128.58, 120.01,

127.03, 126.26, 123.24, 122.22, 121.41, 120.40, 119.97, 102.49, 40.65, 36.06, 32.17, 26.33, 12.04; HRMS (m/z): $[M+H]^+$ calcd., 454.1392; found, 455.1460

JG-43

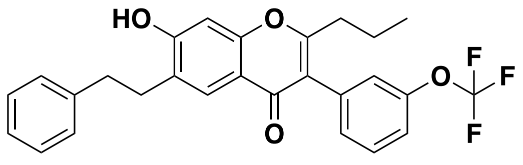

7-hydroxy-6-phenethyl-2-propyl-3-(3-(trifluoromethoxy)phenyl)-4*H*-chromen-4-one: $^1$H-NMR (600 MHz, chloroform-d) δ = 8.00 (s, 1 H), 7.41-7.39 (m, 1 H), 7.26-7.22 (m, 3 H), 7.19-7.14 (m, 6 H), 6.79 (s, 1 H), 2.95-2.90 (m, 4 H), 2.50-2.48 (m, 2 H), 1.74-1.67 (m, 2 H), 0.91-0.89 (m, 3 H); $^{13}$C-NMR (600 MHz, chloroform-d) δ = 177.53, 167.20, 160.95, 156.65, 149.39, 141.90, 135.46, 129.98, 129.33, 128.58, 126.72, 126.16, 123.34, 121.94, 120.41, 116.02, 102.36, 35.96, 34.59, 32.22, 21.05, 13.80; HRMS (m/z): $[M+H]^+$ calcd., 468.1458; found, 469.1593.

JG-44

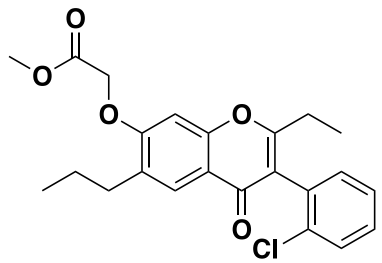

methyl 2-((3-(2-chlorophenyl)-2-ethyl-4-oxo-6-propyl-4*H*-chromen-7-yl)oxy)acetate: $^1$H-NMR (600 MHz, chloroform-d) δ = 7.98 (s, 1 H), 7.49-7.48 (m, 1 H), 7.33-7.31 (m, 2 H), 7.23-7.21 (m, 1 H), 6.99 (s, 1 H), 4.77 (s, 1 H), 3.84 (s, 1 H), 2.73-2.71 (m, 2 H0, 2.51-2.38 (m, 2 H), 1.71-1.65 (m, 2 H), 1.23-1.20 (m, 3 H), 0.98-0.95 (m, 3 H); $^{13}$C-NMR (600 MHz, chloroform-d) δ = 175.87, 168.72, 167.37, 160.28, 156.20, 134.99, 132.77, 132.34, 130.30, 129.81, 129.60, 127.10, 127.04, 120.91, 117.50, 98.83, 65.46, 52.63, 32.08, 29.86, 26.26, 22.87, 14.12, 11.54; HRMS (m/z): $[M+H]^+$ calcd., 414.1234; found, 415.1306.

JG-45

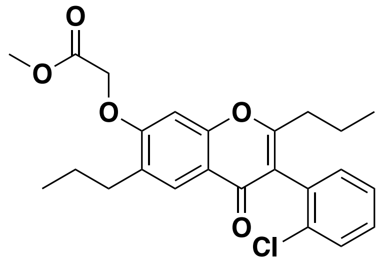


methyl 2-((3-(2-chlorophenyl)-4-oxo-2,6-dipropyl-4*H*-chromen-7-yl)oxy)acetate: $^{1}$H-NMR (600 MHz, chloroform-d) δ = 7.99 (s, 1 H), 7.49-7.48 (m, 1 H), 7.33-7.31 (m, 2 H), 7.22-7.21 (m, 1 H), 6.69 (s, 1 H), 4.77 (s. 2 H), 3.85 (s, 3 H), 2.73-2.71 (m, 2 H), 2.49-2.33 (m, 2 H), 1.72-1.66 (m, 4 H), 0.98-0.95 (m, 3 H), 0.91-0.88 (m, 3 H); $^{13}$C-NMR (600 MHz, chloroform-d) δ = 175.80, 168.74, 166.40, 160.28, 156.17, 135.04, 132.81, 132.46, 130.31, 129.83, 129.59, 127.13, 127.00, 121.58, 117.53, 98.83, 65.49, 52.65, 34.61, 32.10, 39.88, 22.91, 20.51, 14.16, 13.99; HRMS (m/z): $[M+H]^+$ calcd., 428.1392; found, 429.1467.

JG-46

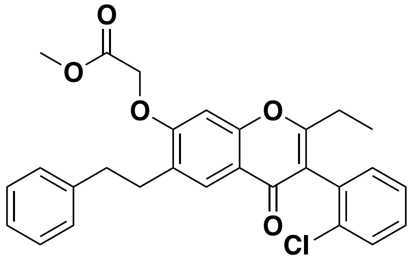


methyl 2-((3-(2-chlorophenyl)-2-ethyl-4-oxo-6-phenethyl-4*H*-chromen-7-yl)oxy)acetate: $^{1}$H-NMR (600 MHz, chloroform-d) δ = 8.03 (s, 1 H), 7.50-7.49 (m, 1 H), 7.34-7.33 (m, 2 H), 7.30-7.27 (m, 2 H), 7.26-7.24 (m, 3 H), 7.20-7.18 (m, 1 H), 6.72 (s, 1 H), 4.74 (s, 2 H), 3.86 (s, 3 H), 3.06-2.94 (m, 4 H), 2.53-2.39 (m, 2 H), 1.24-1.21 (m, 3 H); $^{13}$C-NMR (600 MHz, chloroform-d) δ = 175.81, 168.61, 167.46, 160.25, 156.40, 142.09, 135.02, 132.74, 132.35, 129.87, 129.66, 128.70, 128.50, 127.27, 127.09, 126.10, 121.00, 117.66, 99.05, 65.55, 52.68, 36.31, 32.51, 26.30, 11.57; HRMS (m/z): $[M+H]^+$ calcd., 476.1392; found, 477.1470

JG-047

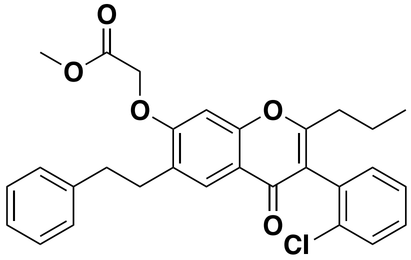


methyl 2-((3-(2-chlorophenyl)-4-oxo-6-phenethyl-2-propyl-4*H*-chromen-7-yl)oxy)acetate: $^{1}$H-NMR (600 MHz, chloroform-d) δ = 8.06 (s, 1 H0, 7.53-7.51 (d, 1 H), 7.36-7.35 (m, 2 H), 7.33-7.25 (m, 5 H), 7.23-7.20 (m, 1 H), 6.74 (s, 1 H), 4.76 (s, 2 H0, 3.89 (s, 3 H), 3.09-2.97 (m, 4 H), 2.53-2.37 (m, 2 H), 1.77-1.70 (m, 2 H), 0.94-0.92 (m, 3 H); $^{13}$C-NMR (600 MHz, chloroform-d) δ = 175.70, 168.56, 166.46, 160.20, 156.32, 142.05, 135.01,

132.71, 132.41, 129.82, 129.60, 129.52, 128.66, 128.46, 127.20, 127.00, 126.06, 121.59, 117.60, 99.00, 65.50, 52.63, 36.27, 34.59, 32.49, 20.48, 13.97; HRMS (m/z): $[M+H]^+$ calcd., 490.1547; found, 491.1632.

JG-48

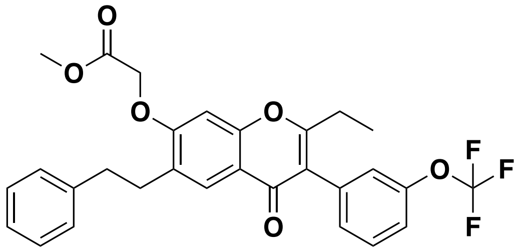


methyl 2-((2-ethyl-4-oxo-6-phenethyl-3-(3-(trifluoromethoxy)phenyl)-4*H*-chromen-7-yl)oxy)acetate: $^1$H-NMR (600 MHz, chloroform-d) δ = 8.02 (s, 1 H), 7.50-7.47 (m, 1 H), 7.32-7.29 (m, 3 H), 7.27-7.20 (m, 4 H), 7.17 (s, 1 H), 6.72 (s, 1 H), 4.75 (s, 2 H), 3.99 (s, 3 H), 3.09-2.96 (m, 4 H), 2.60-2.56 (m, 2 H), 1.30-1.27 (m, 3 H); $^{13}$C-NMR (600 MHz, chloroform-d) δ = 176.12, 168.55, 167.20, 160.36, 156.27, 149.36, 141.97, 135.38, 129.87, 129.68, 129.18, 128.71, 128.50, 127.21, 126.12, 123.23, 121.88, 120.36, 117.63, 98.98, 65.56, 52.68, 36.27, 32.44, 26.26, 12.14; HRMS (m/z): $[M+H]^+$ calcd., 526.1603; found, 527.1692.

JG-49

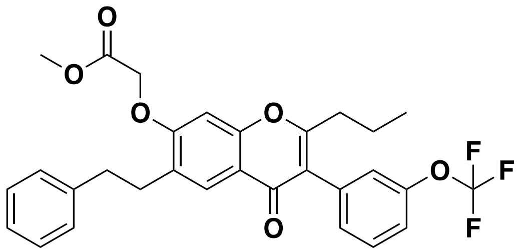


methyl 2-((4-oxo-6-phenethyl-2-propyl-3-(3-(trifluoromethoxy)phenyl)-4*H*-chromen-7-yl)oxy)acetate: $^1$H-NMR (600 MHz, chloroform-d) δ = 8.03 (s, 1 H), 7.50-7.47 (m, 1 H), 7.32-7.20 (m, 7 H), 7.16 (s, 1 H), 6.71 (s, 1 H), 4.75 (s, 2 H), 3.89 (s, 3 H), 3.09-3.07 (2.97 (m, 4 H), 2.54-2.52 (m, 2 H), 1.79-1.72 (m, 2 H), 0.96-0.93 (m, 3 H); $^{13}$C-NMR (600 MHz, chloroform-d) δ = 176.09, 168.54, 166.16, 160.35, 156.23, 149.33, 141.98, 135.45, 129.85, 129.68, 129.30, 128.70, 128.50, 127.20, 126.11, 123.33, 122.52, 120.37, 117.63, 98.98, 65.55, 52.67, 36.27, 34.53, 32.50, 29.89, 21.09, 13.87; HRMS (m/z): $[M+H]^+$ calcd., 540.1760; found, 541.1815.

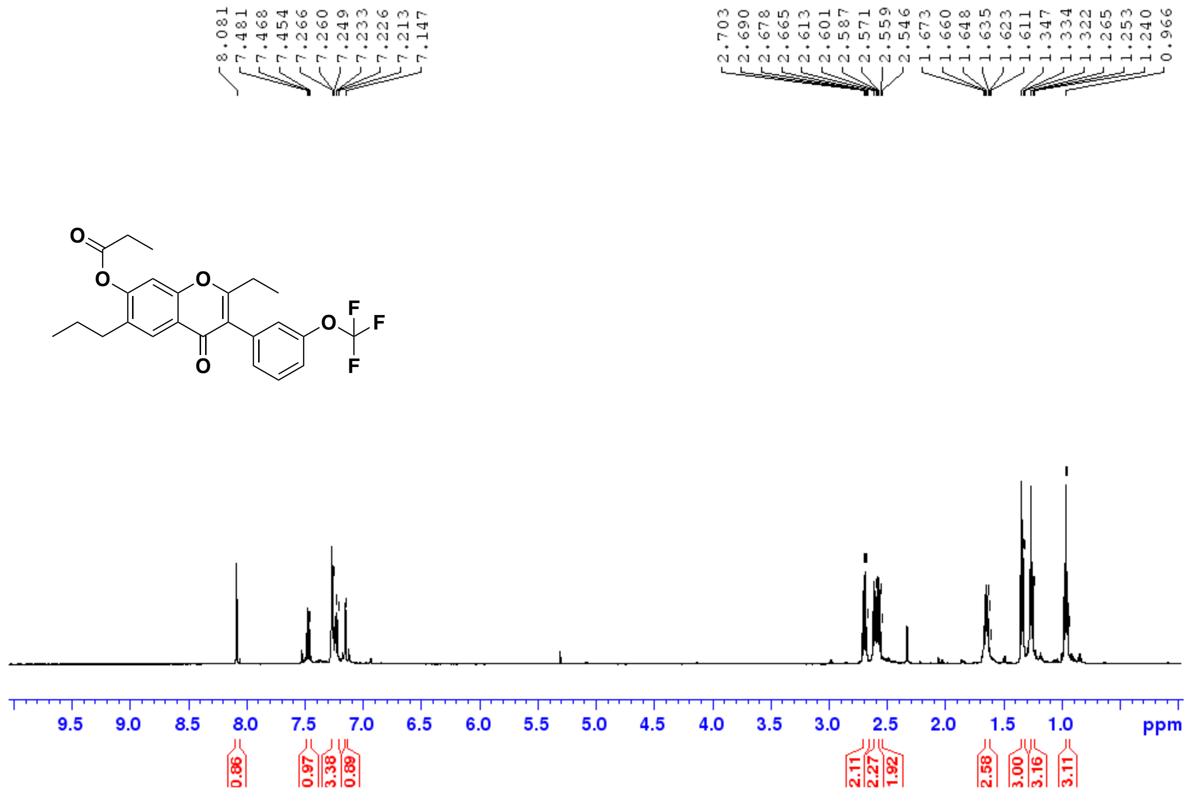

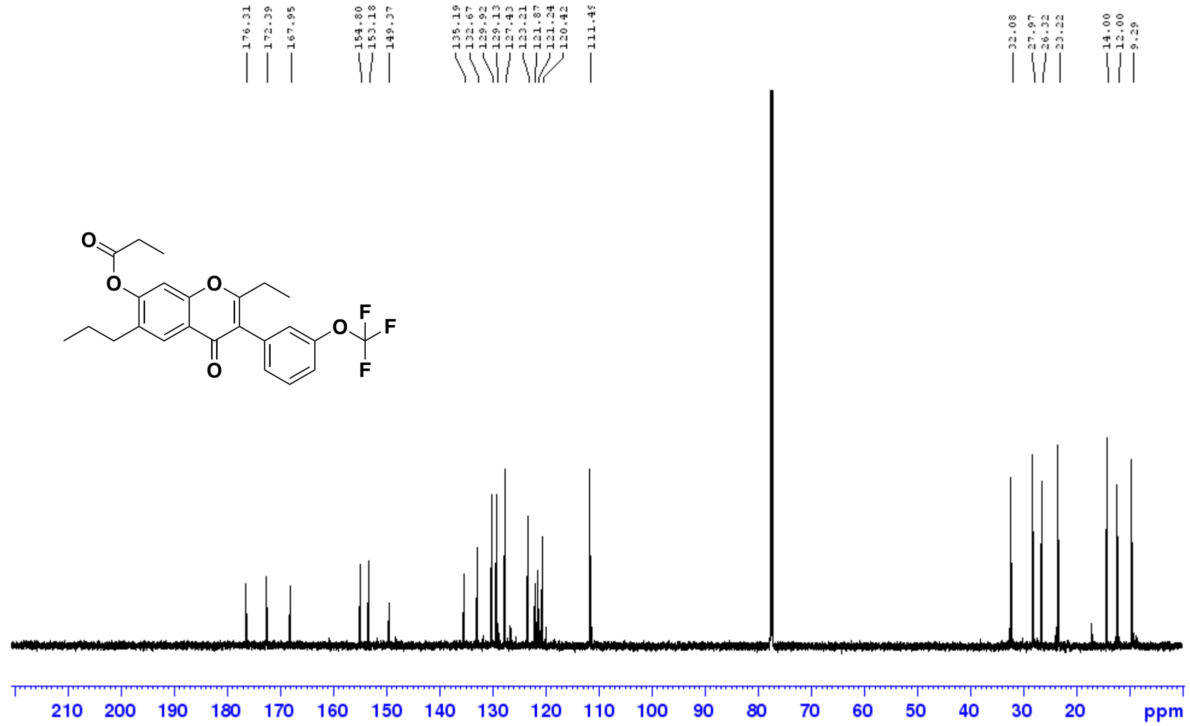

JG-16

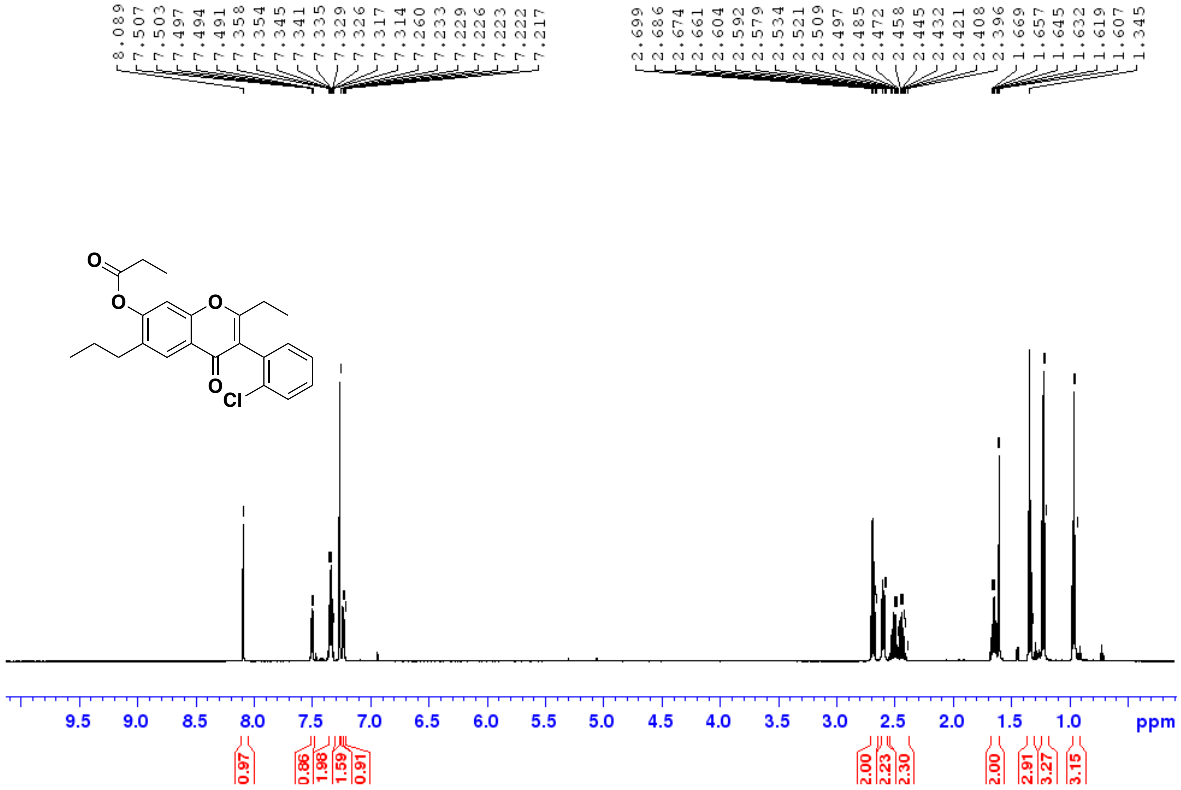
8.089 7.507 7.503 7.497 7.494 7.491 7.358 7.354 7.345 7.341 7.335 7.329 7.326 7.317 7.314 7.260 7.233 7.229 7.226 7.223 7.222 7.217
2.699 2.686 2.674 2.661 2.604 2.592 2.579 2.534 2.521 2.509 2.497 2.485 2.472 2.458 2.445 2.432 2.421 2.408 2.396 1.669 1.657 1.645 1.632 1.619 1.607 1.345
O
O
O
O
Cl
9.5 9.0 8.5 8.0 7.5 7.0 6.5 6.0 5.5 5.0 4.5 4.0 3.5 3.0 2.5 2.0 1.5 1.0 ppm
0.97 0.86 1.98 1.59 0.91 2.00 2.23 2.30 2.00 2.91 3.27 3.15

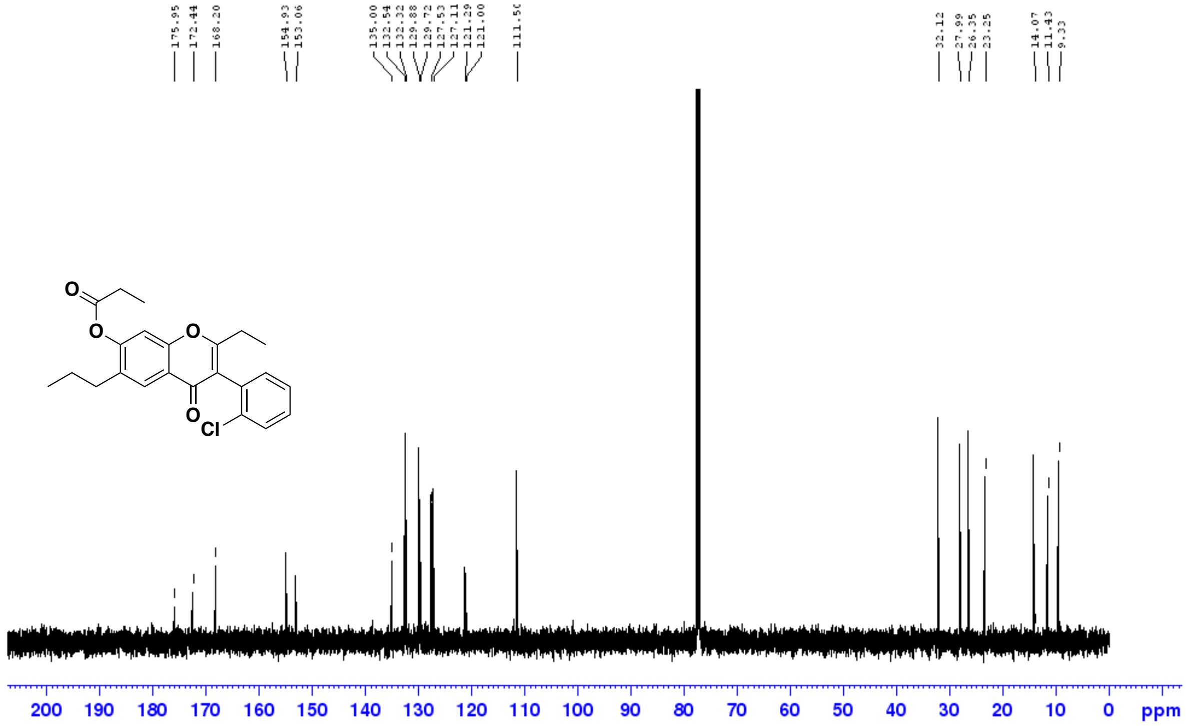
175.95 172.44 168.20 154.93 153.06 135.00 132.54 132.32 129.88 129.72 127.53 127.11 121.29 121.00
32.12 27.99 26.35 23.25 14.07 11.43 9.33
O
O
O
O
Cl
200 190 180 170 160 150 140 130 120 110 100 90 80 70 60 50 40 30 20 10 0 ppm

JG-17

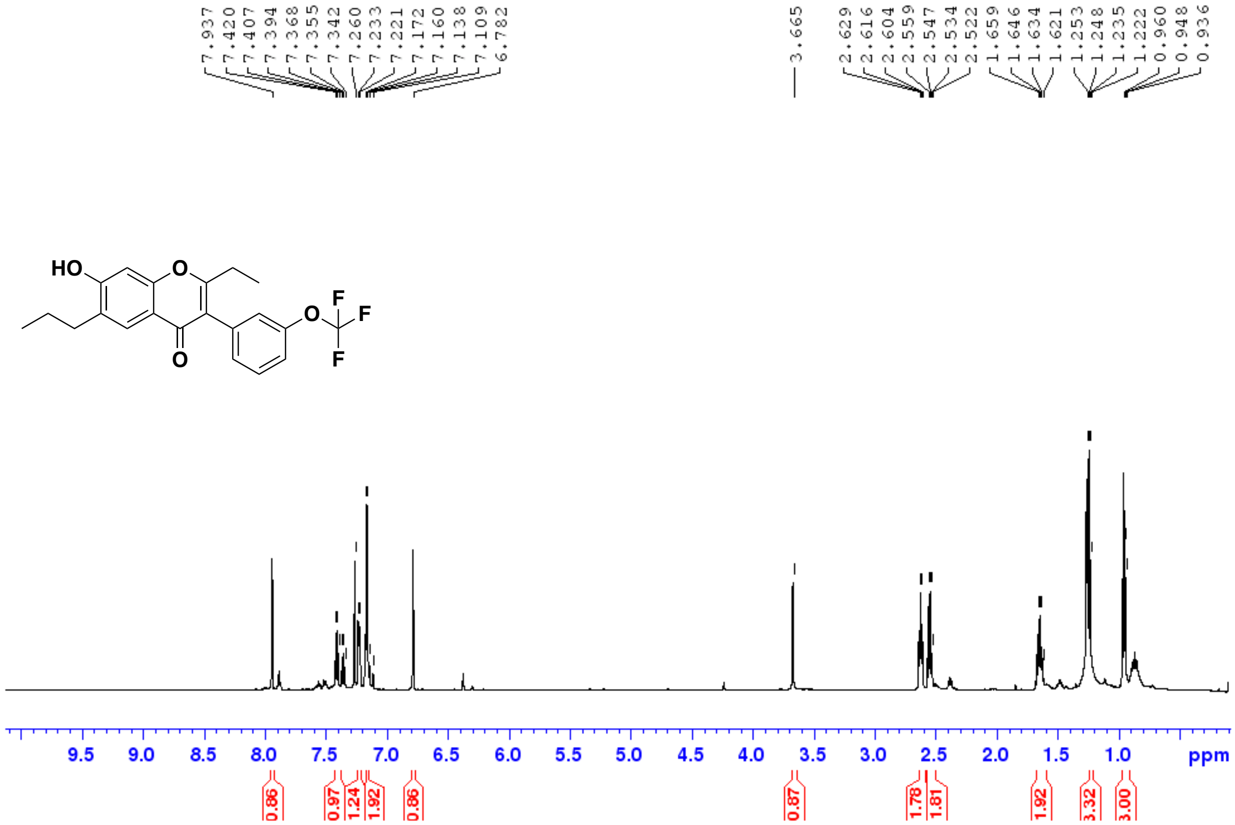
7.937
7.420
7.407
7.394
7.368
7.355
7.342
7.260
7.233
7.221
7.172
7.160
7.138
7.109
6.782
3.665
2.629
2.616
2.604
2.559
2.547
2.534
2.522
1.659
1.646
1.634
1.621
1.253
1.248
1.235
1.222
0.960
0.948
0.936
HO
O
O
O
F
F
F
9.5 9.0 8.5 8.0 7.5 7.0 6.5 6.0 5.5 5.0 4.5 4.0 3.5 3.0 2.5 2.0 1.5 1.0 ppm
0.86
0.97
1.24
1.92
0.86
0.87
1.78
1.81
1.92
3.32
3.00

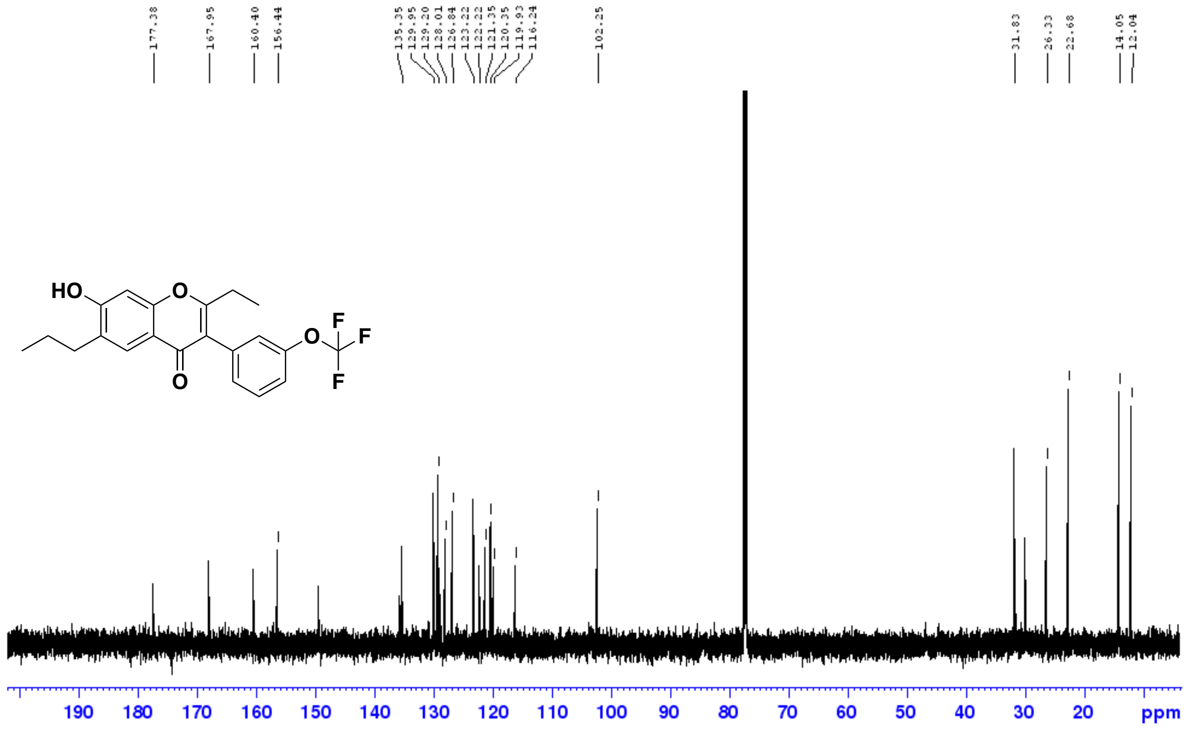
177.38
167.95
160.40
156.44
135.35
129.95
129.20
128.01
126.84
123.22
122.22
121.35
120.35
119.93
116.24
102.25
31.83
26.33
22.68
14.05
12.04
HO
O
O
O
F
F
F
190 180 170 160 150 140 130 120 110 100 90 80 70 60 50 40 30 20 ppm

JG-18

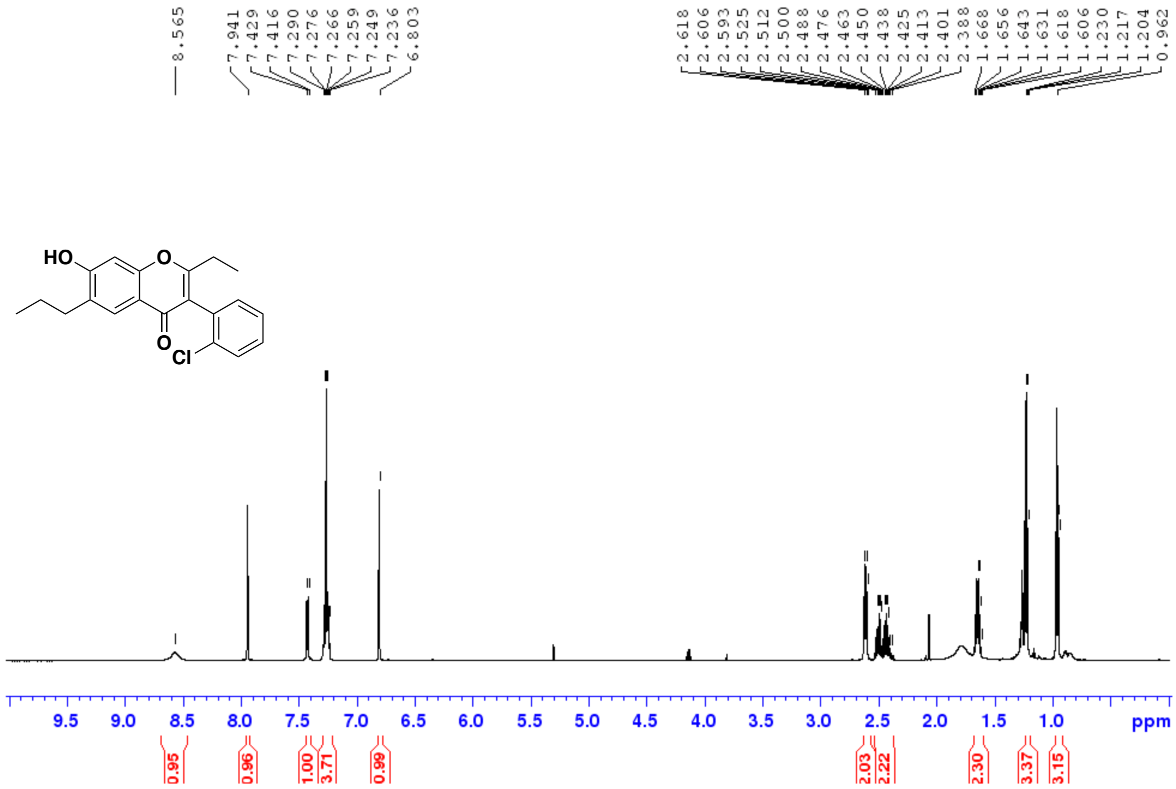
8.565
7.941
7.429
7.416
7.290
7.276
7.266
7.259
7.249
7.236
6.803
2.618
2.606
2.593
2.525
2.512
2.500
2.488
2.476
2.463
2.450
2.438
2.425
2.413
2.401
2.388
1.668
1.656
1.643
1.631
1.618
1.606
1.230
1.217
1.204
0.962
HO
O
O
Cl
9.5
9.0
8.5
8.0
7.5
7.0
6.5
6.0
5.5
5.0
4.5
4.0
3.5
3.0
2.5
2.0
1.5
1.0
ppm
0.95
0.96
1.00
3.71
0.99
2.03
2.22
2.30
3.37
3.15

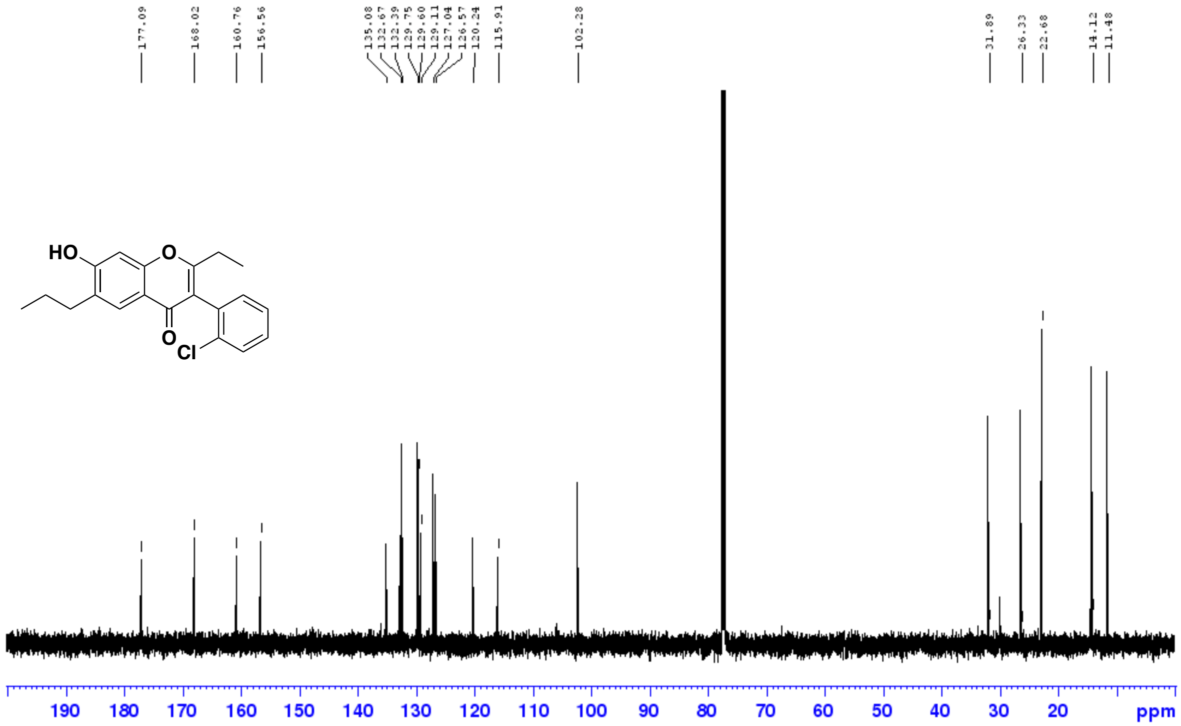
177.09
168.02
160.76
156.56
135.08
132.67
132.39
129.75
129.60
129.11
127.04
126.57
120.24
115.91
102.28
31.89
26.33
22.68
14.12
11.48
HO
O
O
Cl
190
180
170
160
150
140
130
120
110
100
90
80
70
60
50
40
30
20
ppm

JG-29

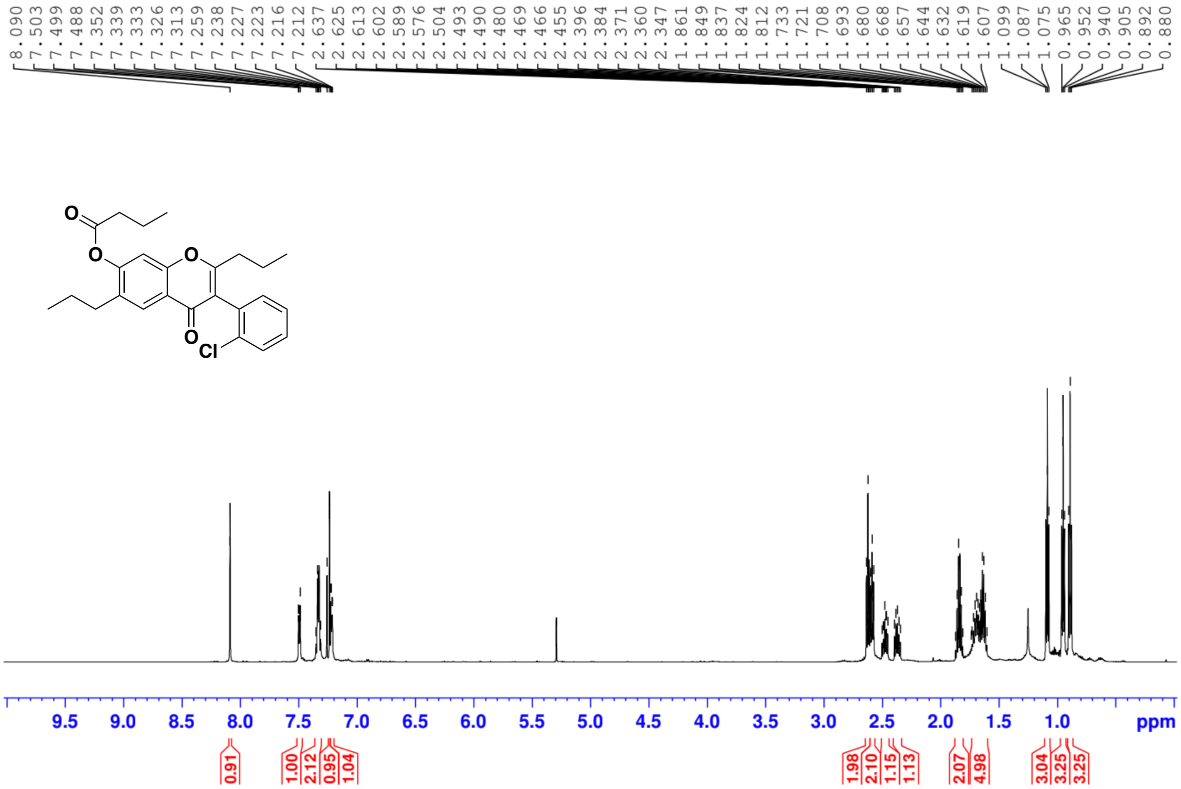
8.090
7.503
7.499
7.488
7.352
7.339
7.333
7.326
7.313
7.259
7.238
7.227
7.223
7.216
7.212
2.637
2.625
2.613
2.602
2.589
2.576
2.504
2.493
2.490
2.480
2.469
2.466
2.455
2.396
2.384
2.371
2.360
2.347
1.861
1.849
1.837
1.824
1.812
1.733
1.721
1.708
1.693
1.680
1.668
1.657
1.644
1.632
1.619
1.607
1.099
1.087
1.075
0.965
0.952
0.940
0.905
0.892
0.880
O
O
O
O
Cl
9.5 9.0 8.5 8.0 7.5 7.0 6.5 6.0 5.5 5.0 4.5 4.0 3.5 3.0 2.5 2.0 1.5 1.0 ppm
0.91
1.00
2.12
0.95
1.04
1.98
2.10
1.15
1.13
2.07
4.98
3.04
3.25
3.25

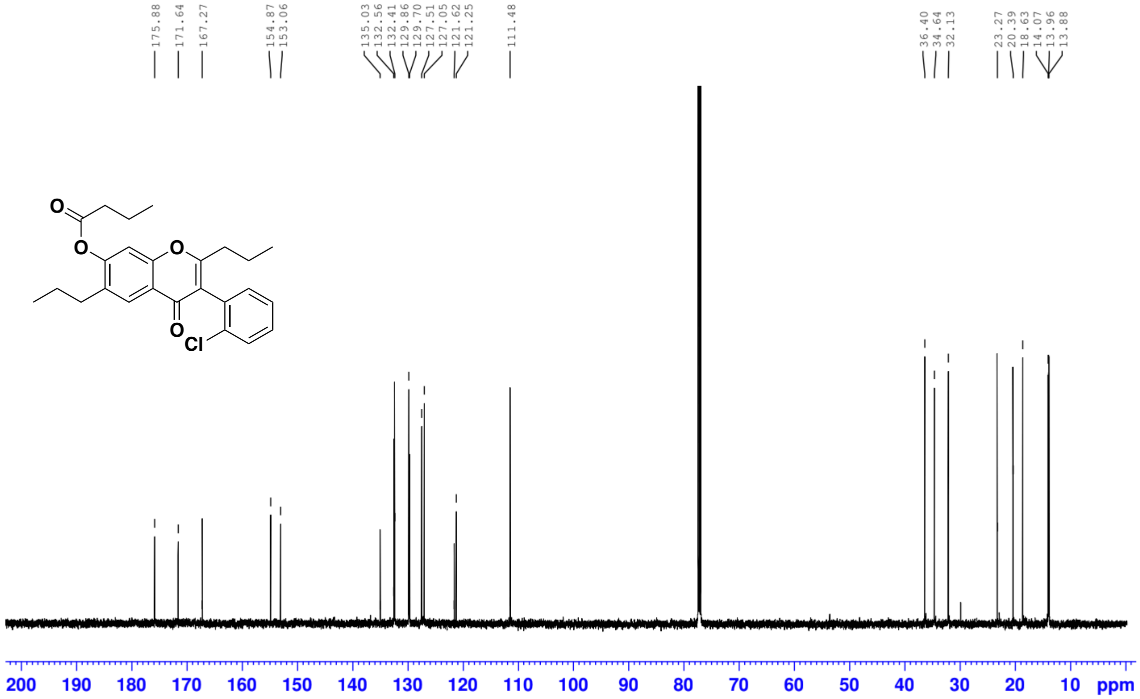
175.88
171.64
167.27
154.87
153.06
135.03
132.56
132.41
129.86
129.70
127.51
127.05
121.62
121.25
111.48
36.40
34.64
32.13
23.27
20.39
18.63
14.07
13.96
13.88
O
O
O
O
Cl
200 190 180 170 160 150 140 130 120 110 100 90 80 70 60 50 40 30 20 10 ppm

JG-30

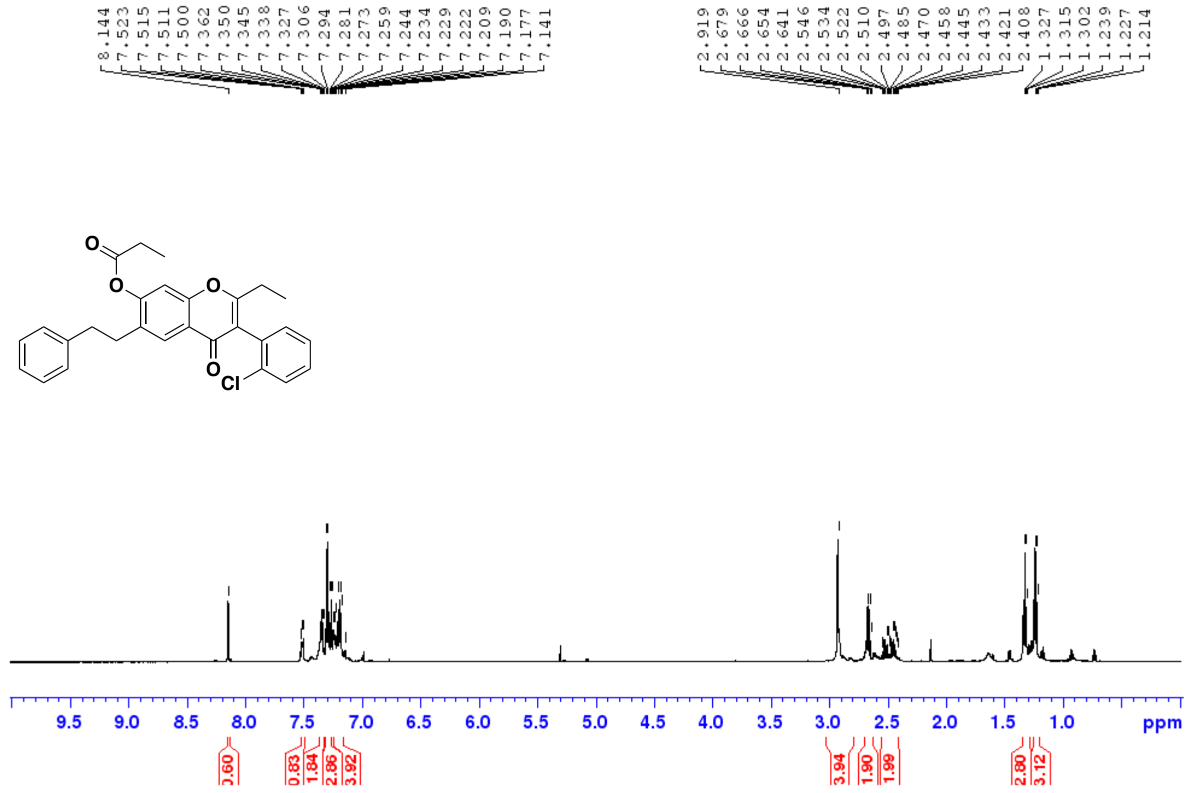

8.144
7.523
7.515
7.511
7.500
7.362
7.350
7.345
7.338
7.327
7.306
7.294
7.281
7.273
7.259
7.244
7.234
7.229
7.222
7.209
7.190
7.177
7.141
2.919
2.679
2.666
2.654
2.641
2.546
2.534
2.522
2.510
2.497
2.485
2.470
2.458
2.445
2.433
2.421
2.408
1.327
1.315
1.302
1.239
1.227
1.214
O
O
O
O
Cl
ppm


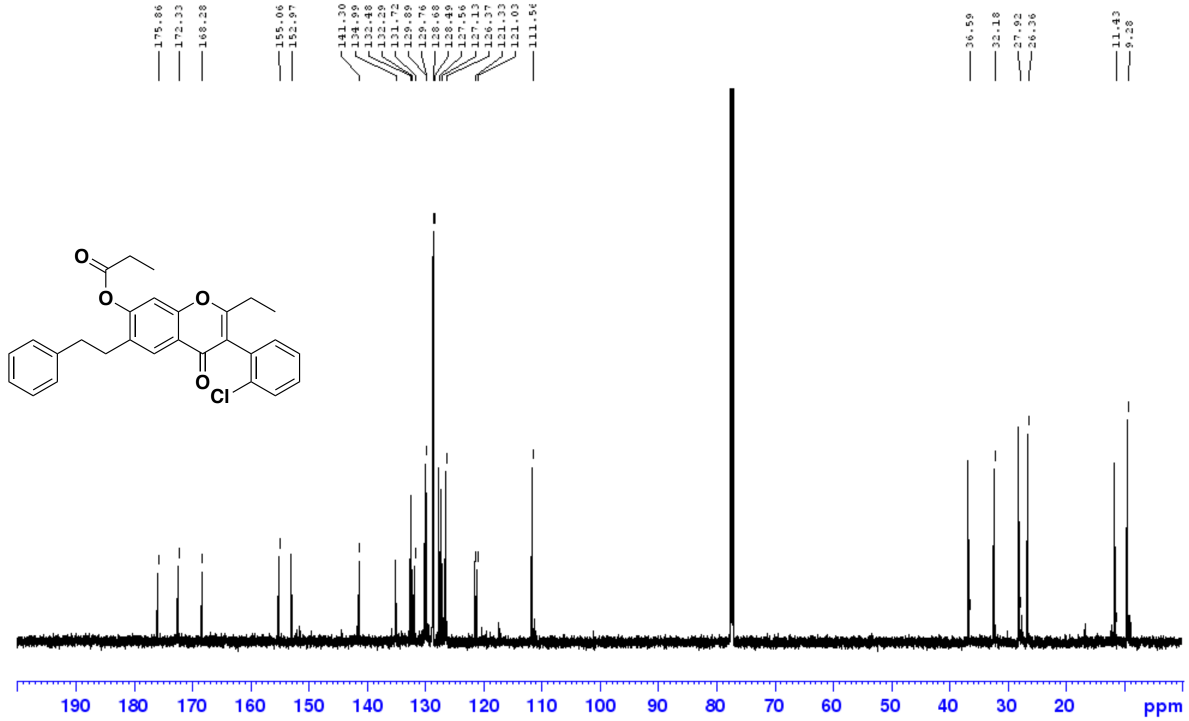

175.86
172.33
168.28
155.06
152.97
141.30
134.99
132.48
132.29
131.72
129.89
129.76
128.68
128.49
127.56
127.13
126.37
121.33
121.03
36.59
32.18
27.92
26.36
11.43
9.28
O
O
O
O
Cl
ppm

JG-31

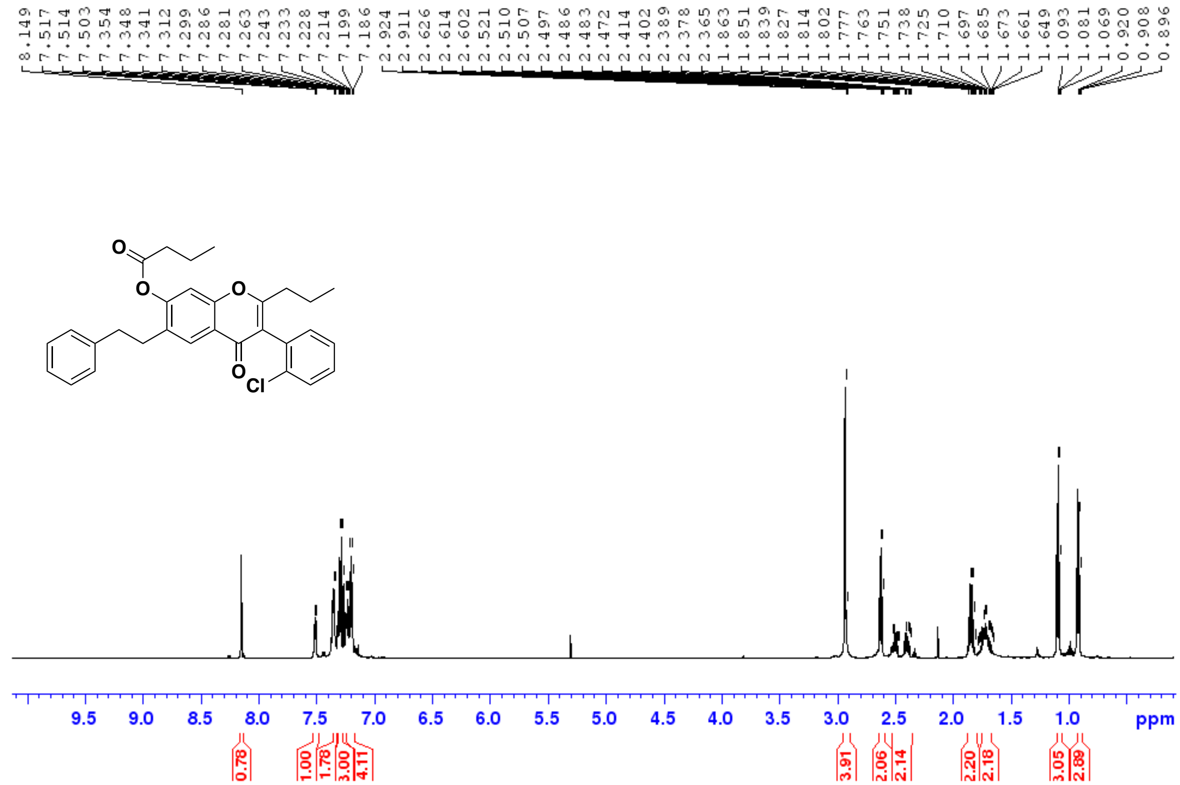

8.149 7.517 7.514 7.503 7.354 7.348 7.341 7.312 7.299 7.286 7.281 7.263 7.243 7.233 7.228 7.214 7.199 7.186 2.924 2.911 2.626 2.614 2.602 2.521 2.510 2.507 2.497 2.486 2.483 2.472 2.414 2.402 2.389 2.378 2.365 1.863 1.851 1.839 1.827 1.814 1.802 1.777 1.763 1.751 1.738 1.725 1.710 1.697 1.685 1.673 1.661 1.649 1.093 1.081 1.069 0.920 0.908 0.896
O
O
O
O
Cl
9.5 9.0 8.5 8.0 7.5 7.0 6.5 6.0 5.5 5.0 4.5 4.0 3.5 3.0 2.5 2.0 1.5 1.0 ppm
0.78 1.00 1.78 3.00 4.11 3.91 2.06 2.14 2.20 2.18 3.05 2.89


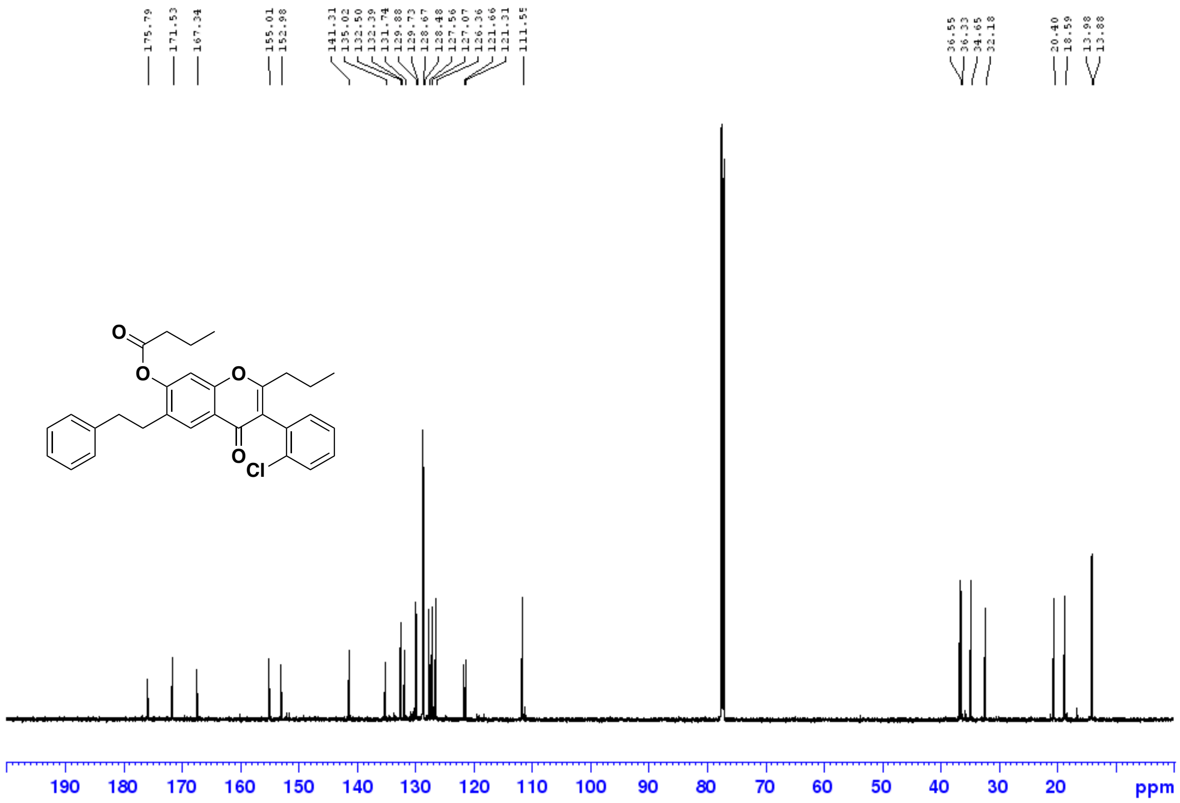

175.79 171.53 167.34 155.01 152.98 141.31 135.02 132.50 132.39 131.74 129.88 129.73 128.67 128.48 127.56 127.07 126.36 121.66 121.31 111.55 36.55 36.33 34.65 32.18 20.40 18.59 13.98 13.88
O
O
O
O
Cl
190 180 170 160 150 140 130 120 110 100 90 80 70 60 50 40 30 20 ppm

JG-34

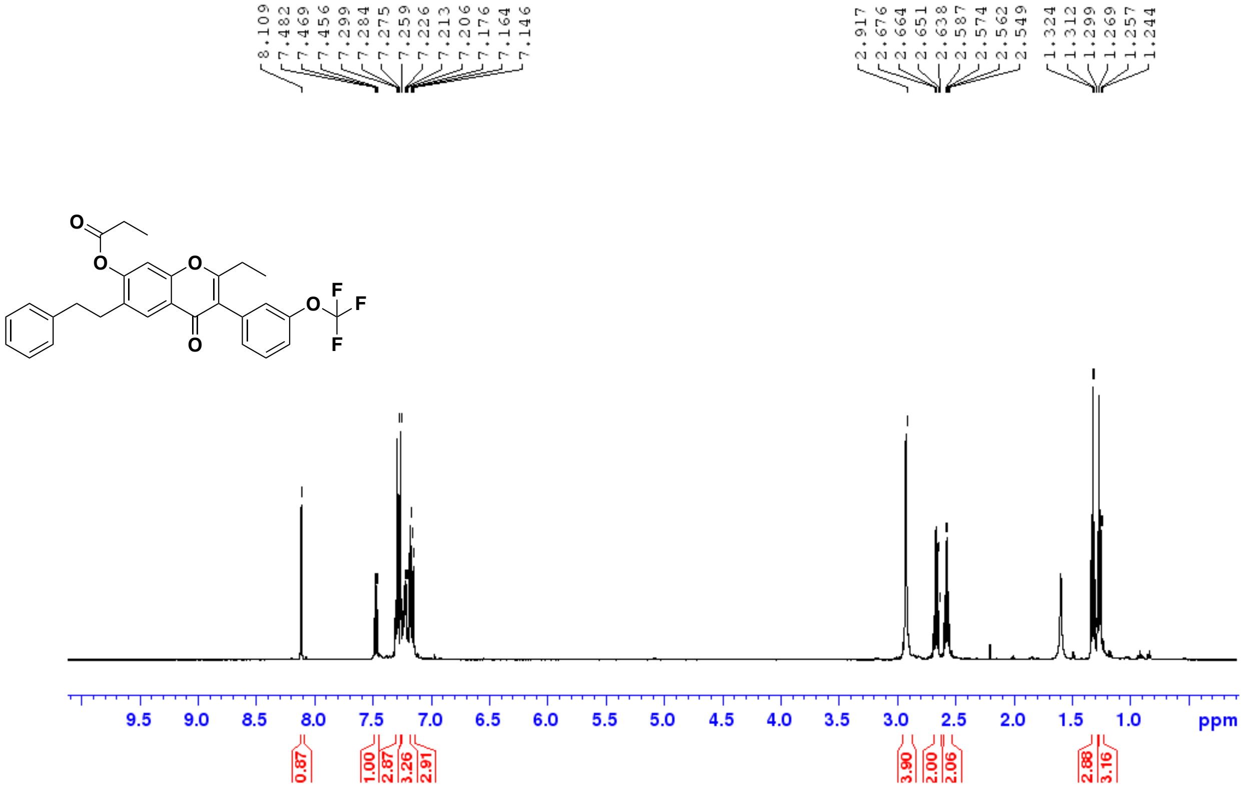

8.109
7.482
7.469
7.456
7.299
7.284
7.275
7.259
7.226
7.213
7.206
7.176
7.164
7.146
2.917
2.676
2.664
2.651
2.638
2.587
2.574
2.562
2.549
1.324
1.312
1.299
1.269
1.257
1.244
O
O
O
O
O
F
F
F
9.5 9.0 8.5 8.0 7.5 7.0 6.5 6.0 5.5 5.0 4.5 4.0 3.5 3.0 2.5 2.0 1.5 1.0 ppm
0.87
1.00
2.87
3.26
2.91
3.90
2.00
2.06
2.88
3.16


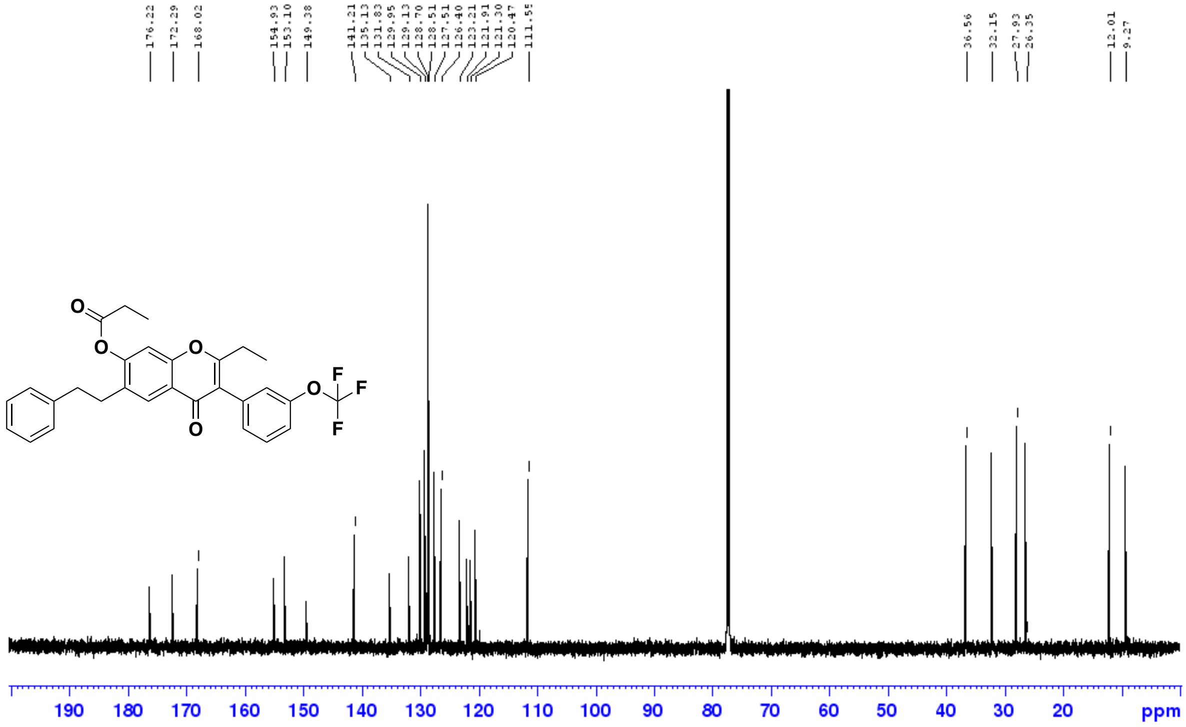

176.22
172.29
168.02
154.93
153.10
149.38
141.21
135.13
131.83
129.95
129.13
128.70
128.51
127.51
126.40
123.21
121.91
121.30
120.47
111.55
36.56
32.15
27.93
26.35
12.01
9.27
O
O
O
O
O
F
F
F
190 180 170 160 150 140 130 120 110 100 90 80 70 60 50 40 30 20 ppm

JG-35

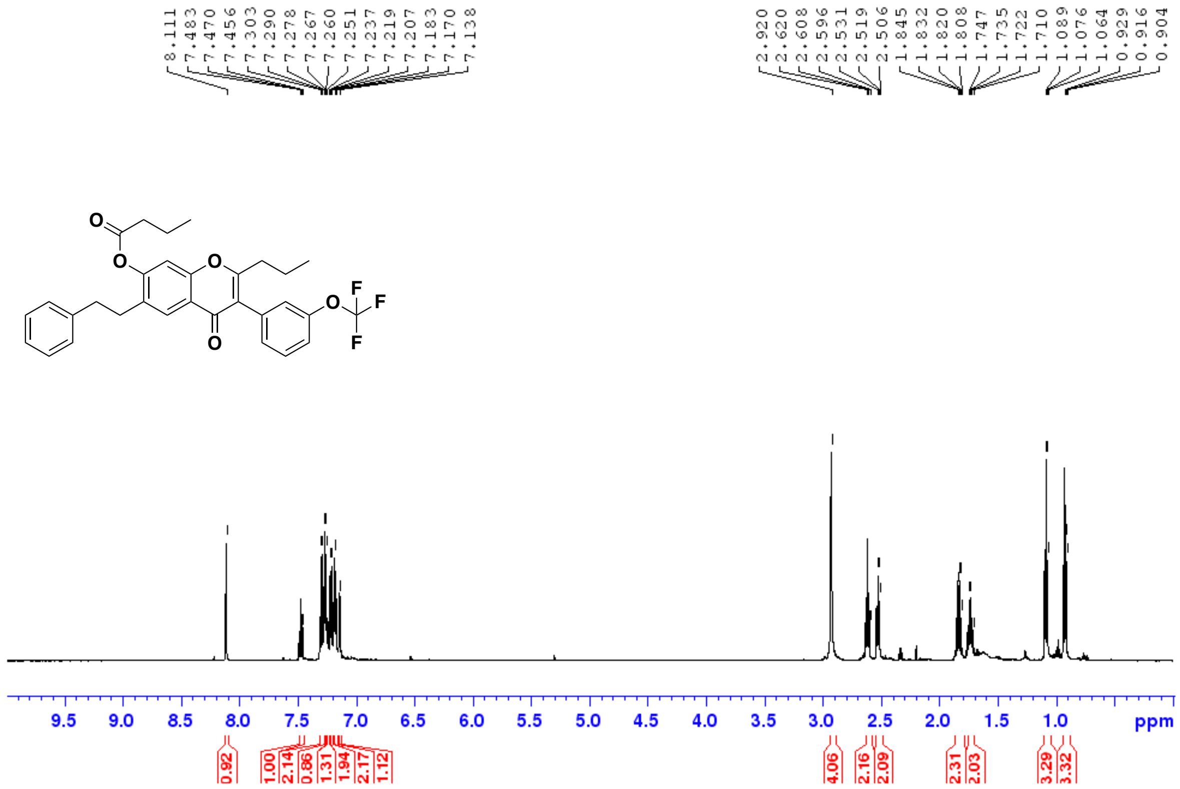

8.111
7.483
7.470
7.456
7.303
7.290
7.278
7.267
7.260
7.251
7.237
7.219
7.207
7.183
7.170
7.138
2.920
2.620
2.608
2.596
2.531
2.519
2.506
1.845
1.832
1.820
1.808
1.747
1.735
1.722
1.710
1.089
1.076
1.064
0.929
0.916
0.904
O
O
O
O
O
F
F
F
9.5 9.0 8.5 8.0 7.5 7.0 6.5 6.0 5.5 5.0 4.5 4.0 3.5 3.0 2.5 2.0 1.5 1.0 ppm
0.92
1.00
2.14
0.86
1.31
1.94
2.17
1.12
4.06
2.16
2.09
2.31
2.03
3.29
3.32


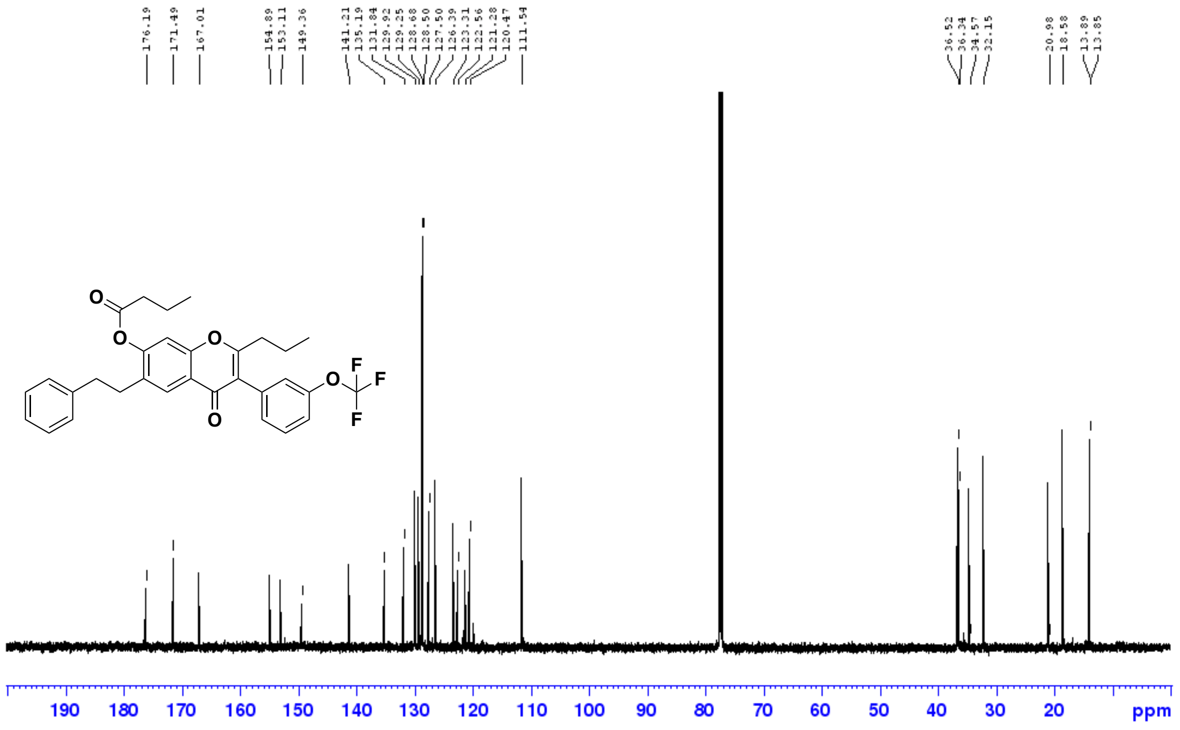

176.19
171.49
167.01
154.89
153.11
149.36
141.21
135.19
131.84
129.92
129.25
128.68
128.50
127.50
126.39
123.31
122.56
121.28
120.47
111.54
36.52
36.34
34.57
32.15
20.98
18.58
13.89
13.85
O
O
O
O
O
F
F
F
190 180 170 160 150 140 130 120 110 100 90 80 70 60 50 40 30 20 ppm

JG-37

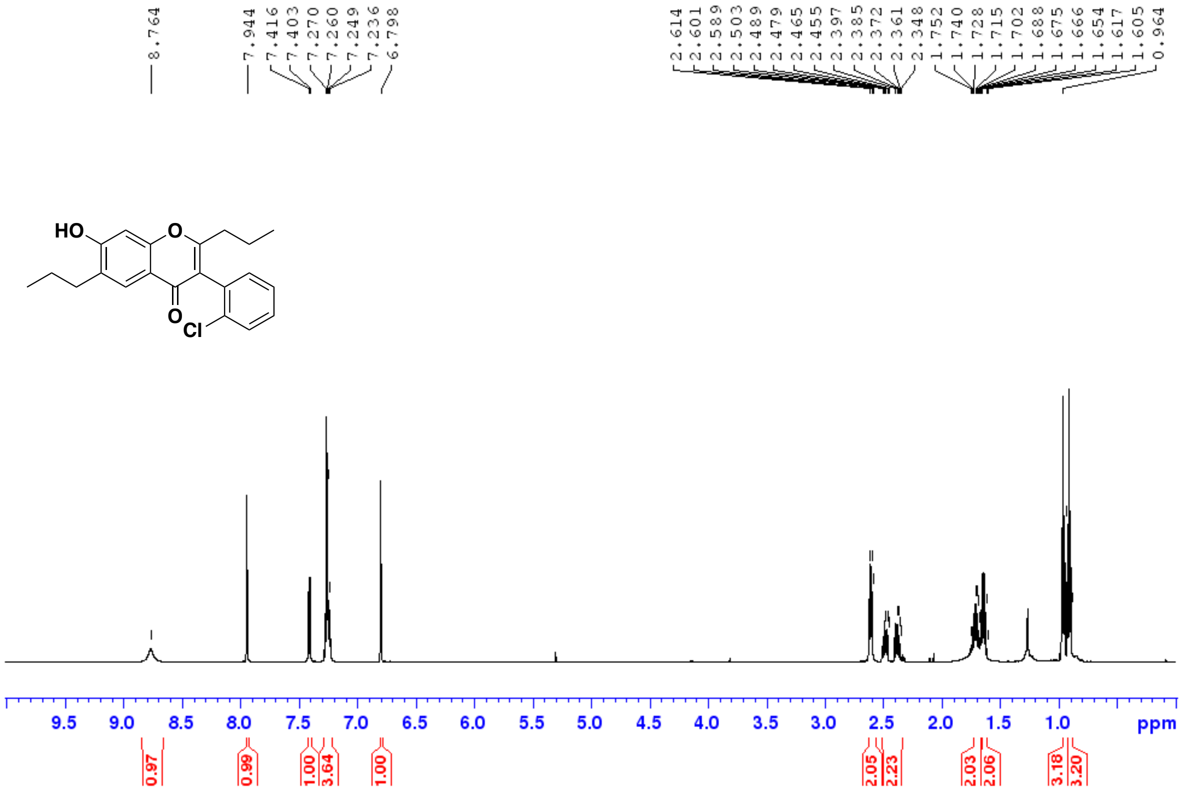
8.764
7.944
7.416
7.403
7.270
7.260
7.249
7.236
6.798
2.614
2.601
2.589
2.503
2.489
2.479
2.465
2.455
2.397
2.385
2.372
2.361
2.348
1.752
1.740
1.728
1.715
1.702
1.688
1.675
1.666
1.654
1.617
1.605
0.964
HO
O
O
Cl
9.5 9.0 8.5 8.0 7.5 7.0 6.5 6.0 5.5 5.0 4.5 4.0 3.5 3.0 2.5 2.0 1.5 1.0 ppm
0.97
0.99
1.00
3.64
1.00
2.05
2.23
2.03
2.06
3.18
3.20

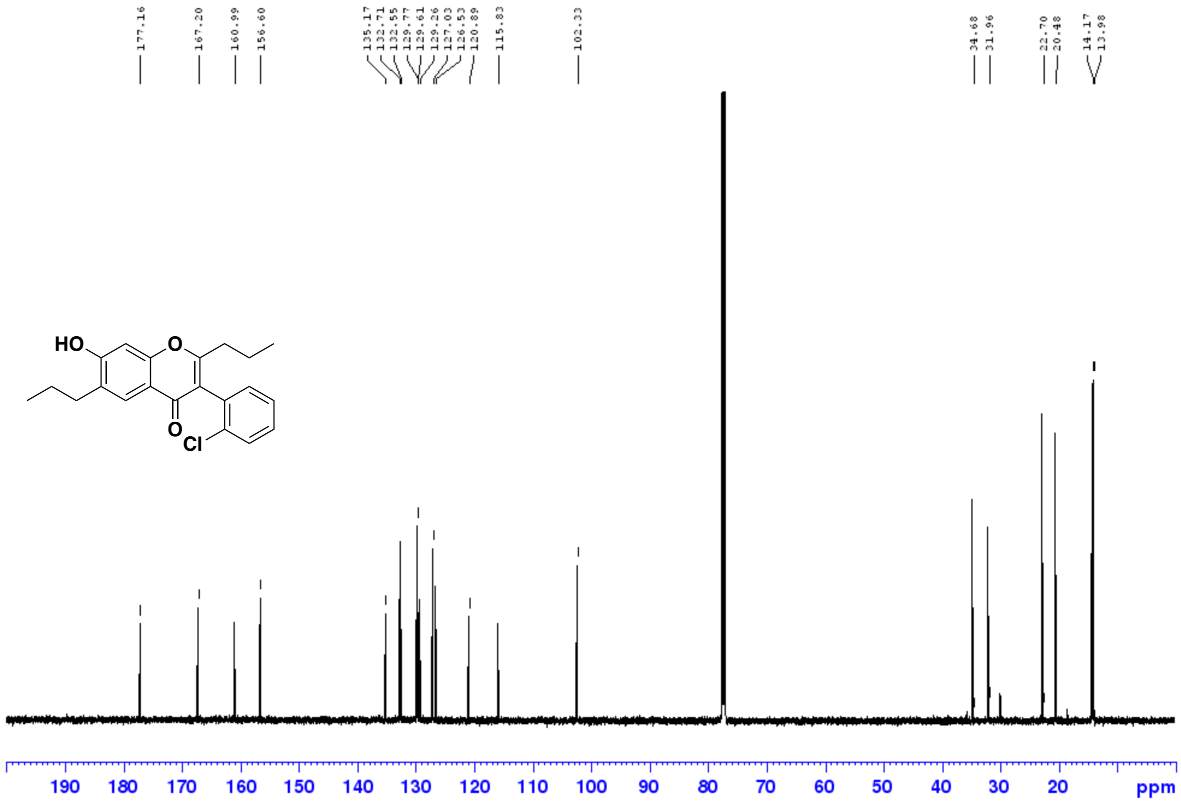
177.16
167.20
160.99
156.60
135.17
132.71
132.55
129.77
129.61
129.26
127.03
126.53
120.89
115.83
102.33
34.68
31.96
22.70
20.48
14.17
13.98
HO
O
O
Cl
190 180 170 160 150 140 130 120 110 100 90 80 70 60 50 40 30 20 ppm

JG-38

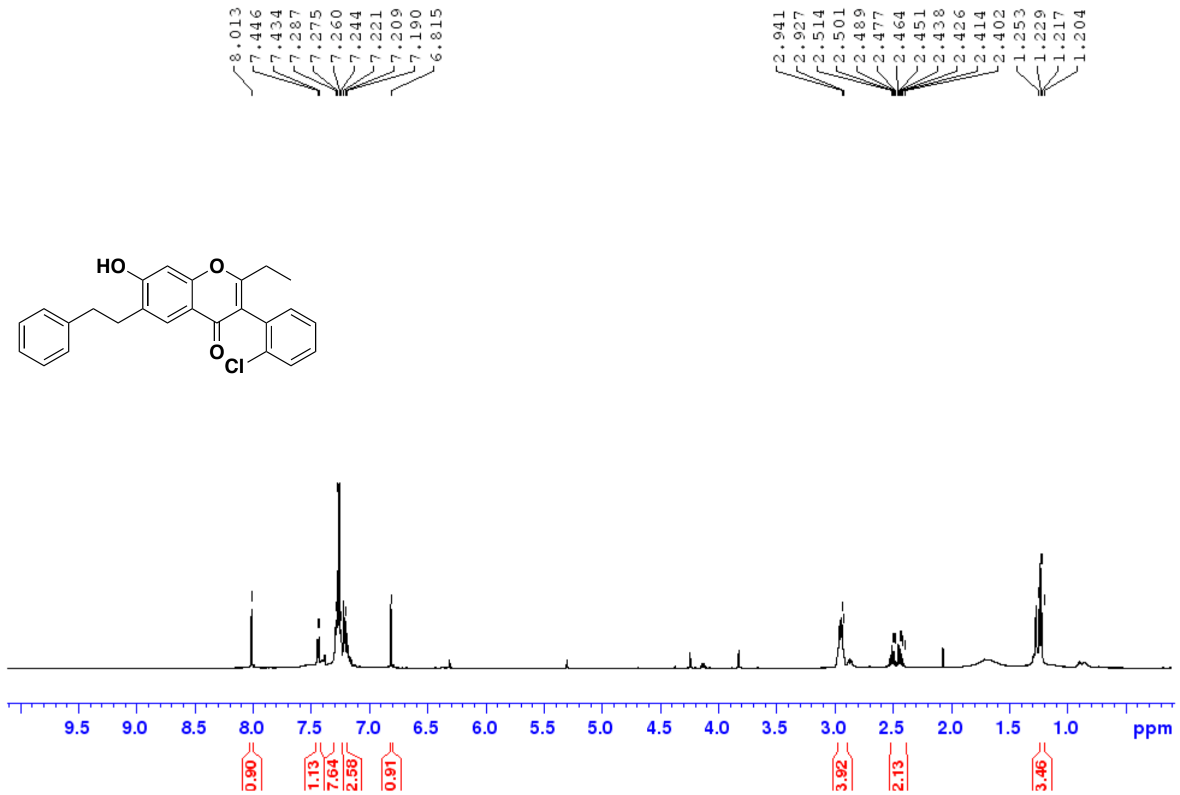
8.013
7.446
7.434
7.287
7.275
7.260
7.244
7.221
7.209
7.190
6.815
2.941
2.927
2.514
2.501
2.489
2.477
2.464
2.451
2.438
2.426
2.414
2.402
1.253
1.229
1.217
1.204
HO
O
O
Cl
9.5 9.0 8.5 8.0 7.5 7.0 6.5 6.0 5.5 5.0 4.5 4.0 3.5 3.0 2.5 2.0 1.5 1.0 ppm
0.90
1.13
7.64
2.58
0.91
3.92
2.13
3.46

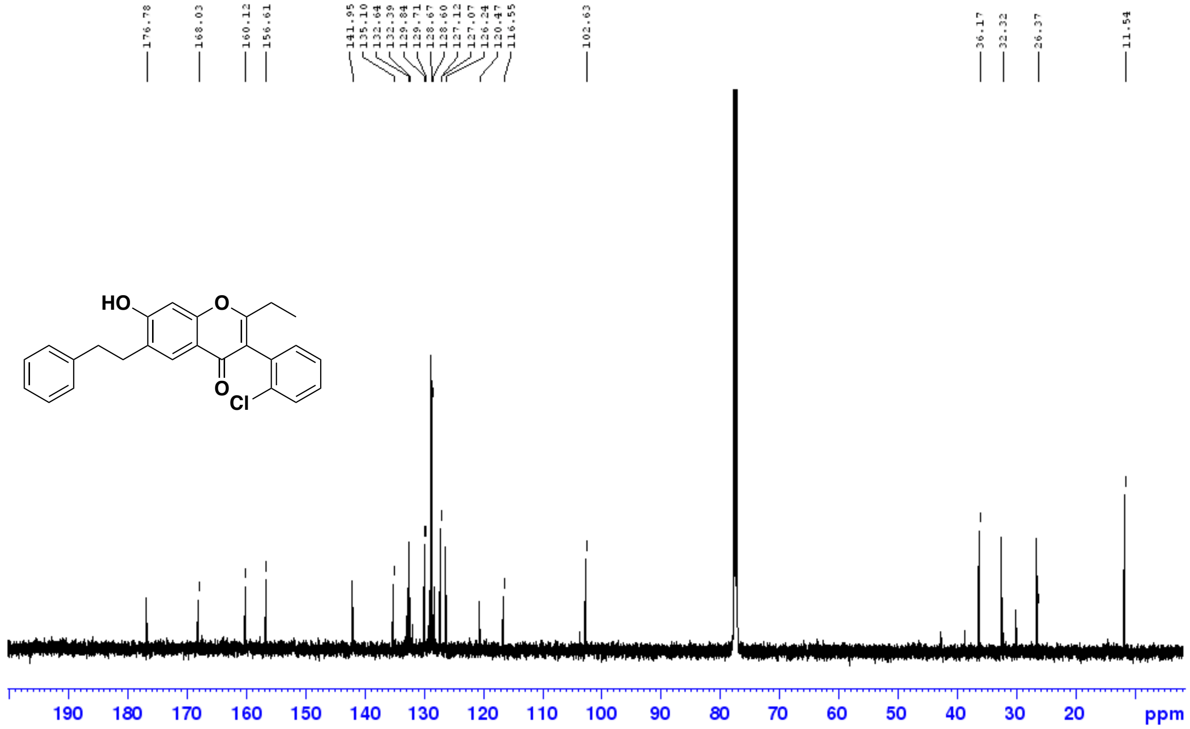
176.78
168.03
160.12
156.61
141.95
135.10
132.64
132.39
129.84
129.71
128.67
128.60
127.12
127.07
126.24
120.47
116.55
102.63
36.17
32.32
26.37
11.54
HO
O
O
Cl
190 180 170 160 150 140 130 120 110 100 90 80 70 60 50 40 30 20 ppm

JG-39

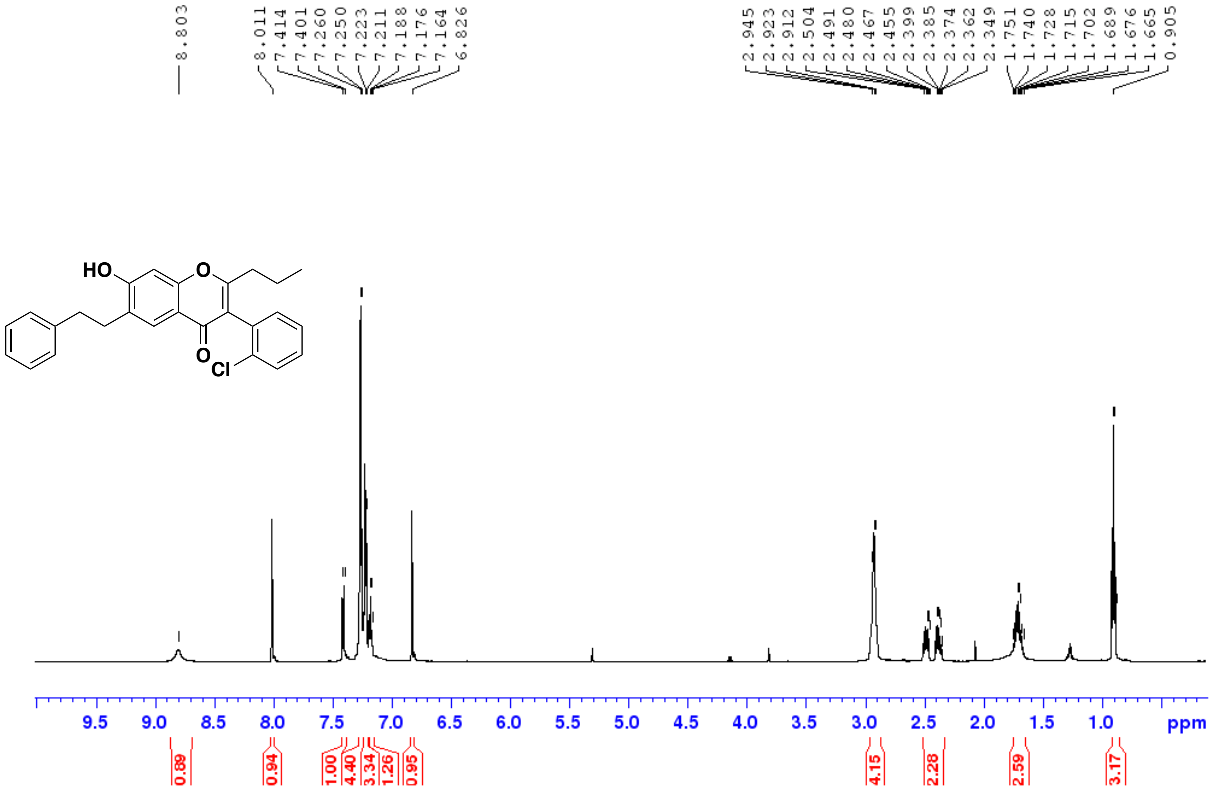
8.803
8.011
7.414
7.401
7.260
7.250
7.223
7.211
7.188
7.176
7.164
6.826
2.945
2.923
2.912
2.504
2.491
2.480
2.467
2.455
2.399
2.385
2.374
2.362
2.349
1.751
1.740
1.728
1.715
1.702
1.689
1.676
1.665
0.905
HO
O
O
Cl
9.5 9.0 8.5 8.0 7.5 7.0 6.5 6.0 5.5 5.0 4.5 4.0 3.5 3.0 2.5 2.0 1.5 1.0 ppm
0.89
0.94
1.00
4.40
3.34
1.26
0.95
4.15
2.28
2.59
3.17

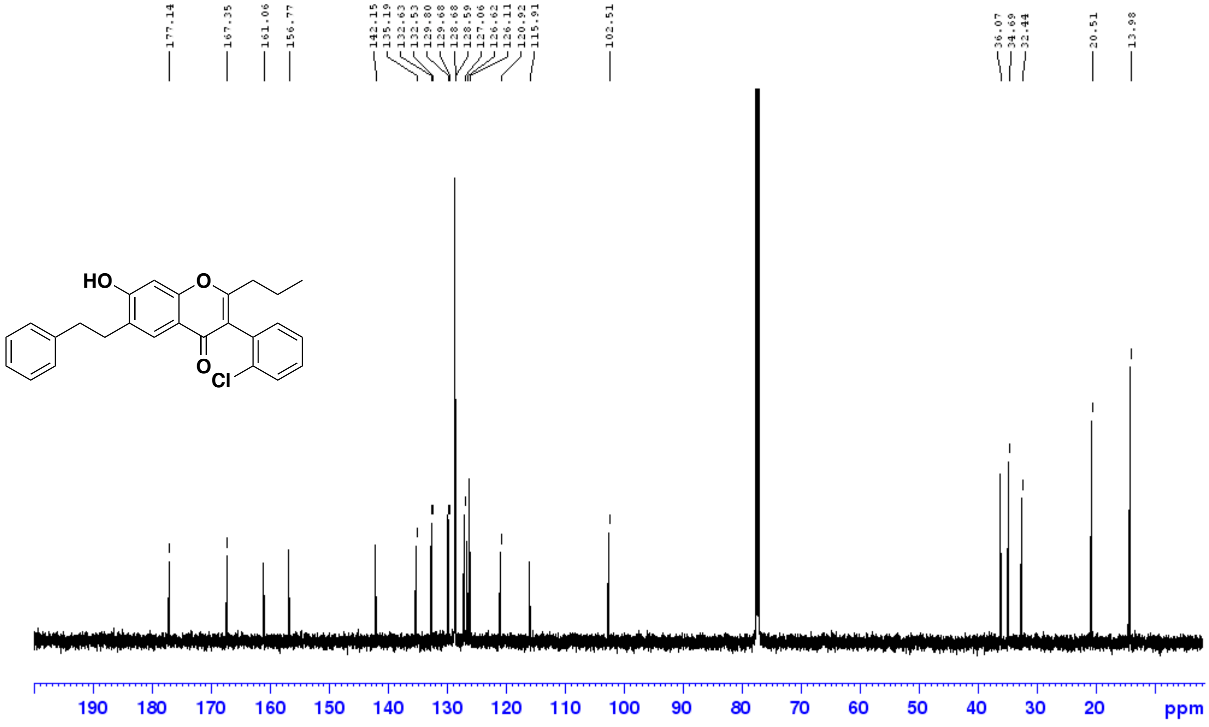
177.14
167.35
161.06
156.77
142.15
135.19
132.63
132.53
129.80
129.68
128.68
128.59
127.06
126.62
126.11
120.92
115.91
102.51
36.07
34.69
32.44
20.51
13.98
HO
O
O
Cl
190 180 170 160 150 140 130 120 110 100 90 80 70 60 50 40 30 20 ppm

JG-41

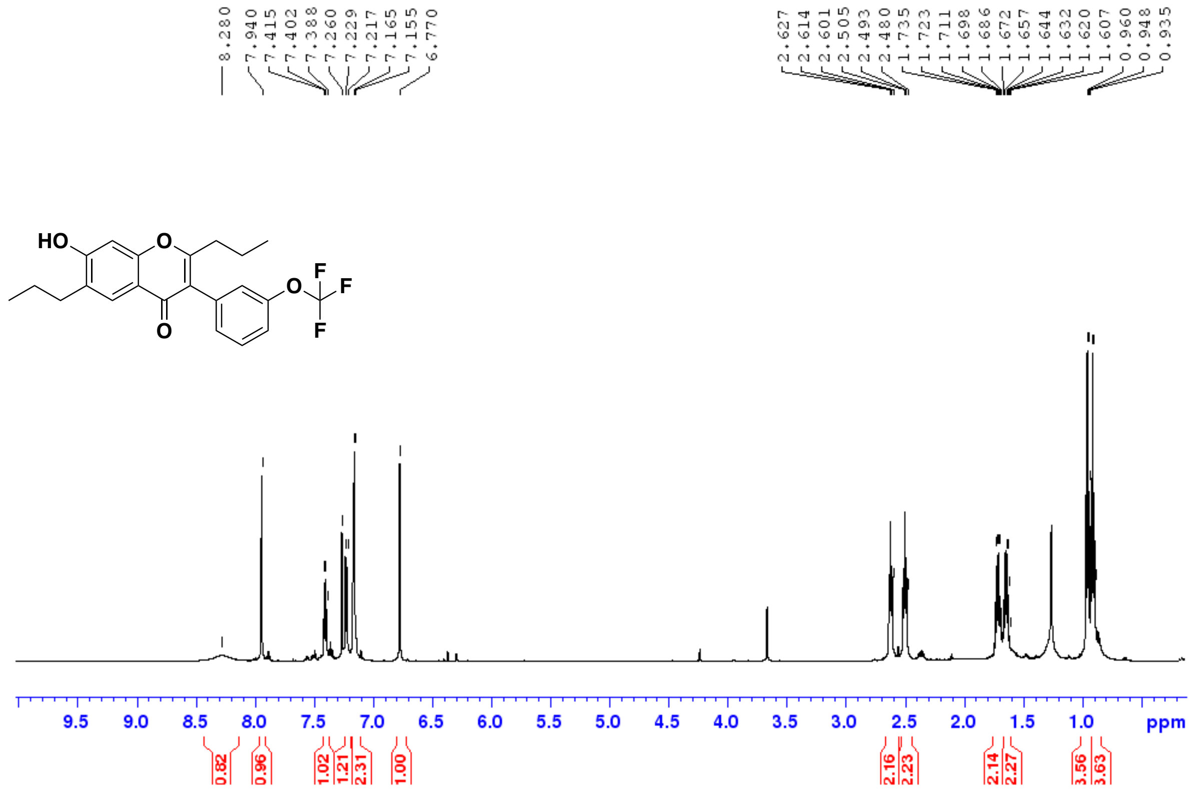

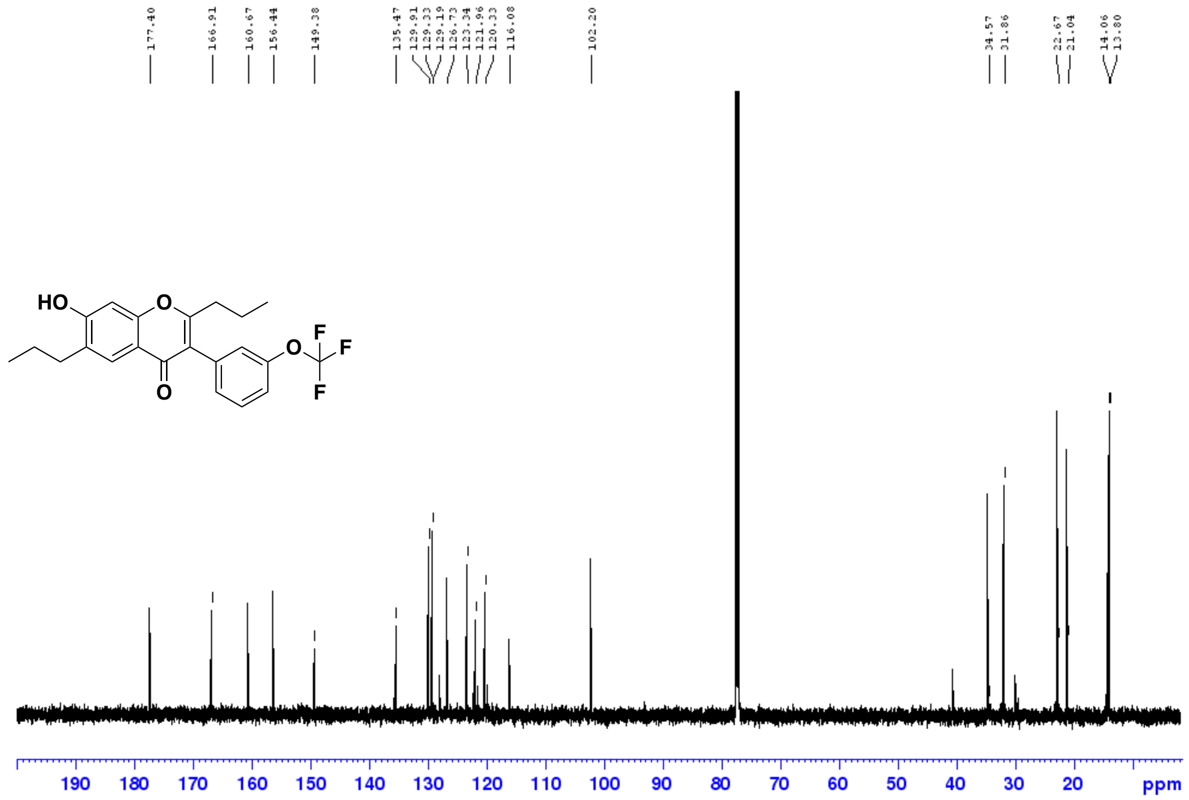

JG-42

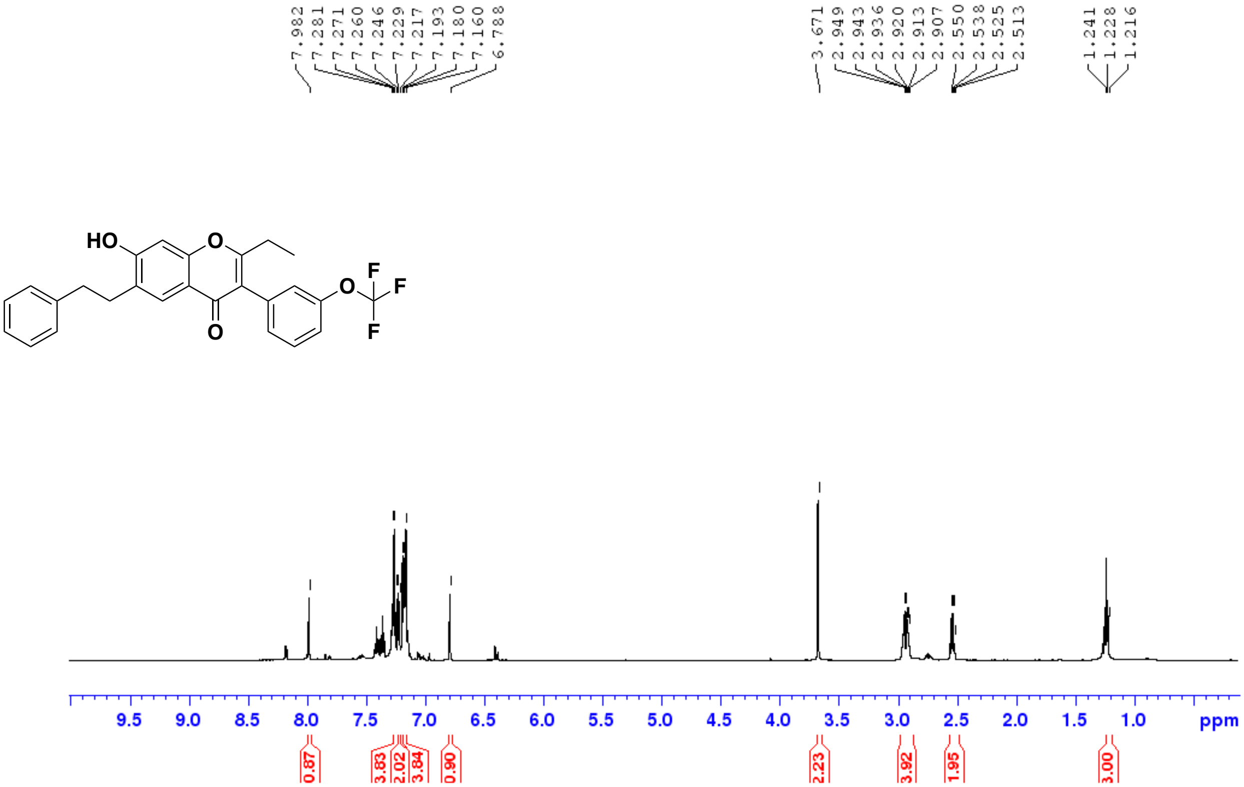
7.982
7.281
7.271
7.260
7.246
7.229
7.217
7.193
7.180
7.160
6.788
3.671
2.949
2.943
2.936
2.920
2.913
2.907
2.550
2.538
2.525
2.513
1.241
1.228
1.216
HO
O
O
O
F
F
F
9.5 9.0 8.5 8.0 7.5 7.0 6.5 6.0 5.5 5.0 4.5 4.0 3.5 3.0 2.5 2.0 1.5 1.0 ppm
0.87
3.83
2.02
3.84
0.90
2.23
3.92
1.95
3.00

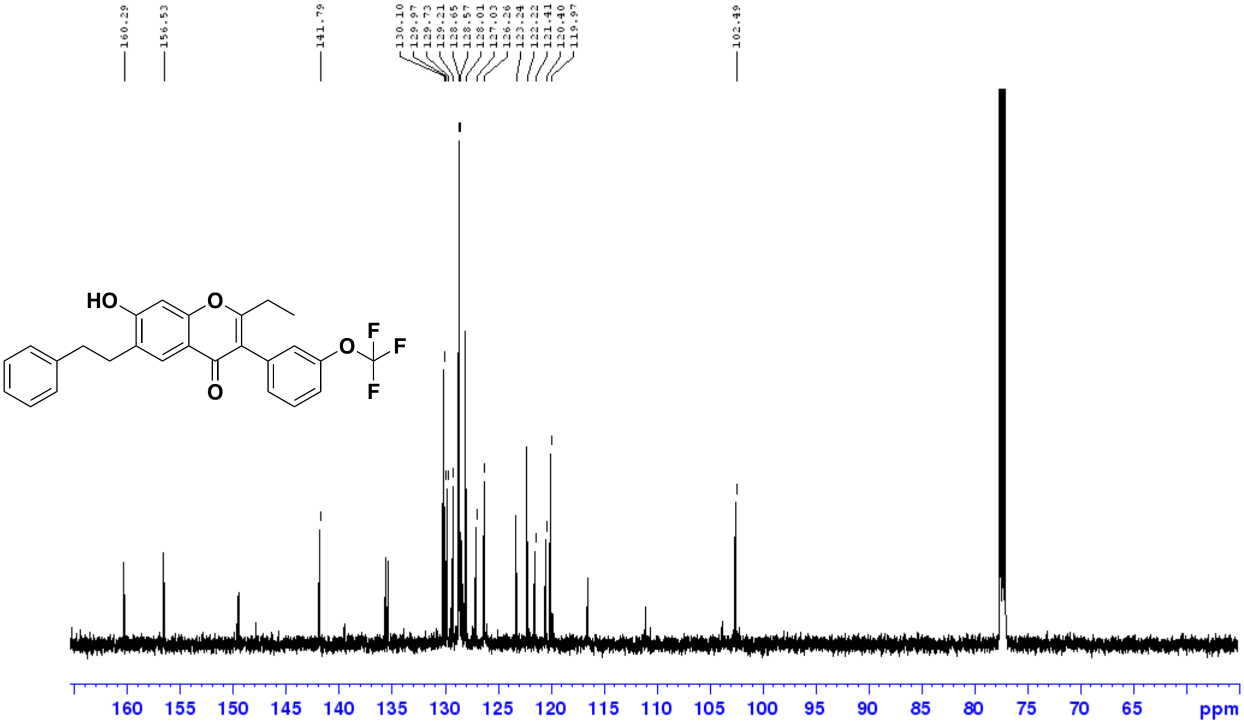
160.29
156.53
141.79
130.10
129.97
129.73
129.21
128.65
128.57
128.01
127.03
126.26
123.24
122.22
121.41
120.40
119.97
102.49
HO
O
O
O
F
F
F
160 155 150 145 140 135 130 125 120 115 110 105 100 95 90 85 80 75 70 65 ppm

JG-43

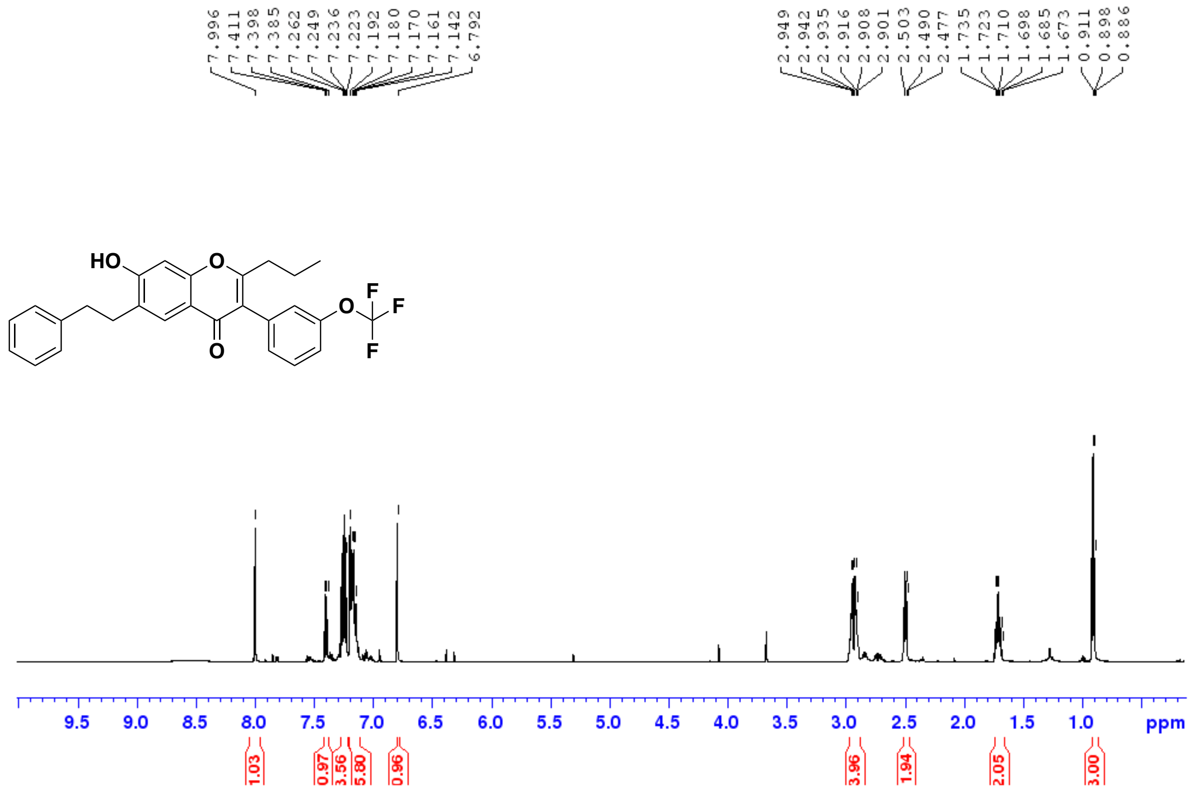
7.996
7.411
7.398
7.385
7.262
7.249
7.236
7.223
7.192
7.180
7.170
7.161
7.142
6.792
2.949
2.942
2.935
2.916
2.908
2.901
2.503
2.490
2.477
1.735
1.723
1.710
1.698
1.685
1.673
0.911
0.898
0.886
HO
O
O
F
F
F
O
9.5
9.0
8.5
8.0
7.5
7.0
6.5
6.0
5.5
5.0
4.5
4.0
3.5
3.0
2.5
2.0
1.5
1.0
ppm
1.03
0.97
3.56
5.80
0.96
3.96
1.94
2.05
3.00

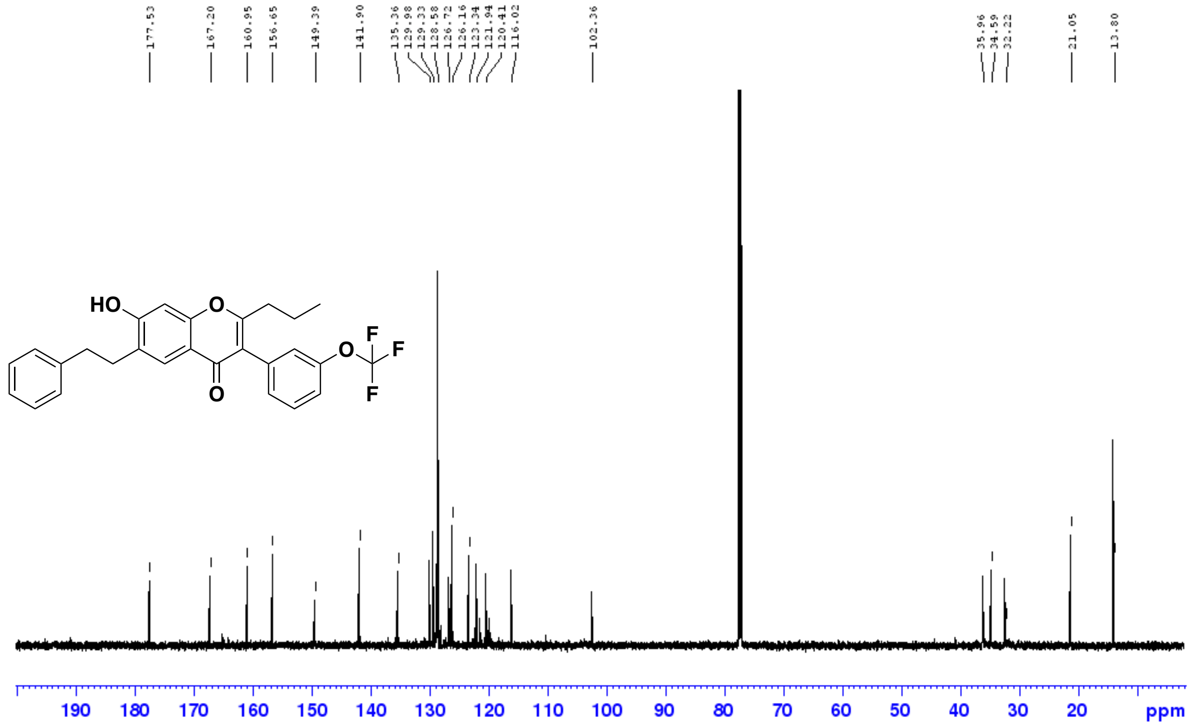
177.53
167.20
160.95
156.65
149.39
141.90
135.36
129.98
129.33
128.58
126.72
126.16
123.34
121.94
120.41
116.02
102.36
35.96
34.59
32.22
21.05
13.80
HO
O
O
F
F
F
O
190
180
170
160
150
140
130
120
110
100
90
80
70
60
50
40
30
20
ppm

JG-44

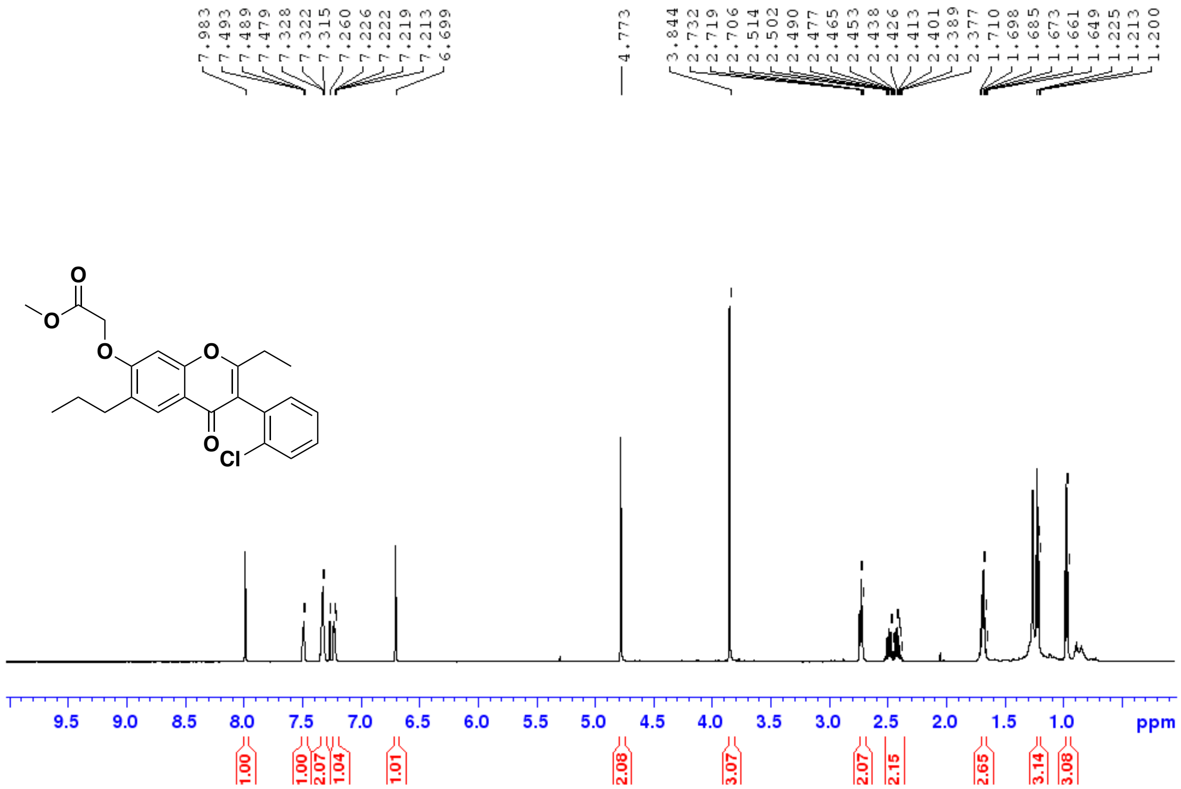

7.983
7.493
7.489
7.479
7.328
7.322
7.315
7.260
7.226
7.222
7.219
7.213
6.699
4.773
3.844
2.732
2.719
2.706
2.514
2.502
2.490
2.477
2.465
2.453
2.438
2.426
2.413
2.401
2.389
2.377
1.710
1.698
1.685
1.673
1.661
1.649
1.225
1.213
1.200
O
O
O
O
Cl
9.5 9.0 8.5 8.0 7.5 7.0 6.5 6.0 5.5 5.0 4.5 4.0 3.5 3.0 2.5 2.0 1.5 1.0 ppm
1.00
1.00
2.07
1.04
1.01
2.08
3.07
2.07
2.15
2.65
3.14
3.08


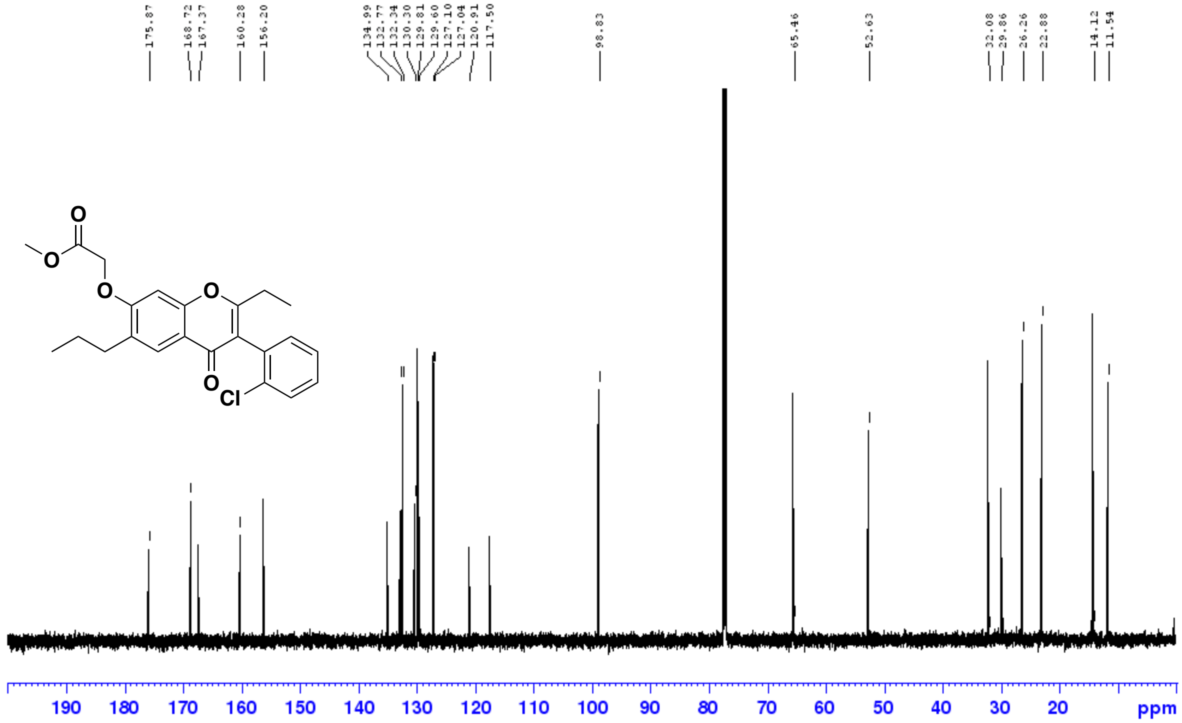

175.87
168.72
167.37
160.28
156.20
134.99
132.77
132.34
130.30
129.81
129.60
127.10
127.04
120.91
117.50
98.83
65.46
52.63
32.08
29.86
26.26
22.88
14.12
11.54
O
O
O
O
Cl
190 180 170 160 150 140 130 120 110 100 90 80 70 60 50 40 30 20 ppm

JG-45

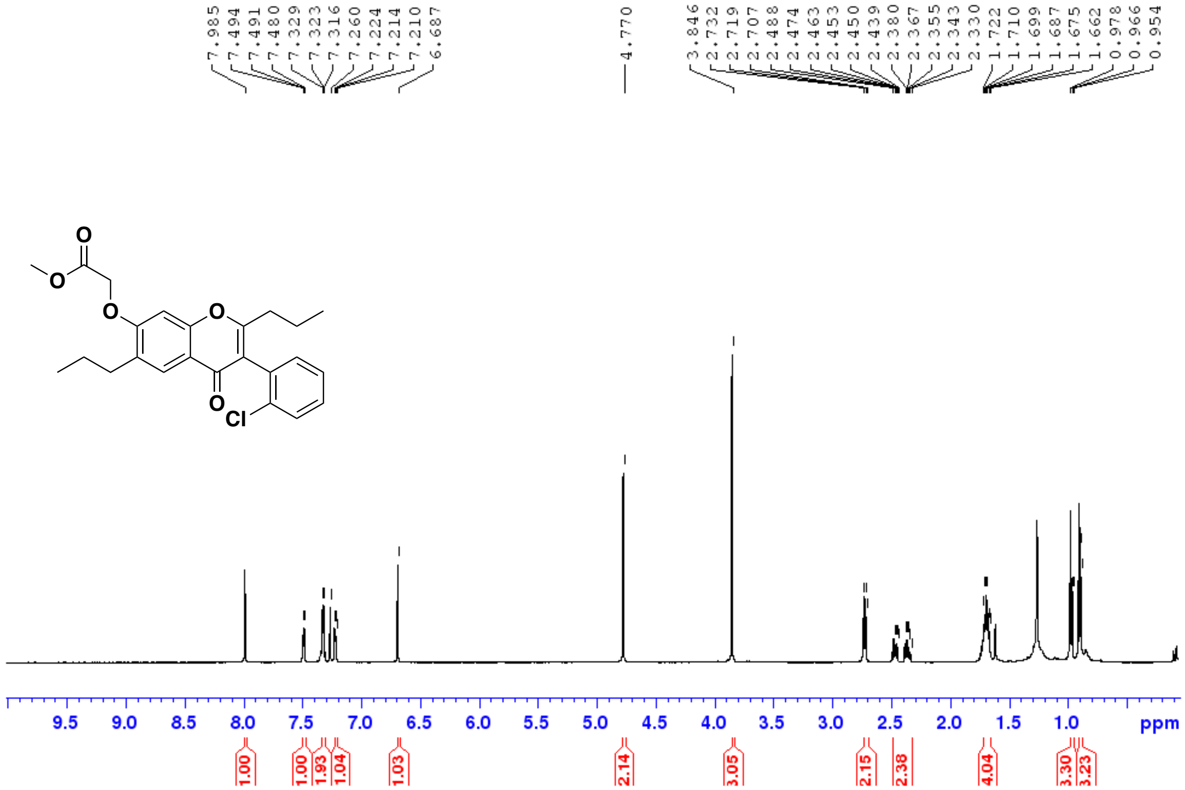
7.985
7.494
7.491
7.480
7.329
7.323
7.316
7.260
7.224
7.214
7.210
6.687
4.770
3.846
2.732
2.719
2.707
2.488
2.474
2.463
2.453
2.450
2.439
2.380
2.367
2.355
2.343
2.330
1.722
1.710
1.699
1.687
1.675
1.662
0.978
0.966
0.954
O
O
O
O
O
Cl
9.5 9.0 8.5 8.0 7.5 7.0 6.5 6.0 5.5 5.0 4.5 4.0 3.5 3.0 2.5 2.0 1.5 1.0 ppm
1.00
1.00
1.93
1.04
1.03
2.14
3.05
2.15
2.38
4.04
3.30
3.23

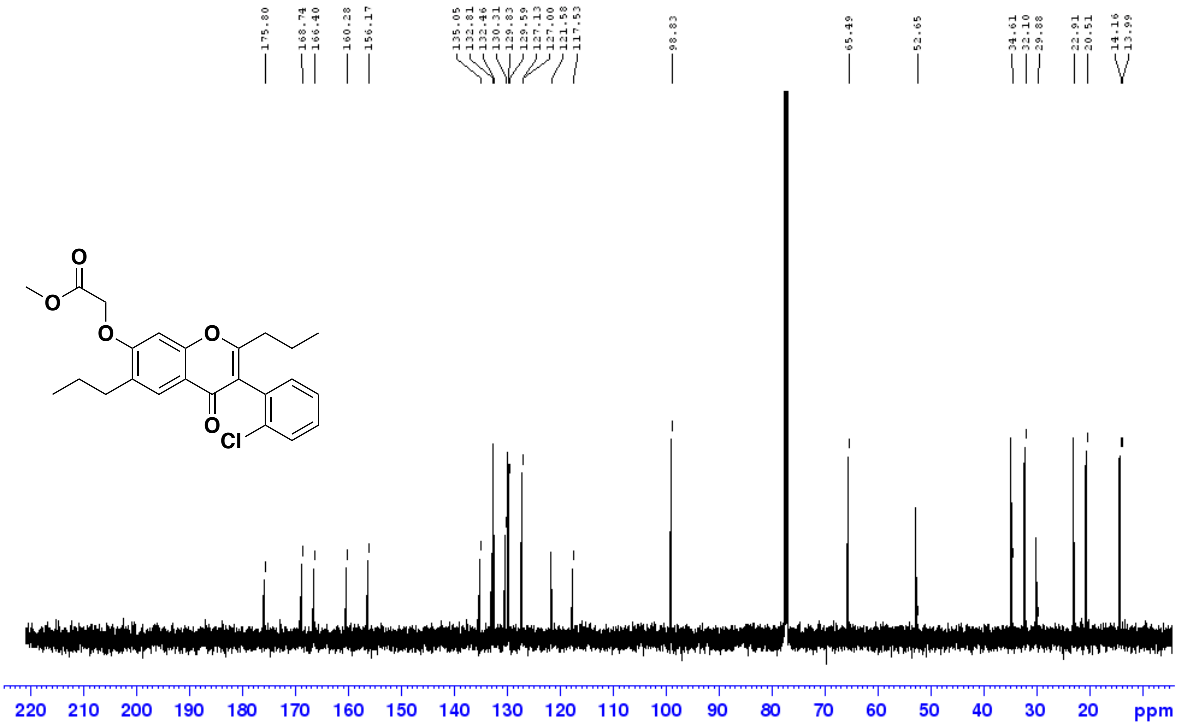
175.80
168.74
166.40
160.28
156.17
135.05
132.81
132.46
130.31
129.83
129.59
127.13
127.00
121.58
117.53
98.83
65.49
52.65
34.61
32.10
29.88
22.91
20.51
14.16
13.99
O
O
O
O
O
Cl
220 210 200 190 180 170 160 150 140 130 120 110 100 90 80 70 60 50 40 30 20 ppm

JG-46

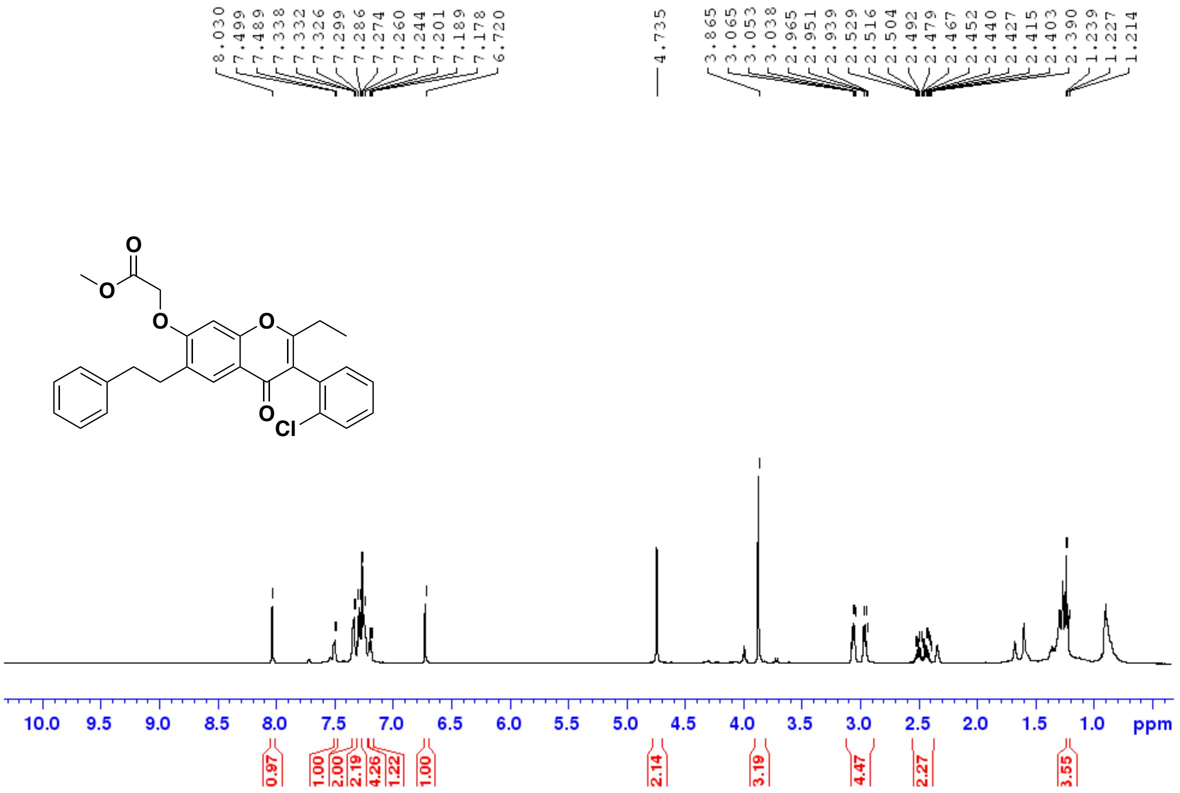

8.030
7.499
7.489
7.338
7.332
7.326
7.299
7.286
7.274
7.260
7.244
7.201
7.189
7.178
6.720
4.735
3.865
3.065
3.053
3.038
2.965
2.951
2.939
2.529
2.516
2.504
2.492
2.479
2.467
2.452
2.440
2.427
2.415
2.403
2.390
1.239
1.227
1.214
O
O
O
O
O
Cl
10.0
9.5
9.0
8.5
8.0
7.5
7.0
6.5
6.0
5.5
5.0
4.5
4.0
3.5
3.0
2.5
2.0
1.5
1.0
ppm
0.97
1.00
2.00
2.19
4.26
1.22
1.00
2.14
3.19
4.47
2.27
3.55


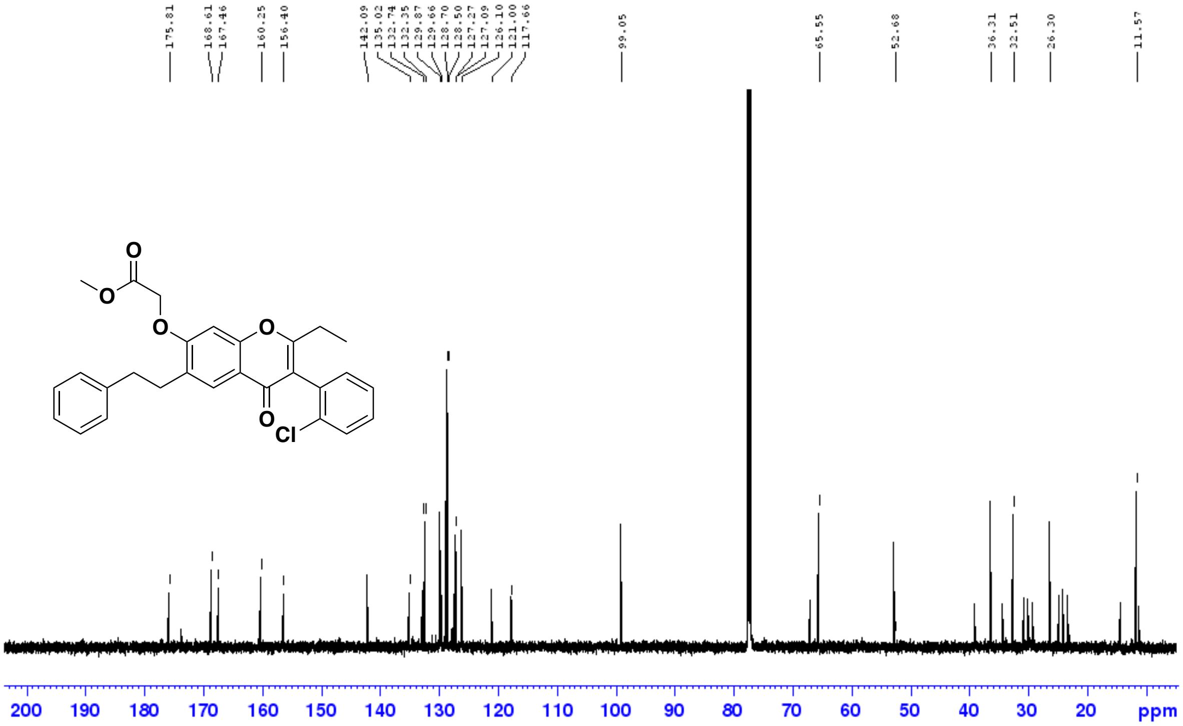

175.81
168.61
167.46
160.25
156.40
142.09
135.02
132.74
132.35
129.87
129.66
128.70
128.50
127.27
127.09
126.10
121.00
117.66
99.05
65.55
52.68
36.31
32.51
26.30
11.57
O
O
O
O
O
Cl
200
190
180
170
160
150
140
130
120
110
100
90
80
70
60
50
40
30
20
ppm

JG-47

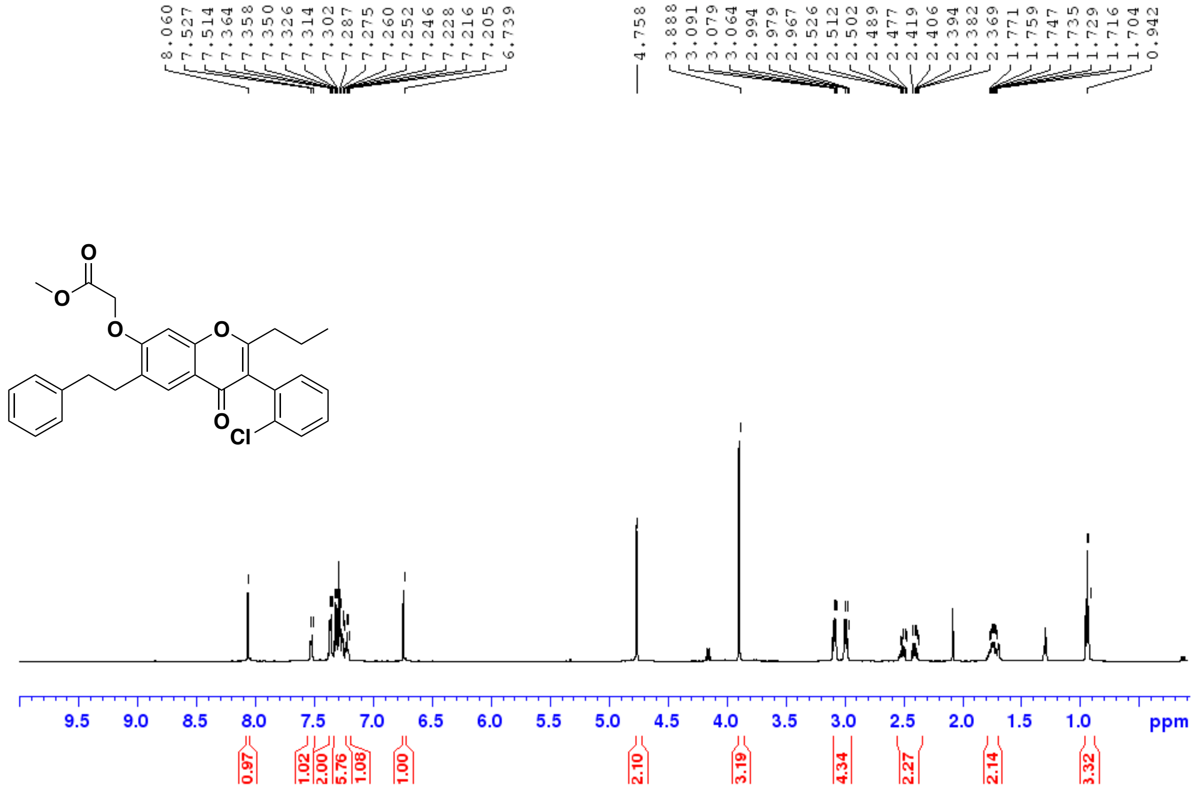
8.060 7.527 7.514 7.364 7.358 7.350 7.326 7.314 7.302 7.287 7.275 7.260 7.252 7.246 7.228 7.216 7.205 6.739
4.758 3.888 3.091 3.079 3.064 2.994 2.979 2.967 2.526 2.512 2.502 2.489 2.477 2.419 2.406 2.394 2.382 2.369 1.771 1.759 1.747 1.735 1.729 1.716 1.704 0.942
O
O
O
O
Cl
9.5 9.0 8.5 8.0 7.5 7.0 6.5 6.0 5.5 5.0 4.5 4.0 3.5 3.0 2.5 2.0 1.5 1.0 ppm
0.97 1.02 2.00 5.76 1.08 1.00 2.10 3.19 4.34 2.27 2.14 3.32

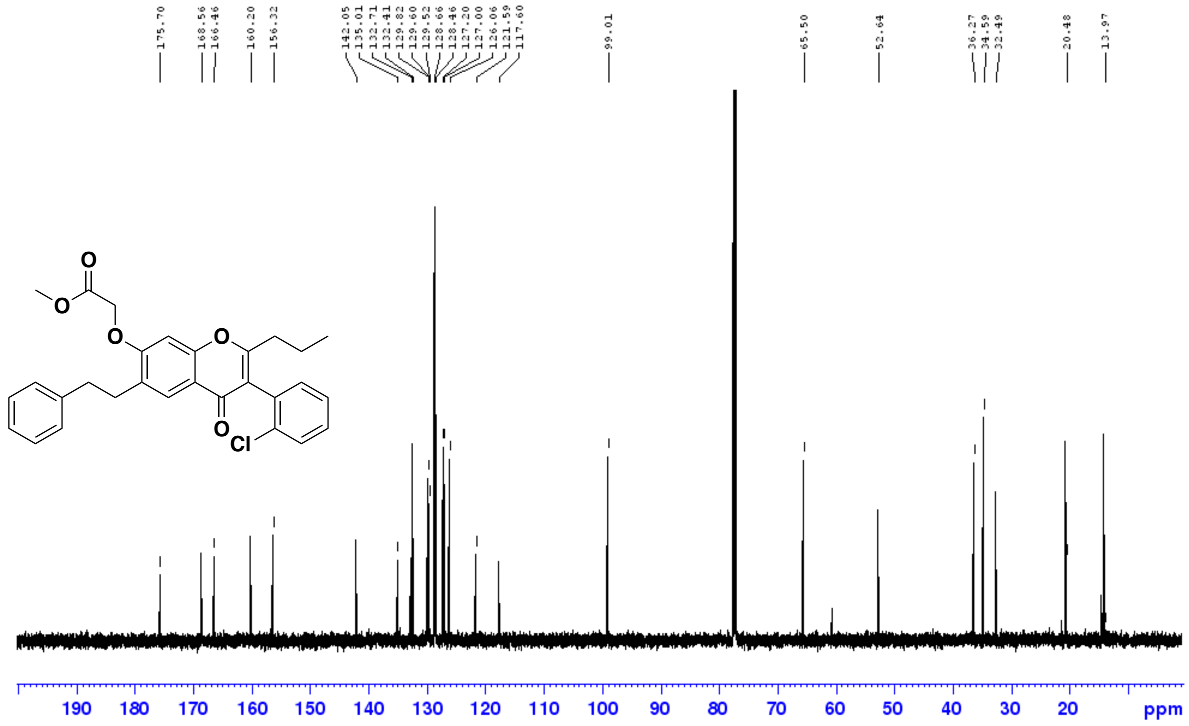
175.70 168.56 166.46 160.20 156.32 142.05 135.01 132.71 132.41 129.82 129.60 129.52 128.66 128.46 127.20 127.00 126.06 121.59 117.60 99.01 65.50 52.64 36.27 34.59 32.49 20.48 13.97
O
O
O
O
Cl
190 180 170 160 150 140 130 120 110 100 90 80 70 60 50 40 30 20 ppm

JG-48

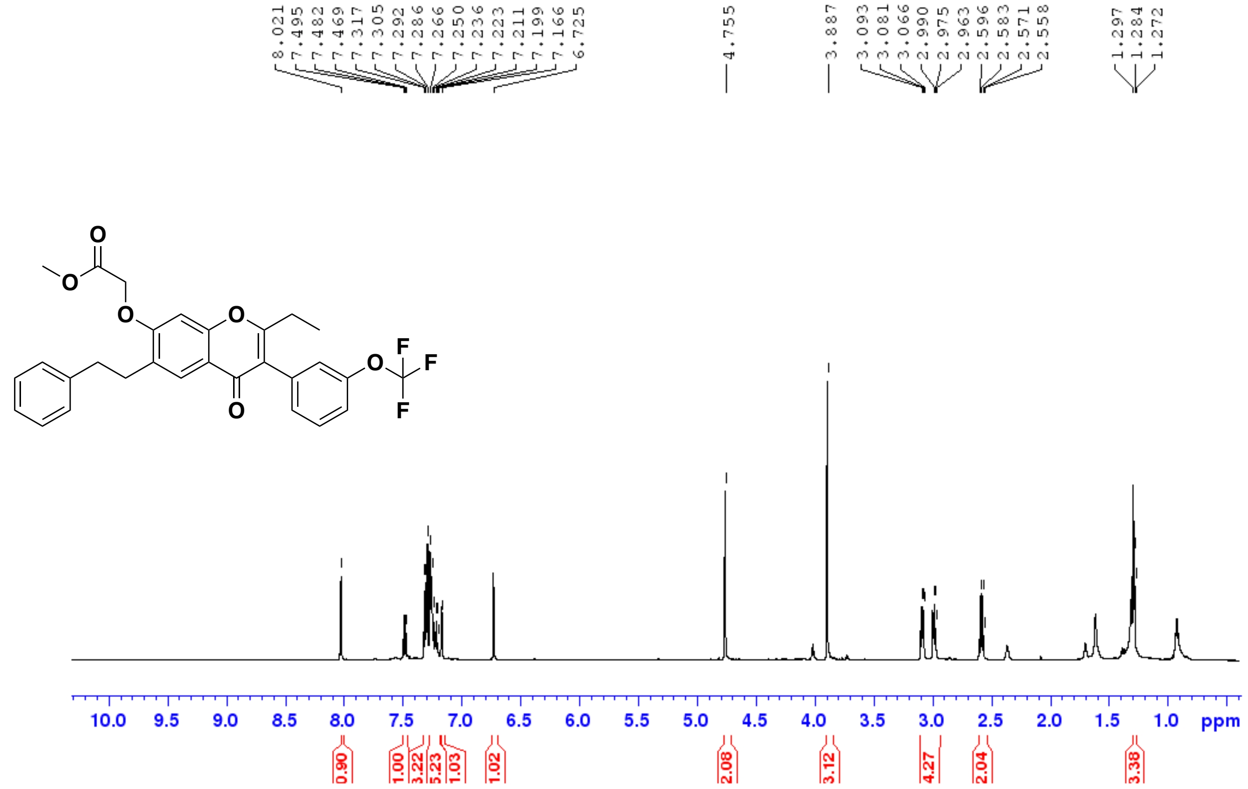
8.021
7.495
7.482
7.469
7.317
7.305
7.292
7.286
7.266
7.250
7.236
7.223
7.211
7.199
7.166
6.725
4.755
3.887
3.093
3.081
3.066
2.990
2.975
2.963
2.596
2.583
2.571
2.558
1.297
1.284
1.272
O
O
O
O
O
F
F
F
O
O
10.0
9.5
9.0
8.5
8.0
7.5
7.0
6.5
6.0
5.5
5.0
4.5
4.0
3.5
3.0
2.5
2.0
1.5
1.0
ppm
0.90
1.00
3.22
5.23
1.03
1.02
2.08
3.12
4.27
2.04
3.38

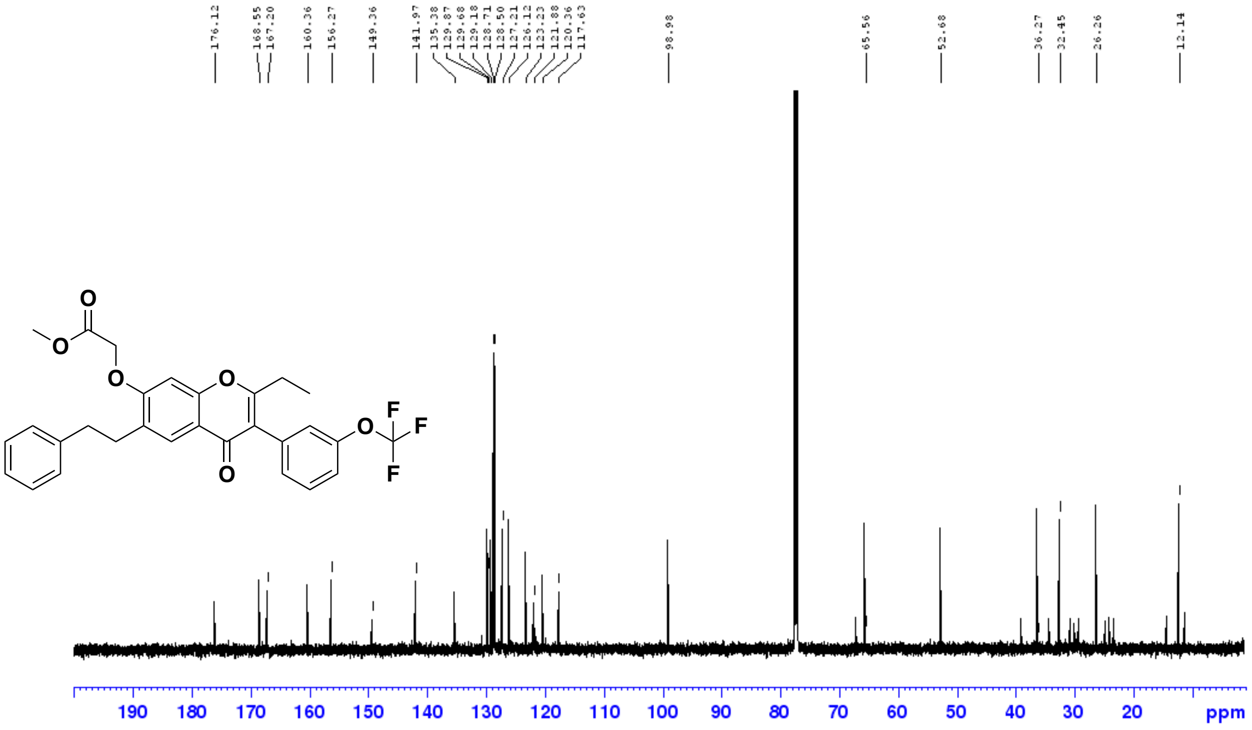
176.12
168.55
167.20
160.36
156.27
149.36
141.97
135.38
129.87
129.68
129.18
128.71
128.50
127.21
126.12
123.23
121.88
120.36
117.63
98.98
65.56
52.68
36.27
32.45
26.26
12.14
O
O
O
O
O
F
F
F
O
O
190
180
170
160
150
140
130
120
110
100
90
80
70
60
50
40
30
20
ppm

JG-49

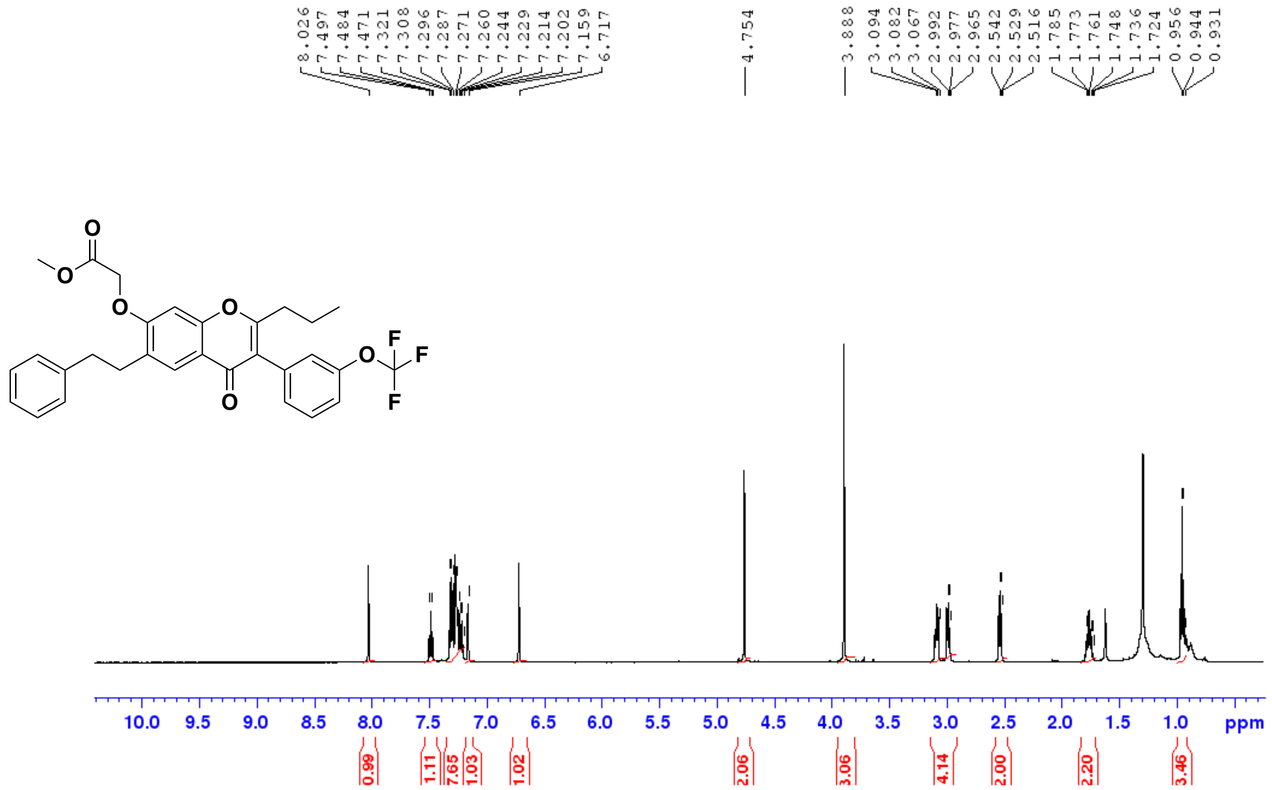
8.026
7.497
7.484
7.471
7.321
7.308
7.296
7.287
7.271
7.260
7.244
7.229
7.214
7.202
7.159
6.717
4.754
3.888
3.094
3.082
3.067
2.992
2.977
2.965
2.542
2.529
2.516
1.785
1.773
1.761
1.748
1.736
1.724
0.956
0.944
0.931
O
O
O
O
F
F
F
O
O
10.0 9.5 9.0 8.5 8.0 7.5 7.0 6.5 6.0 5.5 5.0 4.5 4.0 3.5 3.0 2.5 2.0 1.5 1.0 ppm
0.99
1.11
7.65
1.03
1.02
2.06
3.06
4.14
2.00
2.20
3.46

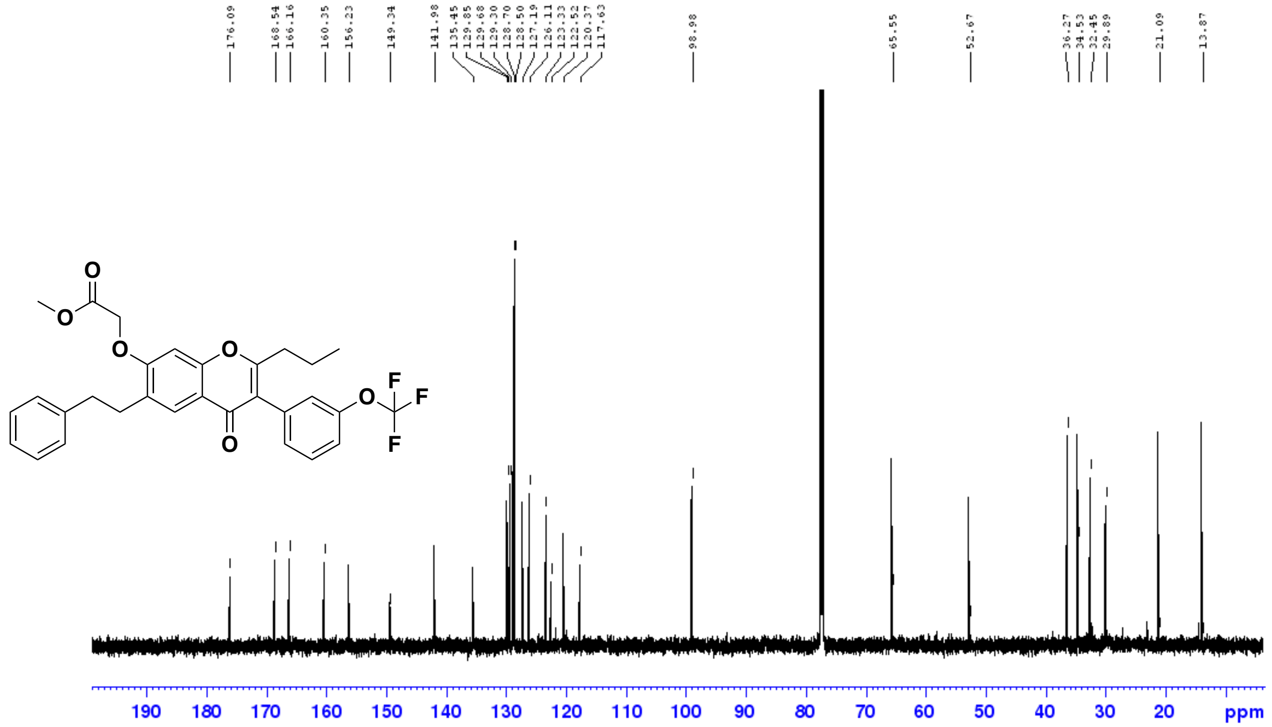
176.09
168.54
166.16
160.35
156.23
149.34
141.98
135.45
129.85
129.68
129.30
128.70
128.50
127.19
126.11
123.33
122.52
120.37
117.63
98.98
65.55
52.67
36.27
34.53
32.45
29.89
21.09
13.87
O
O
O
O
F
F
F
O
O
190 180 170 160 150 140 130 120 110 100 90 80 70 60 50 40 30 20 ppm
