# Supplementary material for: Zebrafish behavioural profiling identifies GABA and serotonin receptor ligands related to sedation and paradoxical excitation
Source: Nat Commun. 2019 Sep 9;10:4078. doi: 10.1038/s41467-019-11936-w (PMC6733874; doi:10.1038/s41467-019-11936-w)
Supplement: Supplementary file 2 — Description of Additional Supplementary Files [file 41467_2019_11936_MOESM2_ESM.docx]

**Description of Additional Supplementary Files**

File Name: Supplementary Movie 1

Description: **Control animals respond to violet light stimuli.** The video shows a group DMSO-treated zebrafish (negative controls) responding to violet light. Video was captured at 1000fps over a 0.5s time period in a single well of a 96 well plate. The violet light turns on at 250ms, as indicated.

File Name: Supplementary Movie 2

Description: **Control animals do not respond to low-volume acoustic stimuli.** The video shows a group of DMSO-treated zebrafish (negative controls) responding to an acoustic stimulus. Video was captured at 1000fps over a 0.5s time period in a single well of a 96 well plate. The acoustic stimulus (soft tap) occurs at 250ms, as indicated.

File Name: Supplementary Movie 3

Description: **Enhanced acoustic startle response in etomidate-treated animals.** The video shows a group of etomidate-treated zebrafish (6.0 μM) responding to an acoustic stimulus. Video was captured at 1000fps over a 0.5s time period in a single well of a 96 well plate. The acoustic stimulus (soft tap) occurs at 250ms, as indicated.

File Name: Supplementary Movie 4

Description: **Enhanced acoustic startle response in propofol-treated animals.** The video shows a group of etomidate-treated zebrafish (10 μM) responding to an acoustic stimulus. Video was captured at 1000fps over a 0.5s time period in a single well of a 96 well plate. The acoustic stimulus (soft tap) occurs at 250ms, as indicated.

File Name: Supplementary Movie 5

Description: **The enhanced acoustic startle response in different sized groups**. Animals, in different sized groups (1, 2, 4, 8, 16, 32), were plated in a 96 well plate. The left half of the plate was treated with vehicle, the right half with etomidate (6 µM). The video clip shows how the animals responded to an acoustic stimulus. Note that the animals respond as individuals, and in groups.
