# Supplementary material for: Zebrafish behavioural profiling identifies GABA and serotonin receptor ligands related to sedation and paradoxical excitation
Source: Nat Commun. 2019 Sep 9;10:4078. doi: 10.1038/s41467-019-11936-w (PMC6733874; doi:10.1038/s41467-019-11936-w)
Supplement: Supplementary file 9 — Reporting Summary [file 41467_2019_11936_MOESM9_ESM.pdf]

nature research

Corresponding author(s): *Double-blind peer review submissions: write DBPR and your manuscript number here instead of author names.*

Last updated by author(s): *YYYY-MM-DD*

Reporting Summary

Nature Research wishes to improve the reproducibility of the work that we publish. This form provides structure for consistency and transparency in reporting. For further information on Nature Research policies, see Authors & Referees and the Editorial Policy Checklist.

Statistics

For all statistical analyses, confirm that the following items are present in the figure legend, table legend, main text, or Methods section.

n/a	Confirmed	
☐	☒	The exact sample size (*n*) for each experimental group/condition, given as a discrete number and unit of measurement
☐	☒	A statement on whether measurements were taken from distinct samples or whether the same sample was measured repeatedly
☐	☒	The statistical test(s) used AND whether they are one- or two-sided<br>*Only common tests should be described solely by name; describe more complex techniques in the Methods section.*
☒	☐	A description of all covariates tested
☐	☒	A description of any assumptions or corrections, such as tests of normality and adjustment for multiple comparisons
☐	☒	A full description of the statistical parameters including central tendency (e.g. means) or other basic estimates (e.g. regression coefficient) AND variation (e.g. standard deviation) or associated estimates of uncertainty (e.g. confidence intervals)
☐	☒	For null hypothesis testing, the test statistic (e.g. *F*, *t*, *r*) with confidence intervals, effect sizes, degrees of freedom and *P* value noted<br>*Give P values as exact values whenever suitable.*
☒	☐	For Bayesian analysis, information on the choice of priors and Markov chain Monte Carlo settings
☒	☐	For hierarchical and complex designs, identification of the appropriate level for tests and full reporting of outcomes
☐	☒	Estimates of effect sizes (e.g. Cohen's *d*, Pearson's *r*), indicating how they were calculated

Our web collection on statistics for biologists contains articles on many of the points above.

Software and code

Policy information about availability of computer code

Data collection	Behavioral data were acquired out using custom scripts in Matlab (MathWorks) and Python. Data was stored in a custom in-house database.
Data analysis	Data analysis was carried out using standard scripts in Prism (GraphPad), and custom scripts in Matlab (MathWorks), and Python (e.g. numpy, scipy, and seaborn). SEA (Python code) is referenced in Keiser MJ, Roth BL, Armbruster BN, Ernsberger P, Irwin JJ, Shoichet BK. Relating protein pharmacology by ligand chemistry. Nat Biotech 25 (2), 197-206 (2007). Custom Python code was used to perform simulated annealing.

For manuscripts utilizing custom algorithms or software that are central to the research but not yet described in published literature, software must be made available to editors/reviewers. We strongly encourage code deposition in a community repository (e.g. GitHub). See the Nature Research guidelines for submitting code & software for further information.

Data

Policy information about availability of data

All manuscripts must include a data availability statement. This statement should provide the following information, where applicable:

- Accession codes, unique identifiers, or web links for publicly available datasets
- A list of figures that have associated raw data
- A description of any restrictions on data availability

The source data for all Figures and Supplementary Figures in the current study are available in the Zenodo repository, https://zenodo.org. 10.5281/zenodo.3336616

Field-specific reporting

Please select the one below that is the best fit for your research. If you are not sure, read the appropriate sections before making your selection.

☒ Life sciences ☐ Behavioural & social sciences ☐ Ecological, evolutionary & environmental sciences

For a reference copy of the document with all sections, see nature.com/documents/nr-reporting-summary-flat.pdf

Life sciences study design

All studies must disclose on these points even when the disclosure is negative.

Sample size	Sample sizes were chosen based on our ability to generate reproducible and statistically significant differences between positive and negative control groups.
Data exclusions	No data were excluded from the analysis.
Replication	All data were collected in replicate, and reproduced as described in the text and statistical analyses
Randomization	Zebrafish were randomly allocated into 96-well plates from large groups of larvae.
Blinding	In the FLIPR assay, compounds were blinded from the experimenter by labeling the tubes with a number code. Blinding was not used in the screen because compounds were uniformly transferred from the library plates. Similarly, the other behavioral assays were also not blinded. One reason is that data acquisition was automated, reducing opportunities for biased scoring. It is theoretically possible that biases could have been introduced at the pipetting step, for example, if a researcher subconsciously altered pipetting accuracy for certain compounds, but this possibility is unlikely due to the use of calibrated pipettemans. All the behavioral experiments were done with large numbers of randomly assigned animals, in many replicate wells, and on multiple days to provide convincing evidence that reported effects were due to the intended variables, rather than unintended bias. Also, the analyses of large-data sets were uniform, reducing the likelihood of unintentional bias.

Reporting for specific materials, systems and methods

We require information from authors about some types of materials, experimental systems and methods used in many studies. Here, indicate whether each material, system or method listed is relevant to your study. If you are not sure if a list item applies to your research, read the appropriate section before selecting a response.

Materials & experimental systems

n/a	Involved in the study	
☐	☒	Antibodies
☐	☒	Eukaryotic cell lines
☒	☐	Palaeontology
☐	☒	Animals and other organisms
☒	☐	Human research participants
☒	☐	Clinical data

Methods

n/a	Involved in the study	
☒	☐	ChIP-seq
☒	☐	Flow cytometry
☒	☐	MRI-based neuroimaging

Antibodies

Antibodies used	α-5HT (1:500, ImmunoStar), α-tERK (1:750, Cell Signaling), α-pERK (1:750, Cell Signaling)
Validation	All of the antibodies used in this study have been previously validated for use in zebrafish whole-mount immunoflourescence, α-5HT(PMID: 20107084), α-tERK & α-pERK(PMID: 26778924)

Eukaryotic cell lines

Policy information about cell lines

Cell line source(s)	HEK293 (ATCC, Cat. No. CRL-1573)
Authentication	HEK293 cell has been obtained from ATCC and used till 20 passages with continuous monitoring of cell morphology.
Mycoplasma contamination	HEK293 cell used here was not tested for mycoplasma
Commonly misidentified lines (See ICLAC register)	No commonly misidentified cell lines were used.

Animals and other organisms

Policy information about studies involving animals; ARRIVE guidelines recommended for reporting animal research

Laboratory animals	Species: Danio rerio. Strain: wild-type zebrafish from Singapore. Sex: There is no known way to determine the sex of larval Zebrafish. Age: Zebrafish were studied at 7 days post fertilization, unless otherwise noted in the text
Wild animals	The study did not involve wild animals.
Field-collected samples	The study did not include field-collected samples.
Ethics oversight	All zebrafish procedures were and approved by the UCSF's Institutional Animal Care Use Committee (IACUC), and in accordance with the Guide to Care and Use of Laboratory Animals (National Institutes of Health 1996) and conducted according to established protocols that complied with ethical regulations for animal testing and research.

Note that full information on the approval of the study protocol must also be provided in the manuscript.
